# Discovery of Small Molecule COX-1 and Akt Inhibitors as Anti-NSCLC Agents Endowed with Anti-Inflammatory Action

**DOI:** 10.3390/ijms24032648

**Published:** 2023-01-31

**Authors:** Mehlika Dilek Altıntop, Gülşen Akalın Çiftçi, Nalan Yılmaz Savaş, İpek Ertorun, Betül Can, Belgin Sever, Halide Edip Temel, Özkan Alataş, Ahmet Özdemir

**Affiliations:** 1 Department of Pharmaceutical Chemistry, Faculty of Pharmacy, Anadolu University, 26470 Eskişehir, Turkey; 2 Department of Biochemistry, Faculty of Pharmacy, Anadolu University, 26470 Eskişehir, Turkey; 3 Graduate School of Health Sciences, Anadolu University, 26470 Eskişehir, Turkey; 4 Department of Medical Biochemistry, Faculty of Medicine, Eskisehir Osmangazi University, 26480 Eskişehir, Turkey

**Keywords:** Akt, anti-inflammatory action, COX-1, hydrazones, non-small cell lung cancer, thiosemicarbazides

## Abstract

Targeted therapies have come into prominence in the ongoing battle against non-small cell lung cancer (NSCLC) because of the shortcomings of traditional chemotherapy. In this context, indole-based small molecules, which were synthesized efficiently, were subjected to an in vitro colorimetric assay to evaluate their cyclooxygenase (COX) inhibitory profiles. Compounds **3b** and **4a** were found to be the most selective COX-1 inhibitors in this series with IC_50_ values of 8.90 µM and 10.00 µM, respectively. In vitro and in vivo assays were performed to evaluate their anti-NSCLC and anti-inflammatory action, respectively. 2-(1*H*-Indol-3-yl)-*N*′-(4-morpholinobenzylidene)acetohydrazide (**3b**) showed selective cytotoxic activity against A549 human lung adenocarcinoma cells through apoptosis induction and Akt inhibition. The in vivo experimental data revealed that compound **3b** decreased the serum myeloperoxidase and nitric oxide levels, pointing out its anti-inflammatory action. Moreover, compound **3b** diminished the serum aminotransferase (particularly aspartate aminotransferase) levels. Based on the in vitro and in vivo experimental data, compound **3b** stands out as a lead anti-NSCLC agent endowed with in vivo anti-inflammatory action, acting as a dual COX-1 and Akt inhibitor.

## 1. Introduction

Non-small cell lung cancer (NSCLC), which accounts for the majority (~85%) of lung cancer cases, is by far the primary cause of cancer-related death throughout the world [1]. Despite significant advances in both diagnosis and treatment, the prognosis for patients with NSCLC still remains poor and the 5-year survival rates of the patients are very low [2]. Surgery, chemotherapy, radiotherapy, immunotherapy, and targeted therapy are existing treatment modalities for NSCLC [3]. Clinical outcomes of patients with NSCLC depend on the cancer stage at the time of diagnosis [4]. The early stages of NSCLC carry the maximum potential for therapeutic intervention and, therefore, its early detection is critical for managing the disease and improving the survival rate [4]. However, there are many challenges in the diagnosis of NSCLC, as it is often asymptomatic early in its course [5,6].

The best treatment option for early stage NSCLC continues to be surgical resection. When the disease is diagnosed at an advanced stage, surgical intervention is no longer an option [7]. In this case, radiotherapy and chemotherapy (e.g., platinum-based chemotherapy) become major therapeutic approaches for unresectable NSCLC [1]. Despite their benefits in NSCLC therapy, conventional chemotherapeutic agents destroy normal cells along with cancer cells and, therefore, these drugs cause severe toxicity and adverse effects [8,9]. Two major barriers to NSCLC management are resistance to radio(chemo)therapy and metastasis [1,9], both of which are the main causes of NSCLC-related mortality [10,11].

The above-mentioned drawbacks have shifted the paradigm of cancer therapy from traditional chemotherapy to targeted therapy, a milestone approach that aims to maximize therapeutic benefits with negligible side effects [3].

The lungs are particularly prone to injury and inflammation since the lungs are continuously exposed to the external environment [12]. Mounting evidence has demonstrated the causal link between chronic inflammation and lung cancer. According to epidemiological data, approximately 20% of cancer-related deaths are associated with unabated inflammation [13]. Chronic inflammation plays a multifaceted role in carcinogenesis; conversely, cancer can also lead to inflammation [12]. Inflammation predisposes to the development of lung cancer [14] and can contribute to tumor initiation, promotion, progression, and metastasis [15]. Targeting inflammation stands out as a rational strategy not only for cancer therapy but also for cancer prevention [16]. Nonsteroidal anti-inflammatory drugs (NSAIDs) significantly diminish the risk of developing certain types of cancer (e.g., colon, lung, breast, and prostate cancer) by reducing tumor-related inflammation [13]. Long-term aspirin use has been reported to reduce the incidence and mortality associated with several cancer types. Several possible mechanisms have been suggested to explain the link between NSAID use and cancer prevention. One of those is cyclooxygenase (COX) inhibition, which reduces the production of inflammatory mediators, particularly prostaglandins (PGs) [16].

COX-1 expression has been reported to be up-regulated in tumorigenesis [17] and implicated in multiple aspects of cancer pathophysiology and, therefore, the inhibition of COX-1, by a variety of selective and nonselective inhibitors, is an emerging approach for pharmacologic intervention in cancer. However, there is only one selective COX-1 inhibitor currently prescribed as an NSAID (mofezolac), just in Japan, for the management of pain and inflammation [17,18,19,20].

Akt, also known as protein kinase B (PKB), is one of the most frequently hyperactivated protein kinases in a variety of human cancers including NSCLC [21,22,23]. Akt overactivation affects several downstream effectors and mediates multiple pathways that promote tumorigenesis (e.g., cell survival, growth, and proliferation) [21]. Furthermore, the hyperactivation of Akt intrinsically up-regulates the nuclear factor-κB (NF-κB) pathway, which transcriptionally initiates pro-inflammatory networks to build up the inflammatory tumor microenvironment [24]. Although diverse small molecule Akt inhibitors have been entered in clinical trials, none of them have been approved [25].

Hydrazides-hydrazones are not only versatile intermediates for the synthesis of various heterocyclic compounds but also commonly occurring motifs in drug-like molecules because of their unique features (e.g., serving as both H-bond donors and acceptors) and diverse pharmacological applications for the management of microbial infections, cancer, and inflammation [26,27,28,29]. Hydrazones exert pronounced antitumor action through diverse mechanisms including apoptosis induction, cell cycle arrest, angiogenesis inhibition, and inhibition of a plethora of biological targets related to the pathogenesis of cancer, including Akt [29,30,31,32,33,34,35,36]. Moreover, mounting evidence has demonstrated the anti-inflammatory and/or COX inhibitory potential of hydrazones [37,38,39,40,41].

Thiosemicarbazides are sulfur and nitrogen-containing ligands distinguished by their capability to form complexes with transition metals (e.g., iron, zinc, and copper) [42]. Thiosemicarbazides have aroused great interest not only as intermediates for the synthesis of biologically active heterocycles but also privileged motifs in many bioactive pharmaceutical products [42,43,44,45]. Thiosemicarbazides/thiosemicarbazones show a wide range of pharmacological activities ranging from anticancer activity to anti-inflammatory potency due to their unique structural features, allowing them to interact with the pivotal residues of biological targets associated with the pathogenesis of many diseases, particularly cancer and inflammation [42,43,44,45,46,47,48,49,50,51,52,53,54,55,56]. Triapine, a synthetic thiosemicarbazone, is a small molecule antineoplastic agent endowed with ribonucleotide reductase (RNR) inhibitory activity [42,43,44,45].

**Figure 1:**
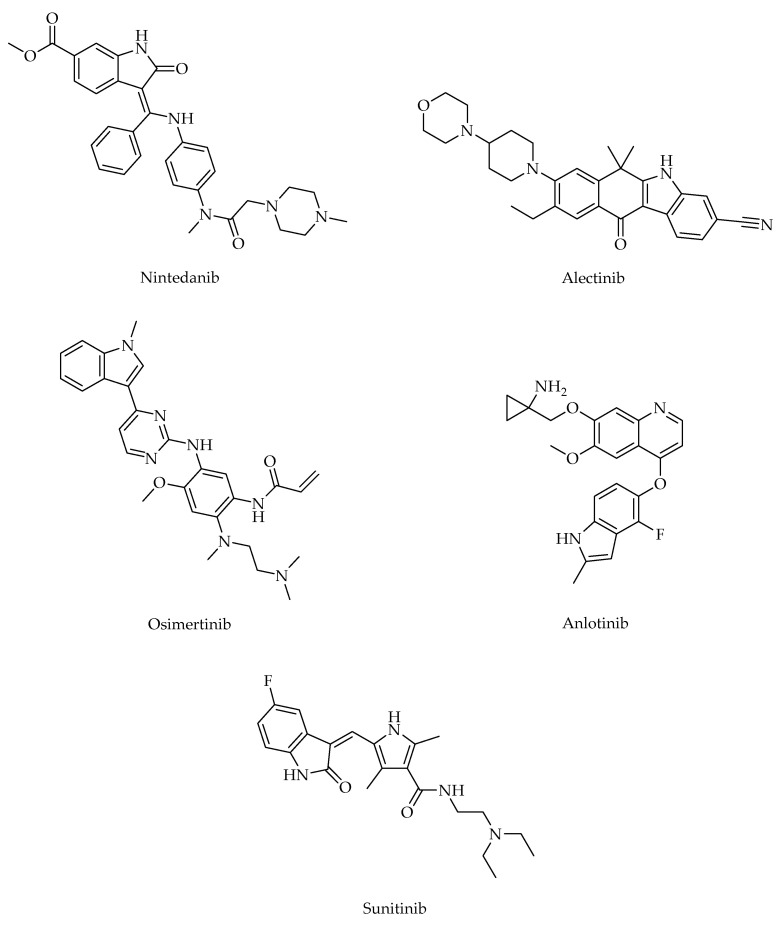
Indole-based anti-NSCLC agents.

**Figure 2:**
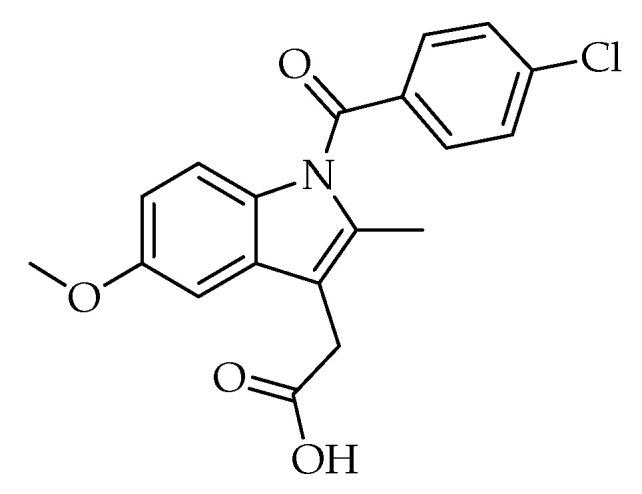
Indomethacin.

The indole ranks among the top 25 most common nitrogen heterocycles in U.S. Food and Drug Administration (FDA)-approved drugs. It is also a key structural component of an essential amino acid (tryptophan), a monoamine neurotransmitter (serotonin), and countless natural products (e.g., vinca alkaloids) [57]. The diverse applications of the indole core in challenging diseases (e.g., lung cancer, inflammatory diseases) make it one of the most privileged heterocyclic scaffolds for drug discovery [57,58]. Among vinca alkaloids, vinorelbine is the most frequently used antimitotic drug to treat lung cancer and vinblastine, in combination with cisplatin, is used in the management of NSCLC. Nintedanib (in combination with docetaxel), alectinib, osimertinib, anlotinib, and sunitinib are indole-based anti-NSCLC agents (Figure 1) [59]. In general, indole derivatives have been reported to exert marked anti-NSCLC action through diverse mechanisms including the induction of apoptosis, the inhibition of crucial biological targets such as microtubule, topoisomerases, protein kinases (e.g., Akt), and histone deacetylases (HDACs) [58,59,60,61,62]. The indole is also considered to be one of the most eligible scaffolds for anti-inflammatory drug discovery [62,63,64,65,66,67]. Indomethacin (Figure 2) is one of the most commonly prescribed NSAIDs exerting its action through the inhibition of COXs. Moreover, several experimental studies have revealed that indomethacin shows significant antiproliferative activity against a broad array of cancer (e.g., colorectal, lung) cell lines [68,69,70,71,72].

Taken together, the aforementioned data [26,27,28,29,30,31,32,33,34,35,36,37,38,39,40,41,42,43,44,45,46,47,48,49,50,51,52,53,54,55,56,57,58,59,60,61,62,63,64,65,66,67,68,69,70,71,72] prompted us to design two classes of indole-based small molecules (**3a-j**, **4a-g**) for the targeted therapy of NSCLC. In this context, we performed the synthesis of new hydrazones (**3a-j**) and thiosemicarbazides (**4a-g**) efficiently and conducted in vitro and in vivo assays to assess their potential for the targeted therapy of NSCLC.

## 2. Results

**Figure 3:**
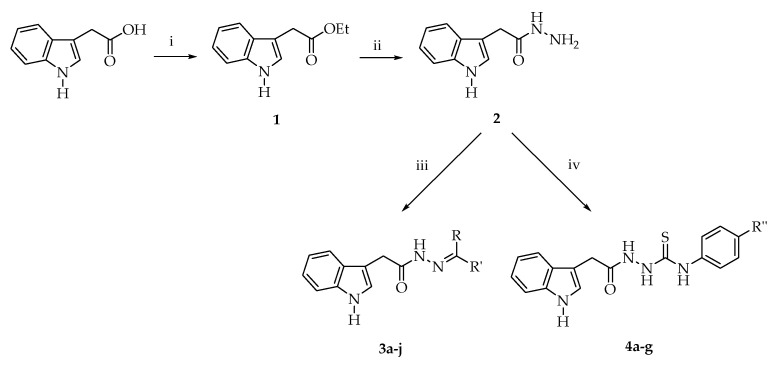
The synthetic route for the preparation of compounds **3a-j** and **4a-g**. Reagents and conditions: (i) EtOH, H_2_SO_4_, reflux, 12 h; (ii) NH_2_NH_2_.H_2_O, EtOH, reflux, 4 h; (iii) RCHO or RCOR′, EtOH, reflux, 15 h; (iv) R″C_6_H_4_NCS, EtOH, rt, 8 h.

The reaction sequence for the preparation of the hitherto unreported small molecules (**3a-j**, **4a-g**) is depicted in Figure 3, starting from 2-(1*H*-indol-3-yl)acetic acid. The convenient and efficient reaction of compound **2** with aromatic aldehydes or ketones and aryl isothiocyanates yielded new hydrazones (**3a-j**), and thiosemicarbazides (**4a-g**), respectively.

New hydrazones (**3a-j**) and thiosemicarbazides (**4a-g**) were subjected to in vitro assays to determine their COX inhibitory profiles. Among compounds **3a-j**, compound **3a** was found to be a nonselective COX inhibitor with IC_50_ values of 10.35 µM and 12.50 µM for COX-1 and COX-2, respectively (Table 1). On the other hand, compound **3b** was the most selective COX-1 inhibitor (IC_50_ = 8.90 µM) in this series with a selectivity index (SI) value of 0.13.

Among compounds **4a-g**, compound **4a** was the most selective COX-1 inhibitor (IC_50_ = 10.00 µM) (Table 2). Other compounds did not show any inhibitory potency on COX-1 at the tested concentrations.

**Figure 4:**
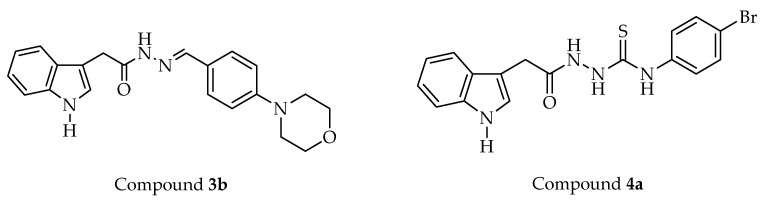
Selective COX-1 inhibitors in this series.

All compounds were examined for their cytotoxic effects on L929 mouse fibroblast (normal) cells using the MTT test. Based on the in vitro experimental data, compound **3a**, the nonselective COX inhibitor, showed cytotoxicity toward L929 cells with an IC_50_ value of 17.33 µM (Table 3), which is close to its IC_50_ values indicated in Table 1. On the other hand, compounds **3b** and **4a** did not show cytotoxicity against L929 cells at their effective concentrations. As a result, compounds **3b** and **4a** (Figure 4), the selective COX-1 inhibitors in this series, were chosen for further studies.

Compounds **3b** and **4a** were also subjected to the MTT assay to assess their cytotoxicity toward A549 human lung adenocarcinoma cell line. Based on the data presented in Table 4, compound **3b** was found to be the most potent anticancer agent on A549 cells with an IC_50_ value of 89.67 µM compared to cisplatin (IC_50_ = 22.67 µM). On the other hand, compound **4a** showed cytotoxic activity against A549 cells with an IC_50_ value of 179.33 µM.

**Figure 5:**
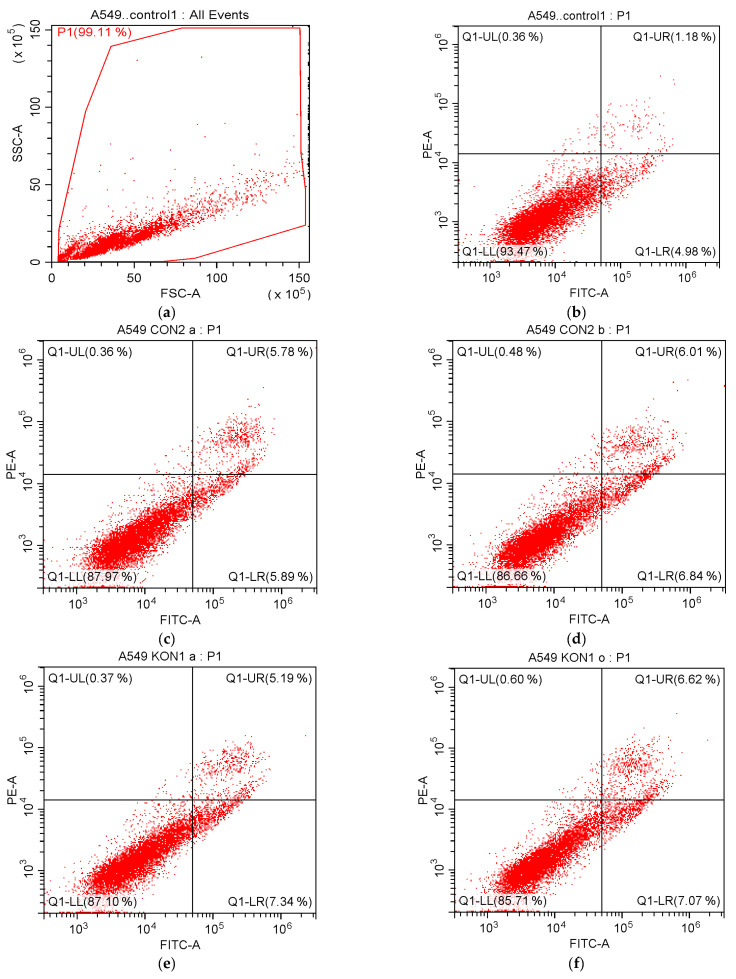

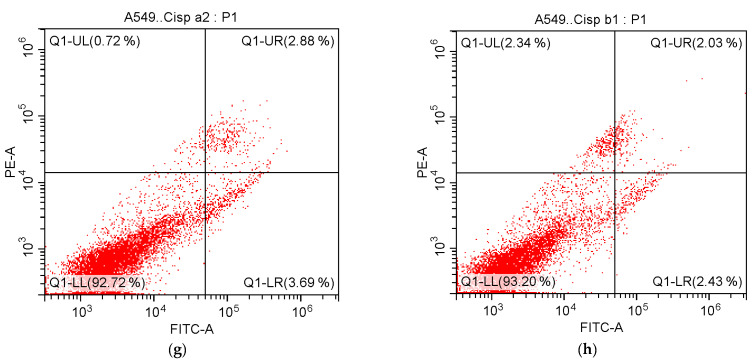
Flow cytometric analysis of A549 cells treated with IC_50_/2 and IC_50_ concentrations of compounds **3b**, **4a,** and cisplatin. At least 10,000 cells were analyzed per sample, and quadrant analysis was performed. Q1-LR, Q1-UR, Q1-LL, and Q1-UL quadrants represent early apoptosis, late apoptosis, viability, and necrosis, respectively. (**a**) Control; (**b**) Control; (**c**) Compound **3b** at IC_50_/2 concentration; (**d**) Compound **3b** at IC_50_ concentration; (**e**) Compound **4a** at IC_50_/4 concentration; (**f**) Compound **4a** at IC_50_/2 concentration; (**g**) Cisplatin at IC_50_/2 concentration; (**h**) Cisplatin at IC_50_ concentration.

After 24 h incubation of A549 cells treated with compounds **3b** and **4a** in this series and cisplatin, flow cytometry-based apoptosis detection assay was performed to identify early and late apoptotic cells using Annexin V-fluorescein isothiocyanate (FITC)/propidium iodide (PI) staining. The percentages of A549 cells undergoing apoptosis (early and late) exposed to compounds **3b** and cisplatin at their IC_50_/2 concentrations were found to be 11.67% and 6.57%, respectively. On the other hand, the percentages of A549 cells undergoing apoptosis (early and late) exposed to compounds **3b** and cisplatin at their IC_50_ concentrations were 12.85% and 4.46%, respectively (Table 5, Figure 5). The percentages of A549 cells undergoing early and late apoptosis exposed to compound **4a** at its IC_50_/4 concentration were 7.34% and 5.19%, respectively. On the other hand, the percentages of A549 cells undergoing early and late apoptosis exposed to compound **4a** at its IC_50_/2 concentration were found to be 7.07% and 6.62%, respectively (Table 5, Figure 5).

Akt inhibition caused by compounds **3b** and **4a** in A549 cells was examined using a colorimetric assay. Compounds **3b** and **4a** caused Akt inhibition in A549 cell line with IC_50_ values of 32.50 and 45.33 µM as compared to GSK690693 (IC_50_= 5.93 µM) (Table 6).

The lipopolysaccharide (LPS)-induced sepsis model was used to assess the in vivo anti-inflammatory activities of compounds **3b** and **4a**. According to the data indicated in Table 7, the myeloperoxidase (MPO) activity of the LPS group increased as compared to the control group. However, this increase is not statistically significant. The LPS + compound **3b** group slightly decreased the MPO activity compared to the LPS group, while the LPS + compound **4a** group significantly decreased the MPO activity compared to the LPS group (*p* < 0.05). The decrease in the MPO activity caused by compound **4a** was higher than that caused by the indomethacin therapy.

As presented in Table 8, there was a significant increase in the nitric oxide (NO) level after LPS administration compared to the control (*p* < 0.001). LPS + compound **3b**, LPS + compound **4a**, and LPS + indomethacin caused a significant decrease in the serum NO levels. However, this decrease in LPS + compound **3b** was similar to the control. The NO level was significantly higher in the LPS group than in the control group, while it was markedly lower in the compound **4a** pre-treatment group compared to the LPS group (*p* < 0.05).

According to the in vivo experiments, the alanine aminotransferase (ALT) level decreased in all groups compared to the LPS group (Table 9). This decrease was greater in the group treated with compound **4a** compared to the group treated with compound **3b**. Likewise, the aspartate aminotransferase (AST) level decreased in all groups compared to the LPS group. This decrease was greater in the group treated with compound **3b** compared to the group treated with compound **4a** and the group treated with indomethacin. However, the decrease caused by the compounds in the ALT and AST levels was not statistically significant compared to the LPS group.

## 3. Discussion

Experimental studies have demonstrated that hydrazones show marked antitumor action through various mechanisms, including the inhibition of Akt [31] or the phosphatidylinositol 3-kinase (PI3K)/Akt signaling pathway [32,36]. *N*′-benzylidene-2-[(4-(4-methoxyphenyl)pyrimidin-2-yl)thio]acetohydrazide was previously reported to exert marked anticancer activity against the 5RP7 H-*ras* oncogene transformed rat embryonic fibroblast cell line via the induction of apoptosis and the inhibition of Akt (IC_50_ = 0.50 µg/mL) [31]. According to western blot data reported by Han et al., (*S*)-2-{[5-[1-(6-methoxynaphtalene-2-yl)ethyl]-4-(4-fluorophenyl)-4*H*-1,2,4-triazole-3-yl]thio}-*N*′-[(5-nitrofuran-2-yl)methylidene]acetohydrazide caused a significant decrease in the epidermal growth factor receptor (EGFR), PI3K, and Akt phosphorylation in PC3 human prostate cancer cells [32]. Bak et al. indicated that 5-hydroxy-7,4′-diacetyloxyflavanone-*N*-phenyl hydrazone (N101-43) induced apoptosis via the up-regulation of Fas/FasL expression, the activation of caspase cascade, and the inhibition of the PI3K/Akt signaling pathway in NSCLC cells [36].

The anti-inflammatory and/or COX inhibitory potential of hydrazones was demonstrated by in vitro and in vivo studies [37,38,39,40,41]. In our previous work, 2-[(1-methyl-1*H*-tetrazol-5-yl)thio]-*N*′-(4-(piperidin-1-yl)benzylidene)acetohydrazide and 2-[(1-methyl-1*H*-tetrazol-5-yl)thio]-*N*′-(4-(morpholin-4-yl)benzylidene)acetohydrazide caused selective COX-1 inhibition [37].

Thiosemicarbazides show pronounced antiproliferative activity toward a variety of tumor cells through diverse mechanisms [42,43,44,45,46,47,48,49,50,51,52]. Our research team reported that 4-(1,3-benzodioxol-5-yl)-1-([1,1′-biphenyl]-4-ylmethylene)thiosemicarbazide showed remarkable anticancer activity against A549 human lung adenocarcinoma and C6 rat glioma cells through apoptosis induction mediated by the disruption of ΔΨm [52].

In vitro and in vivo experimental data revealed that thiosemicarbazides exert marked anti-inflammatory action through several mechanisms including COX inhibition [53,54,55,56]. In our recent work [53], 4-[4-(piperidin-1-ylsulfonyl)phenyl]-1-[4-(4-cyanophenoxy)benzylidene]thiosemicarbazide was found to be a selective COX-1 inhibitor with an IC_50_ value of 1.89 µM. On the other hand, 4-[4-(piperidin-1-ylsulfonyl)phenyl]-1-[4-(4-nitrophenoxy)benzylidene]thiosemicarbazide was determined to be a nonselective COX inhibitor (COX-1 IC_50_ = 13.44 µM, COX-2 IC_50_ = 12.60 µM). Based on the LPS-induced sepsis model, these agents diminished the MPO, NO, high-sensitivity C-reactive protein (hsCRP), malondialdehyde (MDA), ALT, and AST levels. Both compounds were identified as potential anti-inflammatory agents [53].

Indole-based small molecules exert a notable anti-NSCLC action through multiple mechanisms such as the induction of apoptosis and the inhibition of crucial biological targets including protein kinases (e.g., Akt) [58,59,60,61,62]. Furthermore, mounting evidence has demonstrated that indole derivatives show marked anti-inflammatory action via COX inhibition [62,63,64,65,66,67]. In our previous study [65], 3-(5-bromo-1*H*-indol-3-yl)-1-(4-cyanophenyl)prop-2-en-1-one was found to be a nonselective COX inhibitor (COX-1 IC_50_ = 8.10 µg/mL, COX-2 IC_50_ = 9.50 µg/mL), while 3-(5-methoxy-1*H*-indol-3-yl)-1-(4-(methylsulfonyl)phenyl)prop-2-en-1-one inhibited COX-1 (IC_50_ = 8.60 µg/mL). According to the LPS-induced sepsis model, both compounds markedly decreased the MPO, NO, hsCRP, MDA, ALT, and AST levels. Both indole derivatives were identified as potential anti-inflammatory agents [65].

Based on the aforementioned studies [26,27,28,29,30,31,32,33,34,35,36,37,38,39,40,41,42,43,44,45,46,47,48,49,50,51,52,53,54,55,56,57,58,59,60,61,62,63,64,65,66,67,68,69,70,71,72], two classes of indole-based small molecules (**3a-j**, **4a-g**) for the targeted therapy of NSCLC were designed. In this context, we carried out the synthesis of new hydrazones (**3a-j**) and thiosemicarbazides (**4a-g**) efficiently and performed in vitro and in vivo experiments to assess their potential for the targeted therapy of NSCLC.

Among compounds **3a-j**, compound **3a** was determined to be a nonselective COX inhibitor with IC_50_ values of 10.35 µM and 12.50 µM for COX-1 and COX-2, respectively, while compound **3b** was found to be a selective COX-1 inhibitor (IC_50_ = 8.90 µM). Compound **3b** exhibited COX-2 inhibitory activity with an IC_50_ value of 71.00 µM. The SI values of compounds **3a** and **3b** were determined to be 0.83 and 0.13, respectively. In particular, the pyrrolidine ring enhanced the inhibitory effects on both COXs, whereas the morpholine substitution caused selective COX-1 inhibitory potency. The replacement of the morpholine ring (compound **3b**) with the piperidine ring (compound **3c**) or the piperazine ring (compound **3d**) led to a substantial drop in COX-1 inhibitory activity. Compound **3c**, carrying a piperidine ring, showed the lowest COX inhibition (>100 µM) in this series.

According to the in vitro data related to the inhibitory effects of compounds **3b** and **3h** on COXs, it can be concluded that the ethoxy linker between the morpholine and the benzene rings diminishes COX-1 inhibition, while it enhances COX-2 inhibition. Taking into account the inhibitory effects of compounds **3b** and **3g** on COXs, it is important to note that the methyl branching decreases COX-1 inhibition and increases COX-2 inhibition.

The SI values of compounds **3e** and **3f** were found to be 2.39 and 1.84, respectively, indicating that the methylsulfonyl group significantly enhances COX-2 selectivity.

Based on the experimental results related to the inhibitory effects of compounds **3i** and **3j** on COXs, the methyl substituent at the 1^st^ position of the indole scaffold enhances COX-1 selectivity, while the methoxy substituent at the 5^th^ position of the indole core enhances COX-2 selectivity.

Among compounds **4a-g**, the most selective COX-1 inhibitor was found to be compound **4a** (IC_50_ = 10.00 µM, SI = 0.13). It can be concluded that the bromo substituent at the 4th position of the phenyl moiety significantly enhanced the COX-1 inhibitory potency. Other compounds did not exhibit any inhibitory activity towards COX-1 at the tested concentrations. Thiosemicarbazides tested in this work, except for compound **4a**, were found to have a tendency to inhibit the COX-2 enzyme.

Among the indole-based small molecules (**3a-j**, **4a-g**), compounds **3b** and **4a**, selective COX-1 inhibitors in this series, were chosen for further studies since both compounds did not exert cytotoxicity toward L929 (normal) cells at their effective concentrations reported for their COX-1 inhibitory activity.

To investigate their potential as anti-NSCLC agents, their cytotoxic effects on A549 cells were evaluated by means of the MTT assay protocol. Based on the experimental data, compound **3b** was the most potent anticancer agent on A549 cell line with an IC_50_ value of 89.67 µM. It can be concluded that the anticancer activity of compound **3b** against A549 cells is selective since the IC_50_ value of compound **3b** for L929 cells is 176.67 µM. On the other hand, compound **4a** showed cytotoxic activity against A549 and L929 cells with IC_50_ values of 179.33 µM and 84.00 µM, respectively. The cytotoxic activity of compound **4a** toward A549 cells was found to be nonselective at its IC_50_ value. For this reason, the IC_50_/4 and IC_50_/2 concentrations of compound **4a** were applied in the flow cytometry analyses of apoptosis and the Akt inhibition assay.

In cancer, cells lose their ability to undergo apoptosis, resulting in uncontrolled proliferation [73]. The induction of apoptosis is reported to be an intriguing modality for the management of all types of cancer since apoptosis evasion is a hallmark of cancer and is nonspecific to the cause or the type of the cancer [74]. Based on the flow cytometry-based apoptosis detection assay performed in this work, A549 cells treated with compounds **3b** and **4a** underwent apoptosis, pointing out their apoptosis-inducing efficacy.

Akt participates in the pathogenesis of NSCLC and, therefore, the inhibition of Akt by natural and synthetic agents stands out as a rational strategy for cancer therapy [21,22,23,24,25]. The colorimetric assay conducted in this study revealed that the Akt inhibitory activity of compound **3b** (IC_50_ = 32.50 µM) was more notable than that of compound **4a** (IC_50_ = 45.33 µM) in A549 cells.

Sepsis is described as a life-threatening organ dysfunction provoked by a dysregulated host response to infection [53]. Inflammatory imbalance plays a fundamental role in the pathogenesis of sepsis and occurs throughout the whole process of sepsis [75]. The parameters related to inflammation are crucial for evaluating a sepsis case [76]. In this work, the LPS-induced sepsis model was used to evaluate the in vivo anti-inflammatory activities of compounds **3b** and **4a**.

MPO is linked to several diseases, particularly those in which strong infiltration of polymorphonuclear cells (PMNs) and acute or chronic inflammation are involved. MPO contributes to the pathophysiology of diverse diseases such as rheumatoid arthritis, atherosclerosis, pulmonary fibrosis, renal glomerular injury, multiple sclerosis, Huntington’s disease, Alzheimer’s disease, Parkinson’s disease, liver diseases, diabetes, obesity, and cancer. MPO is reported to promote tumor initiation and progression. MPO participates in the regulation of tumor growth, apoptosis, migration, and metastasis [77]. In this work, compound **3b** caused a slight decrease in the MPO activity compared to the LPS group, whereas compound **4a** significantly diminished the MPO activity compared to the LPS group (*p* < 0.05).

Sepsis is characterized by a robust rise in NO production throughout the body that is driven by inducible NO synthase (iNOS) [78]. Due to the key role of NO in the pathogenesis of inflammation as a signaling molecule [78,79,80], herein the effects of compounds **3b** and **4a** on the serum NO levels were evaluated. The in vivo experimental data revealed that compounds **3b** and **4a** diminished the serum NO levels.

Aminotransferases, also referred to as transaminases, are commonly used as markers of hepatocellular injury in nonclinical toxicology studies and clinical trials. In general, aminotransferase activity in blood (serum or plasma) is elevated in the hepatocellular damage induced by diseases or drugs such as anti-inflammatory drugs [81,82,83,84]. Based on the in vivo experimental data performed in this work, both compounds caused a decrease in the serum aminotransferase levels. In particular, compound **3b** diminished the serum AST level more than indomethacin.

Taking into account the knowledge obtained from the in vitro and in vivo assays, compound **3b** can be considered as a lead compound for the targeted therapy of NSCLC due to its direct cytotoxic effects on A549 cells as well as its possible effects on the tumor microenvironment (e.g., tumor-related inflammation).

## 4. Materials and Methods

### 4.1. Chemistry

The chemicals were procured from commercial suppliers and were used without further purification. Melting points (M.p.) were determined on the Electrothermal IA9200 digital melting point apparatus (Staffordshire, UK) and were uncorrected. Thin Layer Chromatography (TLC) was performed on TLC Silica gel 60 F254 aluminum sheets (Merck, Darmstadt, Germany) using petroleum ether:ethyl acetate solvent system (1:1). IR spectra were recorded on the IRPrestige-21 Fourier Transform Infrared spectrophotometer (Shimadzu, Tokyo, Japan). ^1^H and ^13^C NMR spectra were recorded on the Varian Mercury 400 NMR spectrometer (Agilent, Palo Alto, CA, USA). HRMS spectra were recorded on the LC/MS IT-TOF system (Shimadzu, Tokyo, Japan) using the electrospray ionization (ESI) technique.

#### 4.1.1. Preparation of ethyl 2-(1*H*-indol-3-yl)acetate (**1**)

Compound **1** was synthesized starting from 2-(1*H*-indol-3-yl)acetic acid according to a previous work [85].

#### 4.1.2. Preparation of 2-(1*H*-indol-3-yl)acetohydrazide (**2**)

Compound **2** was obtained by the reaction of compound **1** with hydrazine hydrate according to a previous work [85].

#### 4.1.3. General Method for the Preparation of *N*′-benzylidene/(1-arylethylidene)-2-(1*H*-indol-3-yl)acetohydrazide Derivatives (**3a-j**)

A mixture of compound **2** and aromatic aldehyde or ketone in ethanol was heated under reflux for 15 h. At the end of this period, the precipitate was filtered off and dried. The product was crystallized from ethanol.

2-(1*H*-Indol-3-yl)-*N*′-[4-(pyrrolidin-1-yl)benzylidene]acetohydrazide (**3a**)

Yield: 78%. M.p.: 302–303 °C. IR ν_max_ (cm^−1^): 3196.05, 3074.53, 3043.67, 2966.52, 2914.44, 2873.94, 2848.86, 1668.43, 1595.13, 1546.91, 1521.84, 1487.12, 1460.11, 1431.18, 1386.82, 1350.17, 1323.17, 1292.31, 1249.87, 1224.80, 1174.65, 1163.08, 1118.71, 1047.35, 1001.06, 983.70, 958.62, 929.69, 914.26, 856.39, 804.32, 719.45, 682.80. ^1^H NMR (400 MHz, DMSO-*d_6_*): 2.02–2.05 (m, 4H), 3.40–3.42 (m, 4H), 3.60 and 4.02 (2s, 2H), 6.92–7.07 (m, 4H), 7.21 (dd, *J =* 2.4 Hz, 12.8 Hz, 1H), 7.31–7.35 (m, 1H), 7.48–7.59 (m, 3H), 7.88 and 8.08 (2s, 1H), 10.85 and 10.88 (2s, 1H), 11.05 and 11.28 (2s, 1H). ^13^C NMR (100 MHz, DMSO-*d_6_*): 25.41 (2CH_2_), 32.13 (CH_2_), 47.70 (2CH_2_), 108.79 (C), 111.74 (CH), 115.13 (2CH), 118.74 (CH), 119.19 (CH), 121.33 (CH), 124.32 (CH), 124.75 (C), 127.90 (C), 128.27 (2CH), 136.45 (C), 146.90 (CH), 152.26 (C), 172.72 (C). HRMS (ESI) (*m*/*z*): [M + H]^+^ calcd. for C_21_H_22_N_4_O: 347.1866, found: 347.1864.

2-(1*H*-Indol-3-yl)-*N*′-(4-morpholinobenzylidene)acetohydrazide (**3b**)

Yield: 85%. M.p.: 306–307 °C. IR ν_max_ (cm^−1^): 3275.13, 3178.69, 3055.24, 2964.59, 2922.16, 2870.08, 2825.72, 1660.71, 1604.77, 1558.48, 1541.12, 1519.91, 1506.41, 1489.05, 1456.26, 1446.61, 1425.40, 1392.61, 1375.25, 1338.60, 1313.52, 1301.95, 1259.52, 1224.80, 1186.22, 1176.58, 1159.22, 1109.07, 1095.57, 1062.78, 1045.42, 1006.84, 958.62, 921.97, 875.68, 858.32, 846.75, 823.60, 798.53, 786.96, 742.59, 682.80. ^1^H NMR (400 MHz, DMSO-*d_6_*): 3.19 (t, *J =* 4.41 Hz, 4.62 Hz, 4H), 3.72–3.75 (m, 4H), 3.61 and 4.02 (2s, 2H), 6.92–7.08 (m, 4H), 7.21 (dd, *J =* 2.4 Hz, 12.8 Hz, 1H), 7.31–7.35 (m, 1H), 7.49–7.60 (m, 3H), 7.88 and 8.07 (2s, 1H), 10.85 and 10.89 (2s, 1H), 11.05 and 11.28 (2s, 1H). ^13^C NMR (100 MHz, DMSO-*d_6_*): 32.13 (CH_2_), 53.79 (2CH_2_), 66.40 (2CH_2_), 108.79 (C), 111.74 (CH), 115.13 (2CH), 118.74 (CH), 119.19 (CH), 121.33 (CH), 124.32 (CH), 124.75 (C), 127.91 (C), 128.27 (2CH), 136.45 (C), 146.90 (CH), 152.29 (C), 172.73 (C). HRMS (ESI) (*m*/*z*): [M + H]^+^ calcd. for C_21_H_22_N_4_O_2_: 363.1816, found: 363.1824.

2-(1*H*-Indol-3-yl)-*N*′-[4-(piperidin-1-yl)benzylidene]acetohydrazide (**3c**)

Yield: 80%. M.p.: 265–266 °C. IR ν_max_ (cm^−1^): 3203.76, 3082.25, 3034.03, 2972.31, 2935.66, 2856.58, 2825.72, 1668.43, 1598.99, 1552.70, 1514.12, 1448.54, 1427.32, 1384.89, 1350.17, 1282.66, 1247.94, 1220.94, 1182.36, 1124.50, 1024.20, 962.48, 914.26, 858.32, 804.32, 721.38, 651.94. ^1^H NMR (400 MHz, DMSO-*d_6_*): 1.58 (brs, 6H), 3.32 (brs, 4H), 3.60 and 4.02 (2s, 2H), 6.92–7.08 (m, 4H), 7.21 (dd, *J =* 2.4 Hz, 12.8 Hz, 1H), 7.31–7.35 (m, 1H), 7.48–7.59 (m, 3H), 7.88 and 8.08 (2s, 1H), 10.85 and 10.89 (2s, 1H), 11.05 and 11.28 (2s, 1H). ^13^C NMR (100 MHz, DMSO-*d_6_*): 24.40 (CH_2_), 25.42 (2CH_2_), 32.12 (CH_2_), 48.95 (2CH_2_), 108.79 (C), 111.74 (CH), 115.13 (2CH), 118.74 (CH), 119.19 (CH), 121.33 (CH), 124.32 (CH), 124.75 (C), 127.91 (C), 128.27 (2CH), 136.45 (C), 146.90 (CH), 152.26 (C), 172.73 (C). HRMS (ESI) (*m*/*z*): [M + H]^+^ calcd. for C_22_H_24_N_4_O: 361.2023, found: 361.2031.

2-(1*H*-Indol-3-yl)-*N*′-[4-(4-methylpiperazin-1-yl)benzylidene]acetohydrazide (**3d**)

Yield: 81%. M.p.: 218–220 °C. IR ν_max_ (cm^−1^): 3398.57, 3205.69, 3165.19, 3111.18, 3043.67, 2939.52, 2883.58, 2831.50, 1649.49, 1602.85, 1517.98, 1446.61, 1427.32, 1409.96, 1377.17, 1340.53, 1286.52, 1232.51, 1184.29, 1159.22, 1141.86, 1124.50, 1105.21, 1080.14, 1001.06, 956.69, 943.19, 921.97, 806.25, 794.67, 742.59, 686.66. ^1^H NMR (400 MHz, DMSO-*d_6_*): 2.21 (s, 3H), 2.40–2.44 (m, 4H), 3.20–3.22 (m, 4H), 3.59 and 4.02 (2s, 2H), 6.92–7.08 (m, 4H), 7.21 (dd, *J =* 2.4 Hz, 12.8 Hz, 1H), 7.31–7.35 (m, 1H), 7.48–7.59 (m, 3H), 7.88 and 8.08 (2s, 1H), 10.85 and 10.89 (2s, 1H), 11.05 and 11.28 (2s, 1H). ^13^C NMR (100 MHz, DMSO-*d_6_*): 32.13 (CH_2_), 46.21 (CH_3_), 47.69 (2CH_2_), 54.89 (2CH_2_), 108.79 (C), 111.74 (CH), 115.13 (2CH), 118.74 (CH), 119.19 (CH), 121.33 (CH), 124.32 (CH), 124.75 (C), 127.91 (C), 128.27 (2CH), 136.45 (C), 146.90 (CH), 152.28 (C), 172.73 (C). HRMS (ESI) (*m*/*z*): [M + H]^+^ calcd. for C_22_H_25_N_5_O: 376.2132, found: 376.2148.

2-(1*H*-Indol-3-yl)-*N*′-(4-methylsulfonylbenzylidene)acetohydrazide (**3e**)

Yield: 86%. M.p.: 264–265 °C. IR ν_max_ (cm^−1^): 3344.57, 3205.69, 3055.24, 2929.87, 2897.08, 1668.43, 1604.77, 1556.55, 1489.05, 1454.33, 1408.04, 1365.60, 1328.95, 1313.52, 1290.38, 1242.16, 1222.87, 1199.72, 1145.72, 1089.78, 1055.06, 1018.41, 983.70, 972.12, 956.69, 943.19, 869.90, 835.18, 792.74, 769.60, 750.31, 729.09, 686.66, 651.94. ^1^H NMR (400 MHz, DMSO-*d_6_*): 3.24 (s, 3H), 3.67 and 4.09 (2s, 2H), 6.94–7.09 (m, 2H), 7.25 (dd, *J =* 2.4 Hz, 9.6 Hz, 1H), 7.34 (t, *J =* 8.0 Hz, 8.4 Hz, 1H), 7.56–7.60 (m, 1H), 7.90–7.96 (m, 4H), 8.07 and 8.31 (2s, 1H), 10.87 and 10.93 (2s, 1H), 11.52 and 11.76 (2s, 1H). ^13^C NMR (100 MHz, DMSO-*d_6_*): 31.87 (CH_2_), 43.64 (CH_3_), 108.10 (C), 111.51 (CH), 118.53 (CH), 118.87 (CH), 121.12 (CH), 124.18 (CH), 127.31 (C), 127.50 (2CH), 127.70 (2CH), 136.19 (C), 139.35 (C), 141.21 (C), 144.37 (CH), 173.17 (C). HRMS (ESI) (*m*/*z*): [M + H]^+^ calcd. for C_18_H_17_N_3_O_3_S: 356.1063, found: 356.1071.

2-(1*H*-Indol-3-yl)-*N*′-[1-(4-methylsulfonylphenyl)ethylidene]acetohydrazide (**3f**)

Yield: 81%. M.p.: 204–205 °C. IR ν_max_ (cm^−1^): 3342.64, 3190.26, 3088.03, 3032.10, 3005.10, 2924.09, 2848.86, 1668.43, 1585.49, 1562.34, 1489.05, 1456.26, 1417.68, 1394.53, 1338.60, 1296.16, 1280.73, 1226.73, 1188.15, 1145.72, 1093.64, 1070.49, 1008.77, 977.91, 964.41, 852.54, 839.03, 788.89, 758.02, 740.67, 717.52, 700.16, 688.59. ^1^H NMR (400 MHz, DMSO-*d_6_*): 2.30 (s, 3H), 3.26 (s, 3H), 3.81 and 4.12 (2s, 2H), 6.95–7.07 (m, 2H), 7.20–7.36 (m, 2H), 7.54–7.61 (m, 1H), 8.01 (d, *J =* 8.0 Hz, 2H), 8.15 (d, *J =* 8.8 Hz, 2H), 10.63 and 10.66 (2s, 1H), 10.84 and 10.89 (2s, 1H). ^13^C NMR (100 MHz, DMSO-*d_6_*): 14.96 (CH_3_), 31.87 (CH_2_), 43.42 (CH_3_), 108.10 (C), 111.26 (CH), 118.25 (CH), 118.64 (CH), 121.12 (CH), 123.84 (CH), 126.70 (C), 127.11 (2CH), 127.39 (2CH), 141.55 (C), 142.06 (C), 142.97 (C), 156.20 (C), 173.17 (C). HRMS (ESI) (*m*/*z*): [M + H]^+^ calcd. for C_19_H_19_N_3_O_3_S: 370.1220, found: 370.1202.

2-(1*H*-Indol-3-yl)-*N*′-[1-(4-morpholinophenyl)ethylidene]acetohydrazide (**3g**)

Yield: 79%. M.p.: 198–199 °C. IR ν_max_ (cm^−1^): 3269.34, 3080.32, 3047.53, 2966.52, 2916.37, 2848.86, 1668.43, 1608.63, 1593.20, 1546.91, 1516.05, 1454.33, 1444.68, 1417.68, 1379.10, 1361.74, 1340.53, 1301.95, 1263.37, 1236.37, 1197.79, 1118.71, 1068.56, 1051.20, 1026.13, 937.40, 923.90, 864.11, 821.68, 798.53, 742.59, 729.09, 648.08. ^1^H NMR (400 MHz, DMSO-*d_6_*): 2.31 (s, 3H), 3.19 (t, *J =* 4.41 Hz, 4.62 Hz, 4H), 3.72–3.75 (m, 4H), 3.60 and 4.02 (2s, 2H), 6.92–7.08 (m, 4H), 7.21 (dd, *J =* 2.4 Hz, 12.8 Hz, 1H), 7.31–7.35 (m, 1H), 7.48–7.59 (m, 3H), 10.85 and 10.89 (2s, 1H), 11.05 and 11.27 (2s, 1H). ^13^C NMR (100 MHz, DMSO-*d_6_*): 14.96 (CH_3_), 32.13 (CH_2_), 53.79 (2CH_2_), 66.40 (2CH_2_), 108.79 (C), 111.74 (CH), 115.13 (2CH), 118.74 (CH), 119.19 (CH), 121.33 (CH), 124.32 (CH), 124.75 (C), 127.91 (C), 128.27 (2CH), 136.45 (C), 143.30 (C), 156.18 (C), 172.73 (C). HRMS (ESI) (*m*/*z*): [M + H]^+^ calcd. for C_22_H_24_N_4_O_2_: 377.1972, found: 377.1982.

2-(1*H*-Indol-3-yl)-*N*′-[4-(2-morpholinoethoxy)benzylidene]acetohydrazide (**3h**)

Yield: 83%. M.p.: 132–135 °C. IR ν_max_ (cm^−1^): 3383.14, 3319.49, 3196.05, 3045.60, 2958.80, 2918.30, 2850.79, 1664.57, 1604.77, 1548.84, 1510.26, 1456.26, 1421.54, 1355.96, 1340.53, 1303.88, 1240.23, 1201.65, 1170.79, 1116.78, 1049.28, 1010.70, 983.70, 952.84, 925.83, 860.25, 831.32, 742.59, 646.15. ^1^H NMR (400 MHz, DMSO-*d_6_*): 2.45–2.47 (m, 4H), 2.66–2.70 (m, 2H), 3.55–3.58 (m, 4H), 4.04 (s, 2H), 4.08–4.12 (m, 2H), 6.95–7.07 (m, 4H), 7.23 (dd, *J =* 2.4 Hz, 12.4 Hz, 1H), 7.34 (t, *J =* 8.0 Hz, 1H), 7.57–7.65 (m, 3H), 7.94 and 8.16 (2s, 1H), 10.86 and 10.91 (2s, 1H), 11.15 and 11.39 (2s, 1H). ^13^C NMR (100 MHz, DMSO-*d_6_*): 31.85 (CH_2_), 53.79 (2CH_2_), 57.12 (CH_2_), 65.64 (CH_2_), 66.35 (2CH_2_), 108.44 (C), 111.48 (CH), 115.05 (2CH), 118.47 (CH), 118.91 (CH), 121.07 (CH), 124.07 (CH), 127.18 (C), 127.63 (C), 128.41 (2CH), 136.19 (C), 146.18 (CH), 159.90 (C), 172.64 (C). HRMS (ESI) (*m*/*z*): [M + H]^+^ calcd. for C_23_H_26_N_4_O_3_: 407.2078, found: 407.2071.

2-(1*H*-Indol-3-yl)-*N*′-[(1-methyl-1*H*-indol-3-yl)methylene]acetohydrazide (**3i**)

Yield: 84%. M.p.: 221–224 °C. IR ν_max_ (cm^−1^): 3414.00, 3147.83, 3101.54, 3061.03, 2980.02, 2945.30, 2908.65, 2819.93, 1651.07, 1612.49, 1570.06, 1539.20, 1502.55, 1462.04, 1452.40, 1421.54, 1404.18, 1377.17, 1346.31, 1332.81, 1321.24, 1253.73, 1244.09, 1197.79, 1157.29, 1139.93, 1120.64, 1087.85, 1072.42, 1045.42, 1008.77, 948.98, 933.55, 900.76, 856.39, 808.17, 785.03, 744.52, 734.88, 673.16. ^1^H NMR (400 MHz, DMSO-*d_6_*): 3.64 and 4.13 (2s, 2H), 3.79 (s, 3H), 6.94–7.18 (m, 3H), 7.22–7.28 (m, 2H), 7.36 (t, *J =* 8.4 Hz, 8.8 Hz, 1H), 7.47 (t, *J =* 8.4 Hz, 9.2 Hz, 1H), 7.64 (t, *J =* 7.6 Hz, 1H), 7.73 (d, *J =* 2.4 Hz, 1H), 8.20–8.38 (m, 2H), 10.86 and 10.92 (2s, 1H), 10.99 and 11.19 (2s, 1H). ^13^C NMR (100 MHz, DMSO-*d_6_*): 31.94 (CH_2_), 32.90 (CH_3_), 108.74 (C), 110.42 (CH), 110.83 (CH), 111.49 (C), 118.50 (CH), 118.93 (CH), 120.89 (CH), 121.10 (CH), 121.86 (CH), 122.80 (CH), 123.96 (CH), 124.70 (C), 127.70 (C), 133.81 (CH), 136.23 (C), 137.76 (C), 142.98 (CH), 172.09 (C). HRMS (ESI) (*m*/*z*): [M + H]^+^ calcd. for C_20_H_18_N_4_O: 331.1553, found: 331.1538.

2-(1*H*-Indol-3-yl)-*N*′-[(5-methoxy-1*H*-indol-3-yl)methylene]acetohydrazide (**3j**)

Yield: 82%. M.p.: 231–233 °C. IR ν_max_ (cm^−1^): 3415.93, 3373.50, 3049.46, 3012.81, 2958.80, 2931.80, 2877.79, 2829.57, 1654.92, 1614.42, 1577.77, 1539.20, 1487.12, 1456.26, 1421.54, 1396.46, 1354.03, 1342.46, 1307.74, 1292.31, 1261.45, 1213.23, 1182.36, 1176.58, 1130.29, 1105.21, 1087.85, 1072.42, 1049.28, 1022.27, 1006.84, 950.91, 923.90, 856.39, 810.10, 744.52, 725.23, 671.23, 651.94. ^1^H NMR (400 MHz, DMSO-*d_6_*): 3.59 (s, 3H), 3.74 and 4.16 (2s, 2H), 6.83 (dd, *J =* 2.4 Hz, 8.8 Hz, 1H), 6.93–7.10 (m, 2H), 7.27–7.38 (m, 3H), 7.64 (t, *J =* 8.8 Hz, 9.2 Hz, 1H), 7.72–7.80 (m, 2H), 8.23 and 8.41 (2s, 1H), 10.85 and 10.91 (2s, 1H), 11.02 and 11.18 (2s, 1H), 11.40 (s, 1H). ^13^C NMR (100 MHz, DMSO-*d_6_*): 31.90 (CH_2_), 55.01 (CH_3_), 103.47 (CH), 108.71 (C), 111.50 (C), 111.59 (CH), 112.39 (CH), 112.64 (CH), 118.52 (CH), 118.83 (CH), 121.14 (CH), 123.88 (CH), 124.83 (C), 127.73 (C), 130.56 (C), 132.19 (CH), 136.19 (C), 143.76 (CH), 154.55 (C), 172.04 (C). HRMS (ESI) (*m*/*z*): [M + H]^+^ calcd. for C_20_H_18_N_4_O_2_: 347.1503, found: 347.1505.

#### 4.1.4. General Method for the Preparation of 4-aryl-1-[2-(1*H*-indol-3-yl)acetyl]thiosemicarbazide Derivatives (**4a-g**)

A mixture of compound **2** and aryl isothiocyanate in ethanol was stirred at room temperature for 8 h. The precipitate was filtered off. The product was crystallized from ethanol.

4-(4-Bromophenyl)-1-[2-(1*H*-indol-3-yl)acetyl]thiosemicarbazide (**4a**) 

Yield: 87%. M.p.: 187–189 °C. IR ν_max_ (cm^−1^): 3390.86, 3311.78, 3286.70, 3207.62, 3143.97, 3057.17, 2997.38, 2927.94, 1680.00, 1647.21, 1620.21, 1589.34, 1544.98, 1506.41, 1485.19, 1452.40, 1419.61, 1352.10, 1309.67, 1282.66, 1247.94, 1207.44, 1138.00, 1087.85, 1074.35, 1049.28, 1004.91, 987.55, 871.82, 823.60, 792.74, 736.81, 715.59, 669.30. ^1^H NMR (400 MHz, DMSO-*d_6_*): 3.63 (s, 2H), 6.97 (t, *J =* 6.8 Hz, 1H), 7.07 (t, *J =* 6.8 Hz, 1H), 7.25 (d, *J =* 2.4 Hz, 1H), 7.34 (d, *J =* 8.0 Hz, 1H), 7.42 (d, *J =* 8.0 Hz, 2H), 7.51 (d, *J =* 8.4 Hz, 2H), 7.59 (d, *J =* 7.6 Hz, 1H), 9.59 (brs, 1H), 9.73 (s, 1H), 10.10 (brs, 1H), 10.89 (s, 1H). ^13^C NMR (100 MHz, DMSO-*d_6_*): 31.18 (CH_2_), 108.39 (C), 111.75 (CH), 118.80 (CH), 119.26 (CH), 121.46 (CH), 122.70 (C), 124.44 (CH), 127.70 (C), 129.40 (2CH), 131.37 (2CH), 136.51 (C), 139.05 (C), 170.35 (C), 181.10 (C). HRMS (ESI) (*m*/*z*): [M + H]^+^ calcd. for C_17_H_15_BrN_4_OS: 403.0223, found: 403.0204.

4-(4-Trifluoromethylphenyl)-1-[2-(1*H*-indol-3-yl)acetyl]thiosemicarbazide (**4b**) 

Yield: 80%. M.p.: 184–186 °C. IR ν_max_ (cm^−1^): 3392.79, 3315.63, 3292.49, 3223.05, 3163.26, 3070.68, 2995.45, 2927.94, 1681.93, 1649.14, 1616.35, 1568.13, 1544.98, 1504.48, 1454.33, 1419.61, 1357.89, 1321.24, 1246.02, 1224.80, 1209.37, 1184.29, 1163.08, 1132.21, 1120.64, 1112.93, 1085.92, 1070.49, 1012.63, 985.62, 846.75, 788.89, 736.81, 711.73, 665.44. ^1^H NMR (400 MHz, DMSO-*d_6_*): 3.64 (s, 2H), 6.97 (t, *J =* 6.8 Hz, 1H), 7.07 (t, *J =* 6.8 Hz, 1H), 7.26 (d, *J =* 2.0 Hz, 1H), 7.34 (d, *J =* 8.0 Hz, 1H), 7.60 (d, *J =* 7.6 Hz, 1H), 7.67–7.75 (m, 4H), 9.75 (brs, 1H), 9.88 (s, 1H), 10.14 (brs, 1H), 10.89 (s, 1H). ^13^C NMR (100 MHz, DMSO-*d_6_*): 31.16 (CH_2_), 108.36 (C), 111.76 (CH), 118.80 (CH), 119.25 (CH), 121.45 (CH), 123.45 (CH), 124.46 (2CH), 125.64 (C), 126.15 (C), 127.69 (2CH), 132.50 (C), 136.51 (C), 143.45 (C), 170.35 (C), 181.10 (C). HRMS (ESI) (*m*/*z*): [M + H]^+^ calcd. for C_18_H_15_F_3_N_4_OS: 393.0991, found: 393.0989.

4-(4-Cyanophenyl)-1-[2-(1*H*-indol-3-yl)acetyl]thiosemicarbazide (**4c**) 

Yield: 89%. M.p.: 180–182 °C. IR ν_max_ (cm^−1^): 3425.58, 3313.71, 3284.77, 3201.83, 3145.90, 3059.10, 2995.45, 2956.87, 2914.44, 2223.92, 1680.00, 1651.07, 1620.21, 1602.85, 1541.12, 1510.26, 1475.54, 1454.33, 1409.96, 1334.74, 1290.38, 1244.09, 1226.73, 1203.58, 1174.65, 1136.07, 1093.64, 1060.85, 1012.63, 975.98, 837.11, 790.81, 769.60, 734.88, 692.44. ^1^H NMR (400 MHz, DMSO-*d_6_*): 3.64 (s, 2H), 6.97 (t, *J =* 7.2 Hz, 7.6 Hz, 1H), 7.07 (t, *J =* 7.2 Hz, 7.6 Hz, 1H), 7.26 (s, 1H), 7.35 (d, *J =* 7.6 Hz, 1H), 7.60 (d, *J =* 7.2 Hz, 1H), 7.78 (s, 4H), 9.76 (brs, 1H), 9.97 (s, 1H), 10.16 (brs, 1H), 10.90 (s, 1H). ^13^C NMR (100 MHz, DMSO-*d_6_*): 31.17 (CH_2_), 108.04 (C), 109.58 (C), 111.49 (CH), 118.53 (C), 118.97 (CH), 119.16 (CH), 121.20 (CH), 124.20 (CH), 127.40 (C), 129.32 (2CH), 132.55 (2CH), 136.24 (C), 143.87 (C), 170.35 (C), 181.10 (C). HRMS (ESI) (*m*/*z*): [M + H]^+^ calcd. for C_18_H_15_N_5_OS: 350.1070, found: 350.1063.

4-[4-(Piperidin-1-ylsulfonyl)phenyl]-1-[2-(1*H*-indol-3-yl)acetyl]thiosemicarbazide (**4d**) 

Yield: 85%. M.p.: 182–184 °C. IR ν_max_ (cm^−1^): 3390.86, 3288.63, 3197.98, 3089.96, 2939.52, 2850.79, 1645.28, 1595.13, 1550.77, 1496.76, 1467.83, 1404.18, 1336.67, 1315.45, 1276.88, 1244.09, 1226.73, 1215.15, 1149.57, 1093.64, 1053.13, 1028.06, 1012.63, 983.70, 929.69, 860.25, 839.03, 819.75, 777.31, 752.24, 738.74, 719.45, 698.23, 667.37. ^1^H NMR (400 MHz, DMSO-*d_6_*): 1.33–1.36 (m, 2H), 1.50–1.54 (m, 4H), 2.87 (t, *J =* 4.8 Hz, 5.2 Hz, 4H), 3.66 (s, 2H), 6.98 (t, *J =* 7.2 Hz, 1H), 7.08 (t, *J =* 7.2 Hz, 1H), 7.27 (d, *J =* 2.0 Hz, 1H), 7.36 (d, *J =* 7.6 Hz, 1H), 7.61 (d, *J =* 8.0 Hz, 1H), 7.67 (d, *J =* 8.4 Hz, 2H), 7.83 (d, *J =* 8.4 Hz, 2H), 9.75 (brs, 1H), 9.93 (s, 1H), 10.16 (brs, 1H), 10.90 (s, 1H). ^13^C NMR (100 MHz, DMSO-*d_6_*): 23.37 (CH_2_), 25.14 (2CH_2_), 31.18 (CH_2_), 47.08 (2CH_2_), 108.35 (C), 111.78 (CH), 118.83 (CH), 119.26 (CH), 121.48 (CH), 124.49 (CH), 125.05 (2CH), 127.68 (C), 128.11 (2CH), 135.47 (C), 136.53 (C), 143.89 (C), 170.34 (C), 181.11 (C). HRMS (ESI) (*m*/*z*): [M + H]^+^ calcd. for C_22_H_25_N_5_O_3_S_2_: 472.1472, found: 472.1452.

4-[4-(1*H*-Pyrazol-1-yl)phenyl]-1-[2-(1*H*-indol-3-yl)acetyl]thiosemicarbazide (**4e**) 

Yield: 85%. M.p.: 196–198 °C. IR ν_max_ (cm^−1^): 3305.99, 3223.05, 3167.12, 3134.33, 3095.75, 3061.03, 2999.31, 2933.73, 1680.00, 1647.21, 1622.13, 1573.91, 1546.91, 1523.76, 1454.33, 1421.54, 1396.46, 1359.82, 1332.81, 1317.38, 1305.81, 1249.87, 1222.87, 1199.72, 1159.22, 1136.07, 1124.50, 1089.78, 1043.49, 1033.85, 1008.77, 985.62, 935.48, 840.96, 792.74, 758.02, 744.52, 717.52, 667.37. ^1^H NMR (400 MHz, DMSO-*d_6_*): 3.65 (s, 2H), 6.53 (t, *J =* 2.4 Hz, 1H), 6.99 (t, *J =* 7.2 Hz, 1H), 7.08 (t, *J =* 7.2 Hz, 1H), 7.27 (d, *J =* 2.0 Hz, 1H), 7.36 (d, *J =* 8.0 Hz, 1H), 7.55 (d, *J =* 8.0 Hz, 1H), 7.62 (d, *J =* 8.0 Hz, 2H), 7.74 (d, *J =* 1.6 Hz, 1H), 7.80 (d, *J =* 8.8 Hz, 2H), 8.46 (d, *J =* 2.4 Hz, 1H), 9.65 (brs, 1H), 9.71 (s, 1H), 10.13 (s, 1H), 10.90 (s, 1H). ^13^C NMR (100 MHz, DMSO-*d_6_*): 31.20 (CH_2_), 108.23 (C), 108.45 (CH), 111.77 (CH), 118.56 (CH), 118.82 (CH), 119.28 (2CH), 121.48 (CH), 124.47 (CH), 126.80 (CH), 127.73 (C), 128.09 (2CH), 136.53 (C), 137.64 (2C), 141.30 (CH), 170.32 (C), 181.10 (C). HRMS (ESI) (*m*/*z*): [M + H]^+^ calcd. for C_20_H_18_N_6_OS: 391.1336, found: 391.1334.

4-(1,3-Benzodioxol-5-yl)-1-[2-(1*H*-indol-3-yl)acetyl]thiosemicarbazide (**4f**) 

Yield: 81%. M.p.: 168–170 °C. IR ν_max_ (cm^−1^): 3300.20, 3209.55, 3149.76, 3057.17, 2929.87, 2897.08, 1678.07, 1643.35, 1591.27, 1539.20, 1500.62, 1481.33, 1454.33, 1419.61, 1334.74, 1282.66, 1240.23, 1197.79, 1122.57, 1089.78, 1037.70, 981.77, 923.90, 850.61, 815.89, 808.17, 790.81, 731.02, 698.23. ^1^H NMR (400 MHz, DMSO-*d_6_*): 3.62 (s, 2H), 6.02 (s, 2H), 6.72 (d, *J =* 8.4 Hz, 1H), 6.87 (d, *J =* 8.0 Hz, 1H), 6.96–7.09 (m, 3H), 7.26 (s, 1H), 7.34 (d, *J =* 8.4 Hz, 1H), 7.60 (d, *J =* 8.0 Hz, 1H), 9.47 (brs, 1H), 9.55 (s, 1H), 10.06 (s, 1H), 10.89 (s, 1H). ^13^C NMR (100 MHz, DMSO-*d_6_*): 30.65 (CH_2_), 101.21 (CH_2_), 107.38 (C), 107.97 (CH), 111.25 (CH), 118.29 (2CH), 118.78 (CH), 120.95 (CH), 123.95 (CH), 127.24 (CH), 133.08 (C), 136.02 (2C), 144.59 (C), 146.56 (C), 170.35 (C), 181.10 (C). HRMS (ESI) (*m*/*z*): [M + H]^+^ calcd. for C_18_H_16_N_4_O_3_S: 369.1016, found: 369.0998.

4-[4-(Benzyloxy)phenyl]-1-[2-(1*H*-indol-3-yl)acetyl]thiosemicarbazide (**4g**) 

Yield: 88%. M.p.: 198–200 °C. IR ν_max_ (cm^−1^): 3394.72, 3290.56, 3213.41, 3155.54, 3059.10, 3032.10, 2939.52, 2873.94, 1681.93, 1649.14, 1618.28, 1564.27, 1546.91, 1504.48, 1456.26, 1417.68, 1381.03, 1359.82, 1294.24, 1244.09, 1219.01, 1170.79, 1138.00, 1089.78, 1051.20, 999.13, 912.33, 879.54, 829.39, 790.81, 734.88, 702.09, 646.15. ^1^H NMR (400 MHz, DMSO-*d_6_*): 3.64 (s, 2H), 5.10 (s, 2H), 6.97–7.01 (m, 3H), 7.06–7.10 (m, 1H), 7.27–7.29 (m, 3H), 7.33–7.47 (m, 6H), 7.61 (d, *J =* 8.0 Hz, 1H), 9.47 (brs, 1H), 9.53 (s, 1H), 10.07 (s, 1H), 10.90 (s, 1H). ^13^C NMR (100 MHz, DMSO-*d_6_*): 30.68 (CH_2_), 69.34 (CH_2_), 107.99 (C), 111.27 (CH), 114.22 (2CH), 118.31 (2CH), 118.79 (CH), 120.96 (CH), 123.95 (CH), 127.25 (C), 127.67 (2CH), 127.80 (2CH), 128.41 (2CH), 132.15 (C), 136.03 (C), 137.08 (C), 155.77 (C), 170.39 (C), 181.17 (C). HRMS (ESI) (*m*/*z*): [M + H]^+^ calcd. for C_24_H_22_N_4_O_2_S: 431.1536, found: 431.1554.

### 4.2. Biochemistry

#### 4.2.1. In Vitro COX Inhibition Assay 

COX (ovine) Colorimetric Inhibitor Screening Assay (Cayman, Ann Arbor, MI, USA) was conducted to detect the peroxidase component of COX-1 and COX-2 according to the manufacturer’s instructions [53]. The assay was performed in triplicate. Half maximal inhibitory concentration (IC_50_) data (µM) were expressed as mean ± SD.

#### 4.2.2. Cell Culture and Drug Treatment

A549 human lung adenocarcinoma and L929 mouse fibroblast cell lines were obtained from American Type Culture Collection (ATCC) (Manassas, VA, USA). Both cell lines were cultured, and drug treatments were carried out as previously reported [31,86].

#### 4.2.3. MTT Assay 

MTT assay was conducted as previously explained in the literature [87] with small modifications [86]. Cisplatin was used as a positive control. The assay was performed in triplicate. IC_50_ data (µM) were expressed as mean ± SD.

#### 4.2.4. Flow Cytometry-Based Apoptosis Detection

FITC Annexin V Apoptosis Detection kit (BD Pharmingen, San Jose, CA, USA) was applied based on the manufacturer’s instructions after the incubation of A549 cells with compound **4a** (at its IC_50_/4 and IC_50_/2 concentrations), compound **3b**, and cisplatin (at their IC_50_/2 and IC_50_ concentrations) for 24 h [87].

#### 4.2.5. Determination of Akt Inhibition

After A549 cells were incubated with compounds **3b** (22.42 μM, 44.84 μM, 89.67 μM), **4a** (22.42 μM, 44.84 μM, 89.67 μM), Akt inhibitor GSK690693 (3.61 μM, 7.23 μM, 14.45 μM), and cisplatin (5.67 μM, 11.34 μM, 22.67 μM) for 24 h, Akt Colorimetric In-Cell ELISA Kit (Thermo Fisher Scientific, Waltham, MA, USA) was used according to the manufacturer’s instructions [87]. The assay was performed in triplicate. IC_50_ data (µM) were expressed as mean ± SD.

#### 4.2.6. Experimental Animals 

Male albino Sprague Dawley rats (~250–300 g) were procured from the Medical and Surgical Experimental Animals Application and Research Center of Eskisehir Osmangazi University (ESOGU). In the animal house, the rats were housed in stainless steel cages under standard atmospheric conditions at 22 ± 1 °C and exposed to 12 h/12 h light/dark cycle [53]. Food and water were given ad libitum. All experiments and protocols reported in this work were approved by ESOGU Animal Experiments Local Ethics Committee (10 December 2018/700).

#### 4.2.7. Chemicals and Drug Administrations

Compounds **3b**, **4a**, and indomethacin (Sigma-Aldrich, St. Louis, MO, USA) were dissolved in 5% dimethyl sulfoxide (DMSO) and then diluted. The final DMSO concentration in the solution was 0.5% (*v*/*v*). The agents were administered by gastric intubation. LPS (Sigma-Aldrich, St. Louis, MO, USA) (1 mg/kg) dissolved in 0.9% sodium chloride solution was intraperitoneally injected only once on the 7th day for the experimentally induced sepsis model [53].

#### 4.2.8. In vivo Experimental Design 

Rats were randomly divided into five groups (*n* = 8) as control group, LPS group, test groups (**3b** and **4a**), and reference group. 0.5% DMSO was used as control solution for LPS group. Indomethacin (5 mg/kg) was used as a reference agent. Control group (Group I) was fed with basal rat chow throughout the experimental period. LPS group (Group II) was fed with basal rat chow for six days (only 0.5% DMSO was administered by gastric intubation) and LPS was injected intraperitoneally in 0.9% sodium chloride solution only once on the 7th day. Groups III, IV, and V were fed with basal rat chow and compound **3b** (10 mg/kg/day), compound **4a** (10 mg/kg/day), and indomethacin were administered, respectively, by gastric intubation for six days. Then, LPS was injected intraperitoneally in 0.9% sodium chloride solution only once on the 7th day for three groups as well. After 24 h of LPS injection, all rats were sacrificed by ketamine (80 mg/kg) ve xylazine (10 mg/kg) anesthesia via intraperitoneal route. Blood samples were collected via cardiac puncture in tubes containing gel for obtaining serum [53].

Serum ALT and AST levels were determined using enzyme-based Roche Diagnostics kit in Roche Modular Systems analyzer by photometric assay [53] based on the manufacturer’s instructions. The other serum samples were stored at −80 °C (Thermo Electron, Waltham, MA, USA) for subsequent analyses of MPO and NO levels.

#### 4.2.9. Determination of MPO Levels

Suzuki’s assay [88] was performed with slight modifications [53]. The rate of MPO-catalyzed oxidation of 3,3′,5,5′-tetramethylbenzidine (TMB) was followed by recording the absorbance increase at 655 nm for 5 min. Taking into account the linear phase of the reaction, the absorbance change was measured per minute. The enzyme activity was expressed as the amount of the enzyme producing one absorbance change per minute under assay conditions [53].

#### 4.2.10. Determination of NO Levels

Nitrate and nitrite, which represent the best index of the entire NO production, are the stable end products of NO *in vivo*. Nitrate in serum was assayed by a slight modification of the Cd-reduction method as reported by Cortas and Wakid [89].

#### 4.2.11. Statistical Analyses

The data used in statistical analyses were obtained from eight animals for each group and statistically evaluated by means of Statistical Package for the Social Sciences (SPSS) for Windows 17.0. Comparisons were performed by one-way ANOVA (Tukey for post-hoc analyses) test. Differences between groups were considered statistically significant at a level of *p* < 0.05.

## 5. Conclusions

In this paper, two classes of indole-based small molecules (**3a-j**, **4a-g**) were designed and synthesized for the targeted therapy of NSCLC. Based on the data gathered from the COX colorimetric inhibitor screening assay, compounds **3b** and **4a** were found to be the selective COX-1 inhibitors in this series with IC_50_ values of 8.90 and 10.00 µM, respectively. In vitro and in vivo assays were conducted to assess their potential for the targeted therapy of NSCLC. The experimental data demonstrate that compound **3b** exerts selective anticancer activity against A549 cells through apoptosis induction and Akt inhibition. Compound **3b** also caused a substantial drop in the serum MPO and NO levels, pointing out its potential as an anti-inflammatory agent. Moreover, compound **3b** decreased the serum aminotransferase (particularly AST) levels. Taken together, compound **3b** stands out as a lead anti-NSCLC agent endowed with in vivo anti-inflammatory action acting as a dual COX-1 and Akt inhibitor. In the view of this work, a new generation of indole-based small molecules with enhanced antitumor potency could be designed through the molecular modification of compound **3b** for the targeted therapy of NSCLC.

## Figures and Tables

**Table 1:** COX inhibitory profiles of compounds **3a-j** and positive controls.

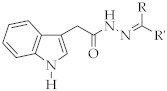					
Compound	R	R′	IC_50_ (µM)		SI *
			COX-1	COX-2	
**3a**	4-(Pyrrolidin-1-yl)phenyl	H	10.35 ± 0.35	12.50 ± 0.71	0.83
**3b**	4-Morpholinophenyl	H	8.90 ± 0.14	71.00 ± 1.41	0.13
**3c**	4-(Piperidin-1-yl)phenyl	H	>100	>100	-
**3d**	4-(4-Methylpiperazin-1-yl)phenyl	H	78.50 ± 8.50	>100	<0.79
**3e**	4-(Methylsulfonyl)phenyl	H	83.75 ± 6.25	35.00 ± 9.90	2.39
**3f**	4-(Methylsulfonyl)phenyl	CH_3_	93.75 ± 6.25	51.00 ± 12.73	1.84
**3g**	4-Morpholinophenyl	CH_3_	51.00 ± 1.00	52.50 ± 0.71	0.97
**3h**	4-(2-Morpholinoethoxy)phenyl	H	38.50 ± 1.5	50.50 ± 0.70	0.76
**3i**	1-Methyl-1*H*-indol-3-yl	H	31.25 ± 1.25	>100	<0.31
**3j**	5-Methoxy-1*H*-indol-3-yl	H	>100	44.50 ± 6.36	>2.25
Indomethacin	-	-	0.12 ± 0.01	0.58 ± 0.08	0.21
Celecoxib	-	-	8.88 ± 0.38	2.75 ± 0.05	3.23

* IC_50_ for COX-1/IC_50_ for COX-2.

**Table 2:** COX inhibitory profiles of compounds **4a-g** and positive controls.

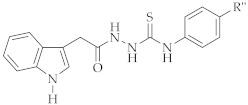				
Compound	R″	IC_50_ (µM)		SI *
		COX-1	COX-2	
**4a**	Br	10.00 ± 0.13	76.50 ± 6.36	0.13
**4b**	CF_3_	>100	61.50 ± 0.71	>1.63
**4c**	CN	>100	56.50 ± 4.49	>1.77
**4d**	Piperidin-1-ylsulfonyl	>100	59.00 ± 8.48	>1.69
**4e**	1*H*-Pyrazol-1-yl	>100	31.50 ± 2.12	>3.17
**4f**	3,4-Methylenedioxy	>100	51.50 ± 0.71	>1.94
**4g**	Benzyloxy	>100	59.50 ± 2.12	>1.68
Indomethacin	-	0.12 ± 0.01	0.58 ± 0.08	0.21
Celecoxib	-	8.88 ± 0.38	2.75 ± 0.05	3.23

* IC_50_ for COX-1/IC_50_ for COX-2.

**Table 3:** IC_50_ values of all compounds for L929 cells.

Compound	IC_50_ (µM)
**3a**	17.33 ± 2.08
**3b**	176.67 ± 5.77
**3c**	21.33 ± 0.58
**3d**	20.00 ± 1.73
**3e**	85.00 ± 17.32
**3f**	<3.90
**3g**	<3.90
**3h**	<3.90
**3i**	42.33 ± 0.58
**3j**	43.00 ± 1.73
**4a**	84.00 ± 19.70
**4b**	22.67 ± 2.08
**4c**	26.33 ± 3.51
**4d**	45.67 ± 6.03
**4e**	62.33 ± 7.51
**4f**	14.00 ± 5.29
**4g**	14.00 ± 3.46

**Table 4:** IC_50_ values of compounds **3b**, **4a,** and cisplatin for A549 cells.

Compound	IC_50_ (µM)
**3b**	89.67 ± 10.78
**4a**	179.33 ± 77.59
Cisplatin	22.67 ± 4.04

**Table 5:** Percentages of typical quadrant analysis of Annexin V FITC/PI flow cytometry of A549 cells treated with compounds **3b**, **4a,** and cisplatin.

Compound	Early Apoptosis (%)	Late Apoptosis (%)	Necrosis (%)	Viability (%)
Control	4.98	1.18	0.36	93.47
Compound **3b** at IC_50_/2	5.89	5.78	0.36	87.97
Compound **3b** at IC_50_	6.84	6.01	0.48	86.66
Compound **4a** at IC_50_/4	7.34	5.19	0.37	87.10
Compound **4a** at IC_50_/2	7.07	6.62	0.60	85.71
Cisplatin at IC_50_/2	3.69	2.88	0.72	92.72
Cisplatin at IC_50_	2.43	2.03	2.34	93.20

A549 cells were cultured for 24 h in medium with compound **4a** (at its IC_50_/4 and IC_50_/2 concentrations), compound **3b,** and cisplatin (at their IC_50_/2 and IC_50_ concentrations). At least 10,000 cells were analyzed per sample, and quadrant analysis was performed.

**Table 6:** Akt inhibitory effects of compounds **3b**, **4a**, GSK690693, and cisplatin in A549 cells.

Compound	IC_50_ (µM)
**3b**	32.50 ± 4.95
**4a**	45.33 ± 6.51
GSK690693	5.93 ± 1.20
Cisplatin	9.30 ± 2.55

**Table 7:** Effects of compounds **3b**, **4a,** and indomethacin on MPO levels.

Groups	MPO (U/L)
Control	1.03 ± 0.51
LPS	1.79 ± 0.27
LPS + Compound **3b**	1.50 ± 0.81
LPS + Compound **4a**	0.84 ± 0.26 ^#^
LPS + Indomethacin	1.06 ± 0.73

Values are given as mean ± standard deviation (SD). Significance according to LPS values, ^#^: *p* < 0.05. One-way ANOVA, post-hoc Tukey test *n* = 8.

**Table 8:** Effects of compounds **3b**, **4a,** and indomethacin on NO levels (µmol/L).

Groups	25% Percentile	Median	75% Percentile
Control	0	0.07	0.14
LPS ***	4.482	6.018	8.386
LPS + Compound **3b**	0.316	0.667	2.772
LPS + Compound **4a** *	1.369	2.509	4.0
LPS + Indomethacin *	0.667	1.281	3.123

Significance relative to control values, *: *p* < 0.05, ***: *p* < 0.001. One-way ANOVA, Kruskal–Wallis test, *n* = 8.

**Table 9:** Effects of compounds **3b**, **4a,** and indomethacin on ALT and AST levels.

Groups	ALT (U/L)	AST (U/L)
Control	45.75 ± 3.81	126.60 ± 24.09
LPS	57.14 ± 22.94	139.90 ± 26.34
LPS + Compound **3b**	50.25 ± 21.86	114.90 ± 22.04
LPS + Compound **4a**	48.29 ± 26.88	132.30 ± 37.06
LPS + Indomethacin	44.00 ± 14.64	123.50 ± 32.14

Values are given as mean ± SD. One-way ANOVA, post-hoc Tukey test, *n* = 8.

## Data Availability

Data are contained within the article or Supplementary Material.

## Supplementary Materials

The following supporting information can be downloaded at: https://www.mdpi.com/article/10.3390/ijms24032648/s1.
