# Supplementary material for: Discovery of Small Molecule COX-1 and Akt Inhibitors as Anti-NSCLC Agents Endowed with Anti-Inflammatory Action
Source: Int J Mol Sci. 2023 Jan 31;24(3):2648. doi: 10.3390/ijms24032648 (PMC9916685; doi:10.3390/ijms24032648)
Supplement: Supplementary file 1 [file ijms-24-02648-s001.zip › ijms-2111155-supplementary.pdf]

# Supplementary Material

# Discovery of small molecule COX-1 and Akt inhibitors as anti-NSCLC agents endowed with anti-inflammatory action

**Mehlika Dilek Altıntop [1], Gülşen Akalın Çiftçi [2,3], Nalan Yılmaz Savaş [3], İpek Ertorun [4], Betül Can [4], Belgin Sever [1], Halide Edip Temel [2], Özkan Alataş [4] and Ahmet Özdemir [1,*]**

[1] Department of Pharmaceutical Chemistry, Faculty of Pharmacy, Anadolu University, 26470 Eskişehir, Turkey; mdaltintop@anadolu.edu.tr (M.D.A.), belginsever@anadolu.edu.tr (B.S.)

[2] Department of Biochemistry, Faculty of Pharmacy, Anadolu University, 26470 Eskişehir, Turkey; gakalin@anadolu.edu.tr (G.A.Ç.), heincedal@anadolu.edu.tr (H.E.T.)

[3] Graduate School of Health Sciences, Anadolu University, 26470 Eskişehir, Turkey; nalan_y@anadolu.edu.tr (N.Y.S.)

[4] Department of Medical Biochemistry, Faculty of Medicine, Eskisehir Osmangazi University, 26480 Eskişehir, Turkey; erdoganipek@gmail.com (İ.E.), bcan@ogu.edu.tr (B.C.), oalatas@ogu.edu.tr (Ö.A.)

* Correspondence: mdaltintop@anadolu.edu.tr (M.D.A.); ahmeto@anadolu.edu.tr (A.Ö.); Tel.: +90-222-335-0580 (ext. 3772) (M.D.A); Tel.: +90-222-335-0580 (ext. 3780) (A.Ö.).

**List of Contents**


| Figure | Page |
|---|---|
| **Figure S1.** IR spectrum of compound **3a** | S5 |
| **Figure S2.** $^{1}H$ NMR spectrum of compound **3a** | S6 |
| **Figure S3.** $^{13}C$ NMR spectrum of compound **3a** | S6 |
| **Figure S4.** HRMS spectrum of compound **3a** | S7 |
| **Figure S5.** IR spectrum of compound **3b** | S8 |
| **Figure S6.** $^{1}H$ NMR spectrum of compound **3b** | S9 |
| **Figure S7.** $^{13}C$ NMR spectrum of compound **3b** | S9 |
| **Figure S8.** HRMS spectrum of compound **3b** | S10 |
| **Figure S9.** IR spectrum of compound **3c** | S11 |
| **Figure S10.** $^{1}H$ NMR spectrum of compound **3c** | S12 |
| **Figure S11.** $^{13}C$ NMR spectrum of compound **3c** | S12 |
| **Figure S12.** HRMS spectrum of compound **3c** | S13 |
| **Figure S13.** IR spectrum of compound **3d** | S14 |
| **Figure S14.** $^{1}H$ NMR spectrum of compound **3d** | S15 |
| **Figure S15.** $^{13}C$ NMR spectrum of compound **3d** | S15 |
| **Figure S16.** HRMS spectrum of compound **3d** | S16 |
| **Figure S17.** IR spectrum of compound **3e** | S17 |
| **Figure S18.** $^{1}H$ NMR spectrum of compound **3e** | S18 |
| **Figure S19.** $^{13}C$ NMR spectrum of compound **3e** | S18 |
| **Figure S20.** HRMS spectrum of compound **3e** | S19 |
| **Figure S21.** IR spectrum of compound **3f** | S20 |
| **Figure S22.** $^{1}H$ NMR spectrum of compound **3f** | S21 |
| **Figure S23.** $^{13}C$ NMR spectrum of compound **3f** | S21 |
| **Figure S24.** HRMS spectrum of compound **3f** | S22 |
| **Figure S25.** IR spectrum of compound **3g** | S23 |
| **Figure S26.** $^{1}H$ NMR spectrum of compound **3g** | S24 |
| **Figure S27.** $^{13}C$ NMR spectrum of compound **3g** | S24 |
| **Figure S28.** HRMS spectrum of compound **3g** | S25 |
| **Figure S29.** IR spectrum of compound **3h** | S26 |
| **Figure S30.** $^{1}H$ NMR spectrum of compound **3h** | S27 |

**Figure S31.** $^{13}$C NMR spectrum of compound **3h** S27

**Figure S32.** HRMS spectrum of compound **3h** S28

**Figure S33.** IR spectrum of compound **3i** S29

**Figure S34.** $^{1}$H NMR spectrum of compound **3i** S30

**Figure S35.** $^{13}$C NMR spectrum of compound **3i** S30

**Figure S36.** HRMS spectrum of compound **3i** S31

**Figure S37.** IR spectrum of compound **3j** S32

**Figure S38.** $^{1}$H NMR spectrum of compound **3j** S33

**Figure S39.** $^{13}$C NMR spectrum of compound **3j** S33

**Figure S40.** HRMS spectrum of compound **3j** S34

**Figure S41.** IR spectrum of compound **4a** S35

**Figure S42.** $^{1}$H NMR spectrum of compound **4a** S36

**Figure S43.** $^{13}$C NMR spectrum of compound **4a** S36

**Figure S44.** HRMS spectrum of compound **4a** S37

**Figure S45.** IR spectrum of compound **4b** S38

**Figure S46.** $^{1}$H NMR spectrum of compound **4b** S39

**Figure S47.** $^{13}$C NMR spectrum of compound **4b** S39

**Figure S48.** HRMS spectrum of compound **4b** S40

**Figure S49.** IR spectrum of compound **4c** S41

**Figure S50.** $^{1}$H NMR spectrum of compound **4c** S42

**Figure S51.** $^{13}$C NMR spectrum of compound **4c** S42

**Figure S52.** HRMS spectrum of compound **4c** S43

**Figure S53.** IR spectrum of compound **4d** S44

**Figure S54.** $^{1}$H NMR spectrum of compound **4d** S45

**Figure S55.** $^{13}$C NMR spectrum of compound **4d** S45

**Figure S56.** HRMS spectrum of compound **4d** S46

**Figure S57.** IR spectrum of compound **4e** S47

**Figure S58.** $^{1}$H NMR spectrum of compound **4e** S48

**Figure S59.** $^{13}$C NMR spectrum of compound **4e** S48

**Figure S60.** HRMS spectrum of compound **4e** S49

**Figure S61.** IR spectrum of compound **4f** S50

**Figure S62.** $^{1}$H NMR spectrum of compound **4f** S51

**Figure S63.** $^{13}$C NMR spectrum of compound **4f** S51

**Figure S64.** HRMS spectrum of compound **4f** S52

**Figure S65.** IR spectrum of compound **4g** S53

**Figure S66.** $^{1}$H NMR spectrum of compound **4g** S54

**Figure S67.** $^{13}$C NMR spectrum of compound **4g** S54

**Figure S68.** HRMS spectrum of compound **4g** S55

**Figure S1.** IR spectrum of compound **3a**

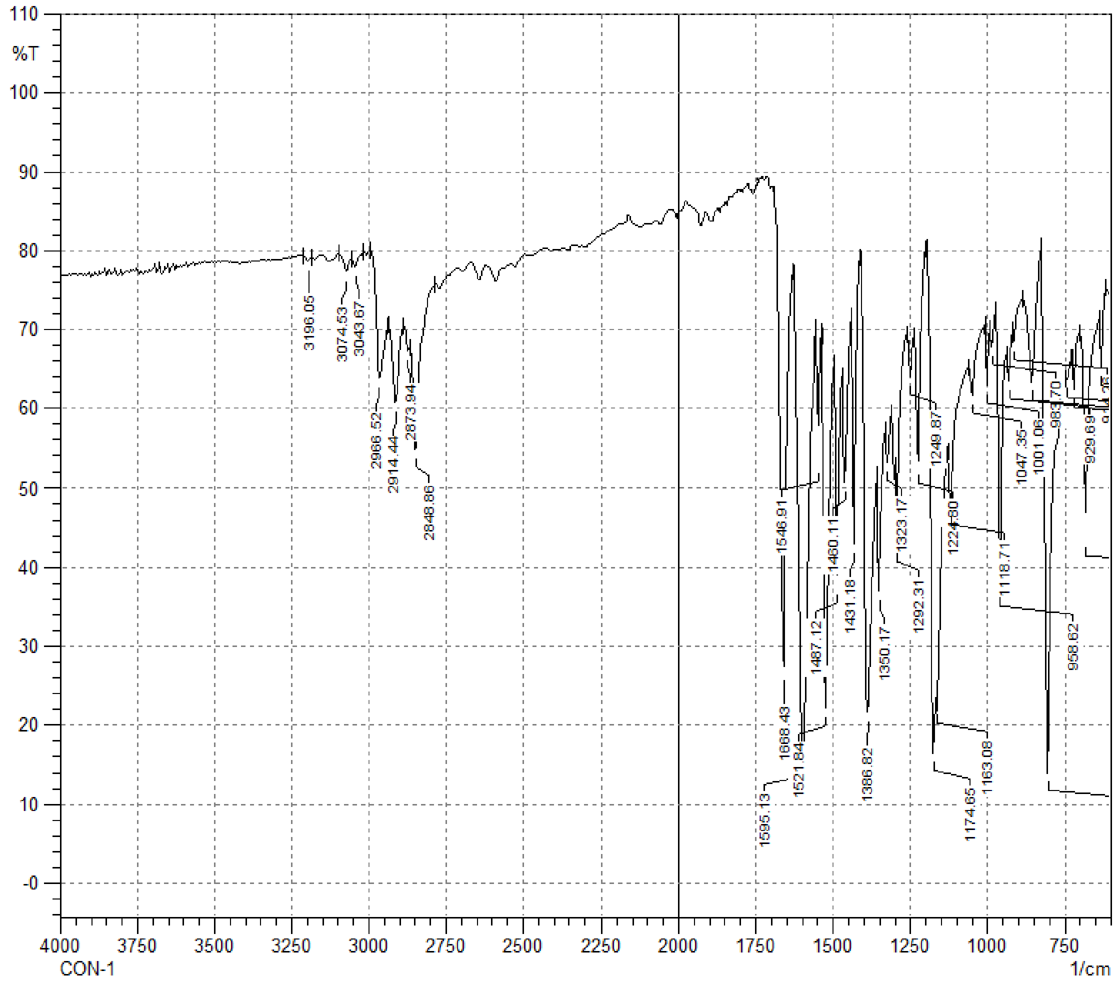

**Figure S2.** $^{1}$H NMR spectrum of compound **3a**

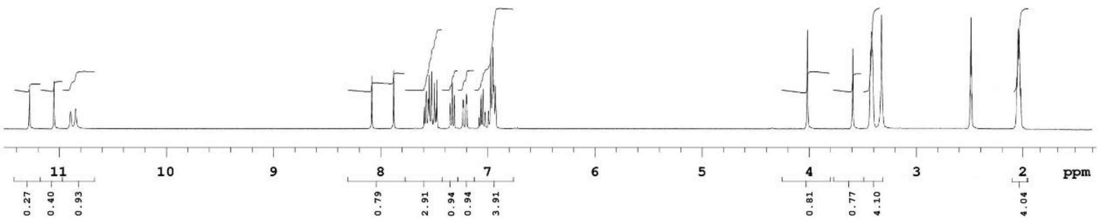


**Figure S3.** $^{13}$C NMR spectrum of compound **3a**

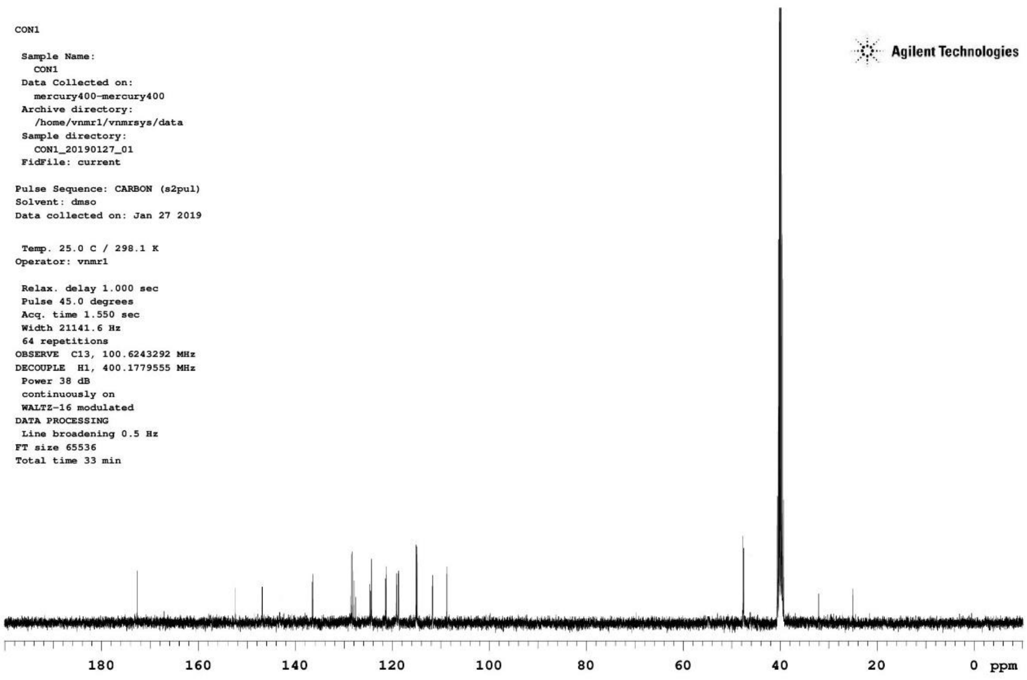

**Figure S4.** HRMS spectrum of compound **3a**

Formula Predictor Report - CON-1_2.lcd

Page 1 of 1

Data File: C:\LabSolutions\Data\Analiz\AOzdemir\CON-1_2.lcd

| Elmt | Val. | Min | Max | Elmt | Val. | Min | Max | Elmt | Val. | Min | Max | Elmt | Val. | Min | Max | Use Adduct |
|---|---|---|---|---|---|---|---|---|---|---|---|---|---|---|---|---|
| H | 1 | 0 | 30 | O | 2 | 1 | 4 | S | 2 | 0 | 2 | Ru | 2 | 0 | 0 | H |
| C | 4 | 15 | 25 | F | 1 | 0 | 0 | Cl | 1 | 0 | 0 | Pd | 2 | 0 | 0 | |
| N | 3 | 1 | 4 | P | 3 | 0 | 0 | Br | 1 | 0 | 0 | I | 3 | 0 | 0 | |

| | | | | | |
|---|---|---|---|---|---|
| Error Margin (ppm): | 10 | DBE Range: | 10.0 - 20.0 | Electron Ions: | both |
| HC Ratio: | unlimited | Apply N Rule: | yes | Use MSn Info: | yes |
| Max Isotopes: | 3 | Isotope RI (%): | 1.00 | Isotope Res: | 9000 |
| MSn Iso RI (%): | 10.00 | MSn Logic Mode: | AND | Max Results: | 500 |

Event#: 1 MS(E+) Ret. Time : 7.173 -> 7.720 Scan# : 1077 -> 1159

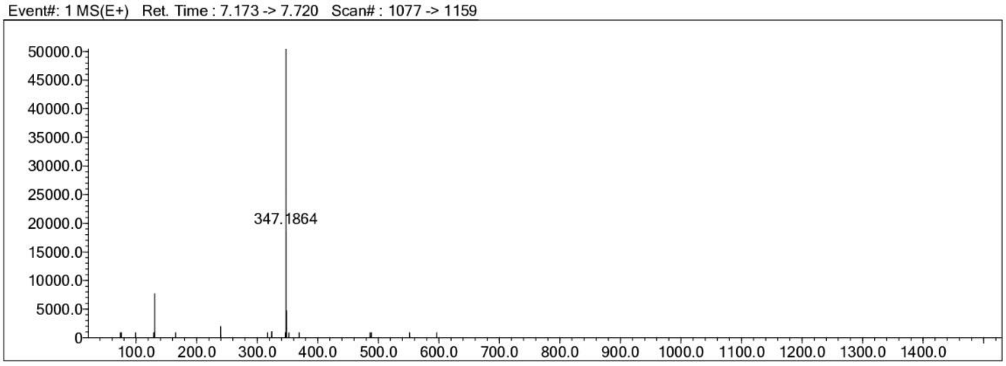


Measured region for 347.1864 m/z

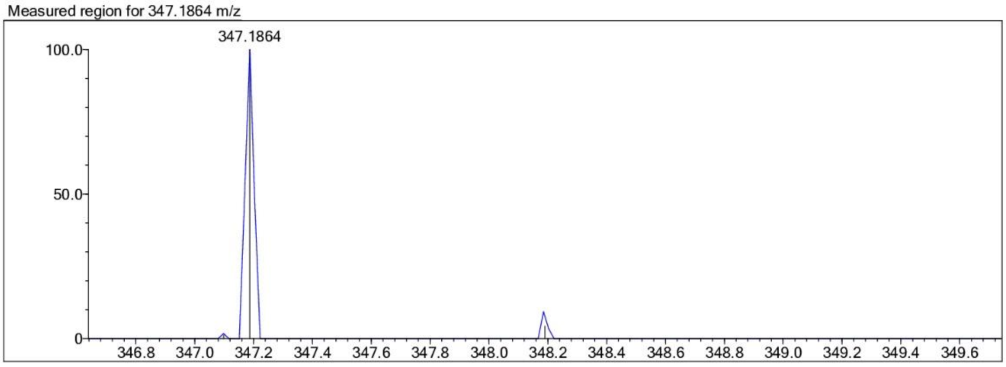


C21 H22 N4 O [M+H]+ : Predicted region for 347.1866 m/z

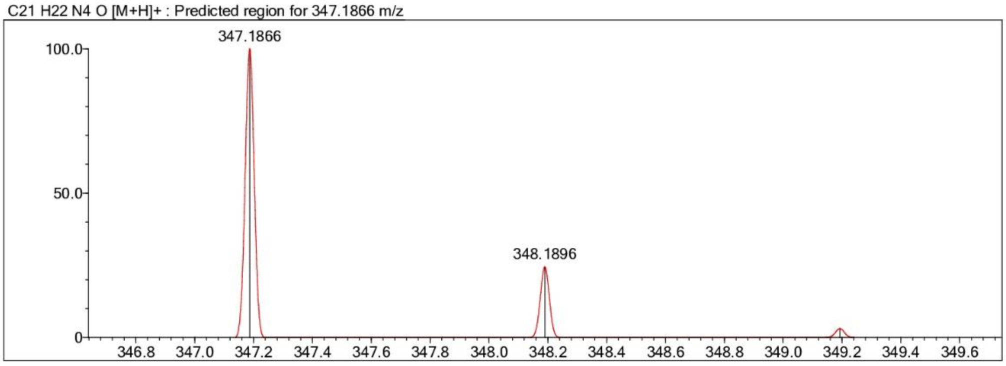


| Rank | Score | Formula (M) | Ion | Meas. m/z | Pred. m/z | Df. (mDa) | Df. (ppm) | Iso | DBE |
|---|---|---|---|---|---|---|---|---|---|
| 1 | 51.62 | C21 H22 N4 O | [M+H]+ | 347.1864 | 347.1866 | -0.2 | -0.58 | 51.62 | 13.0 |

**Figure S5.** IR spectrum of compound **3b**

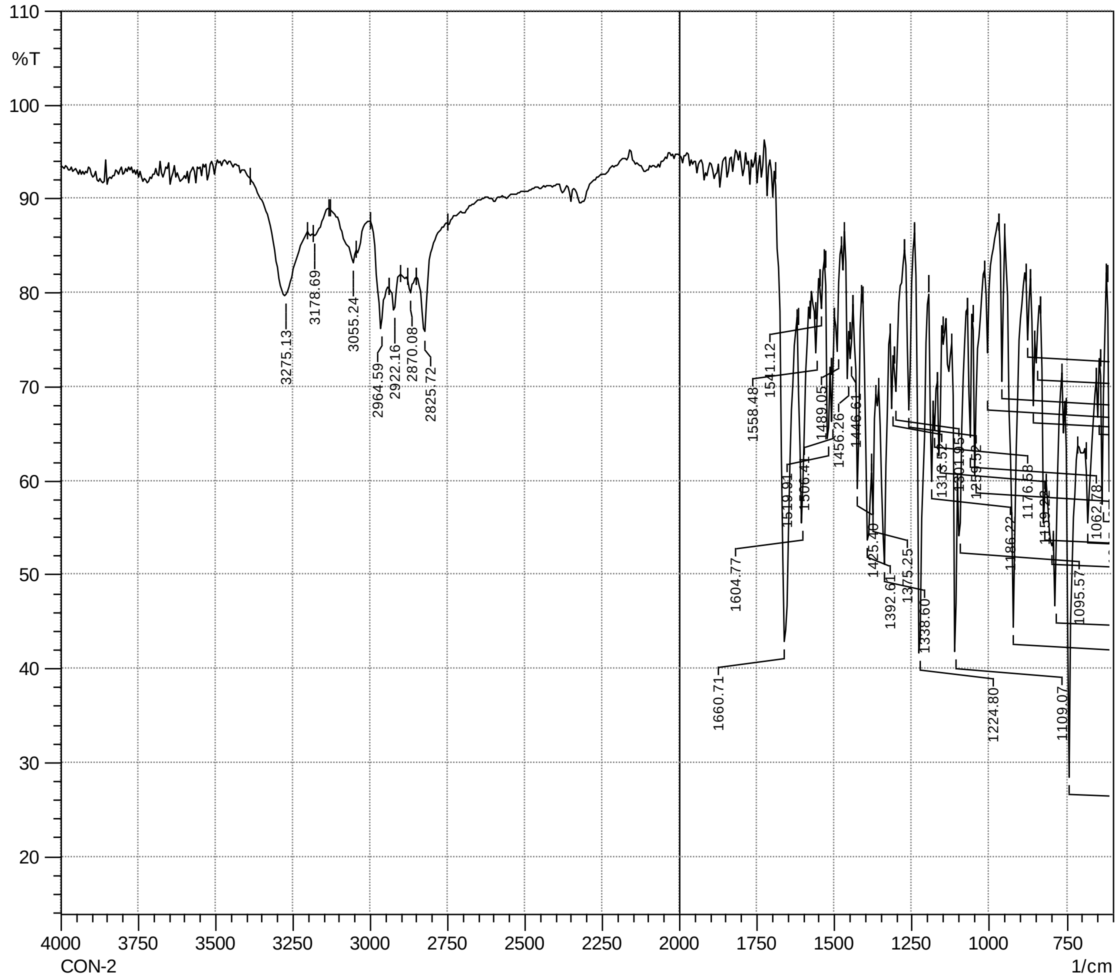

**Figure S6.** $^{1}$H NMR spectrum of compound **3b**

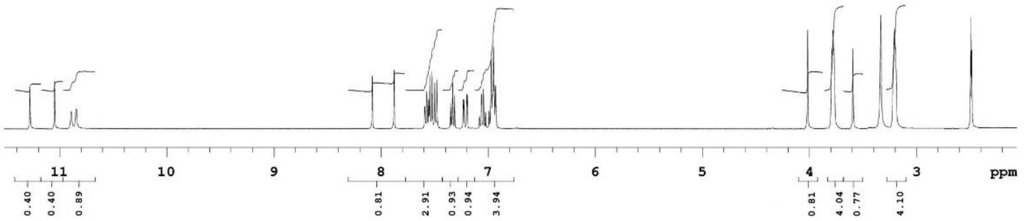


**Figure S7.** $^{13}$C NMR spectrum of compound **3b**

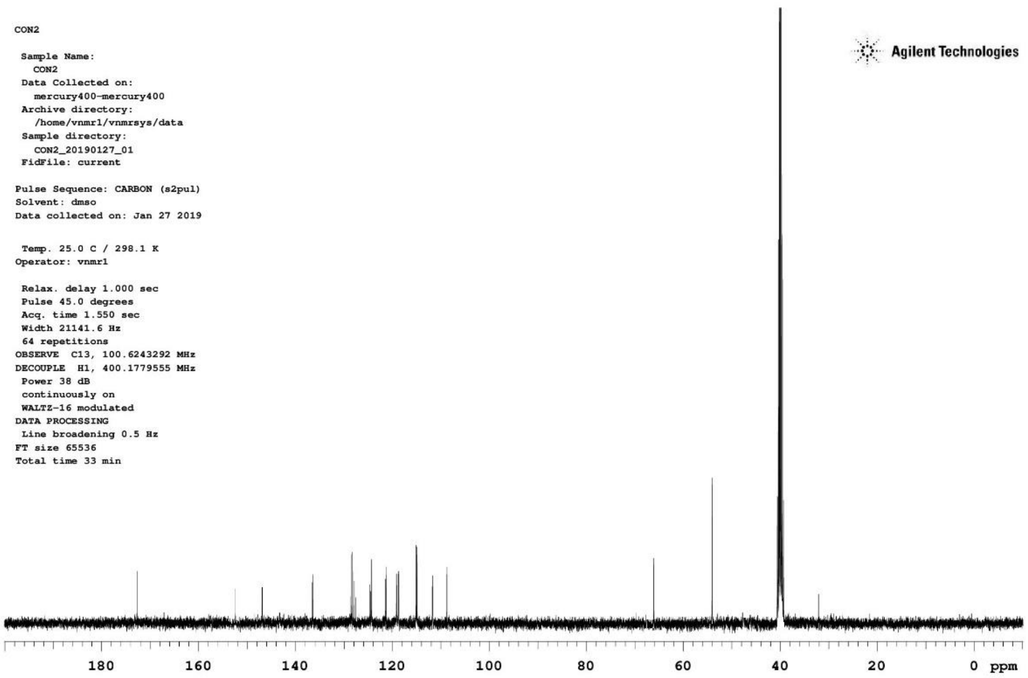

**Figure S8.** HRMS spectrum of compound **3b**

Formula Predictor Report - CON-2_6.lcd　　　　Page 1 of 1

Data File: C:\LabSolutions\Data\Analiz\AOzdemir\CON-2_6.lcd

| Elmt | Val. | Min | Max | Elmt | Val. | Min | Max | Elmt | Val. | Min | Max | Elmt | Val. | Min | Max | Use Adduct |
|---|---|---|---|---|---|---|---|---|---|---|---|---|---|---|---|---|
| H | 1 | 0 | 30 | O | 2 | 1 | 4 | S | 2 | 0 | 2 | Ru | 2 | 0 | 0 | H |
| C | 4 | 15 | 25 | F | 1 | 0 | 0 | Cl | 1 | 0 | 0 | Pd | 2 | 0 | 0 | |
| N | 3 | 1 | 4 | P | 3 | 0 | 0 | Br | 1 | 0 | 0 | I | 3 | 0 | 0 | |

| | | | | | |
|---|---|---|---|---|---|
| Error Margin (ppm): | 10 | DBE Range: | 10.0 - 20.0 | Electron Ions: | both |
| HC Ratio: | unlimited | Apply N Rule: | yes | Use MSn Info: | yes |
| Max Isotopes: | 3 | Isotope RI (%): | 1.00 | Isotope Res: | 9000 |
| MSn Iso RI (%): | 10.00 | MSn Logic Mode: | AND | Max Results: | 500 |

Event#: 1 MS(E+)　Ret. Time : 6.413　Scan# : 963

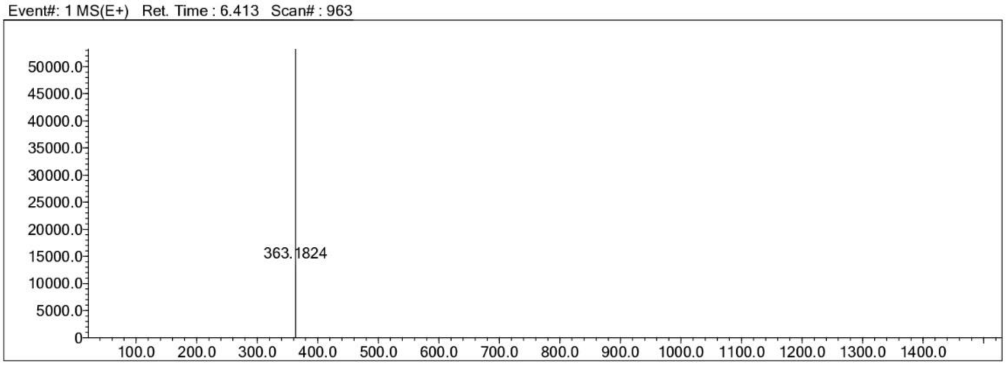


Measured region for 363.1824 m/z

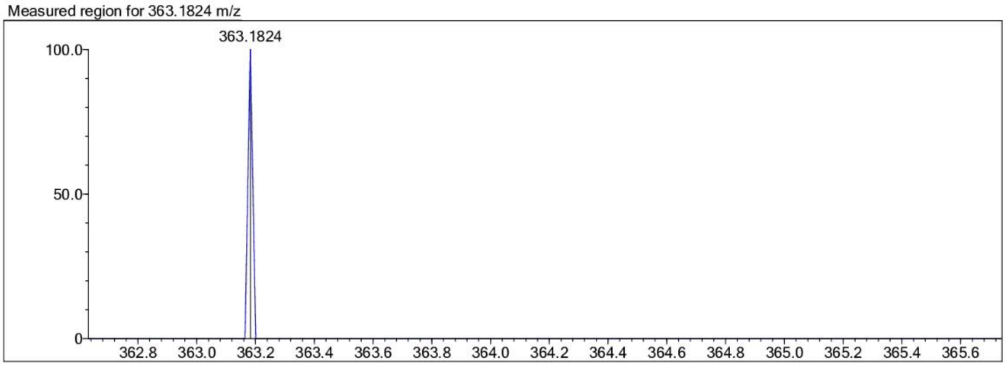


C21 H22 N4 O2 [M+H]+ : Predicted region for 363.1816 m/z

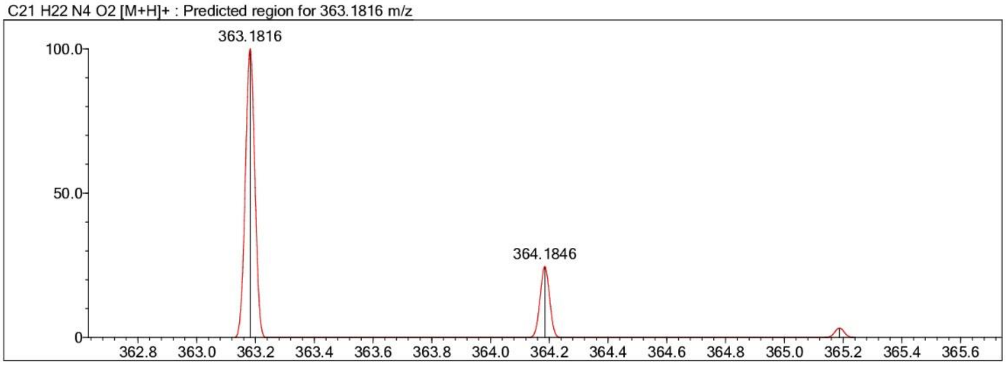


| Rank | Score | Formula (M) | Ion | Meas. m/z | Pred. m/z | Df. (mDa) | Df. (ppm) | Iso | DBE |
|---|---|---|---|---|---|---|---|---|---|
| 1 | 0.00 | C21 H22 N4 O2 | [M+H]+ | 363.1824 | 363.1816 | 0.8 | 2.20 | 0.00 | 13.0 |

**Figure S9.** IR spectrum of compound **3c**

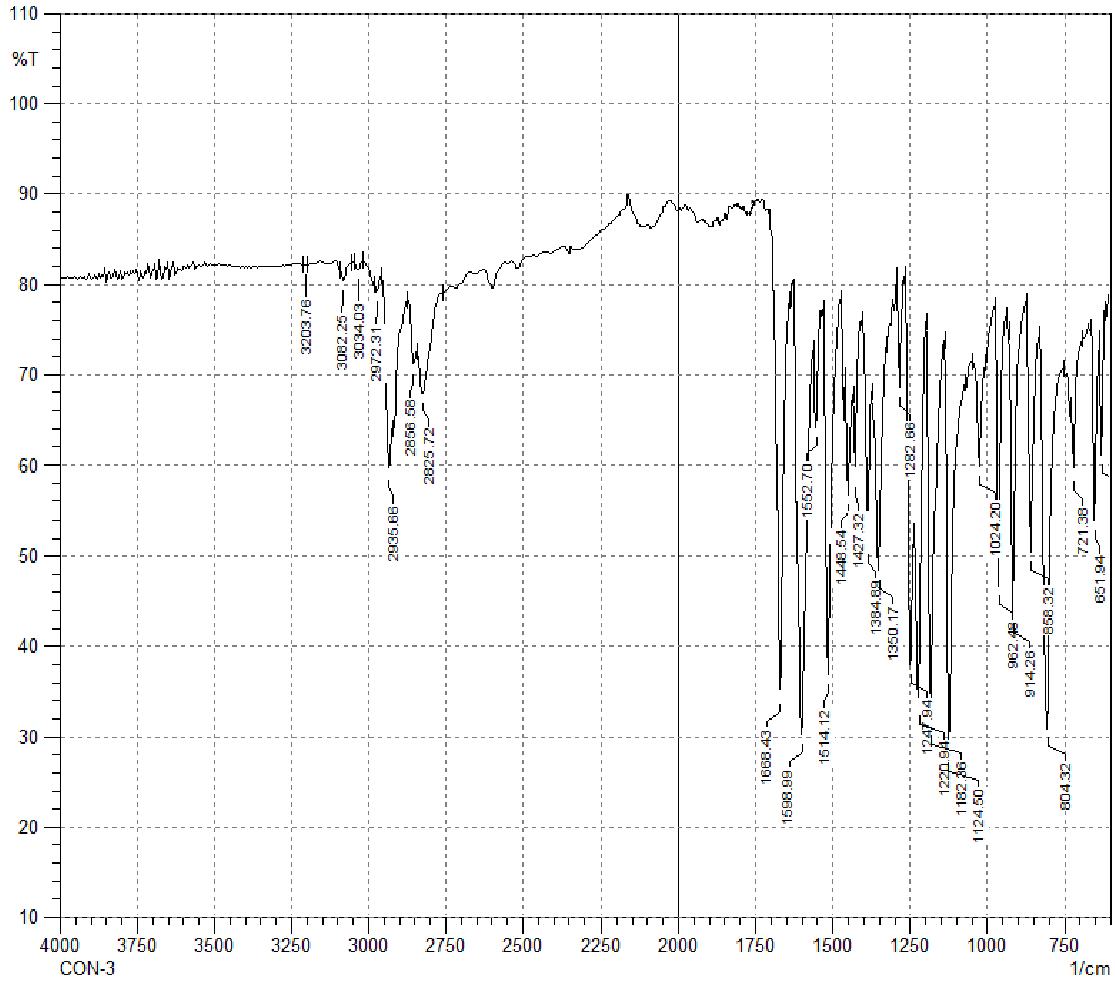

**Figure S10.** $^{1}$H NMR spectrum of compound **3c**

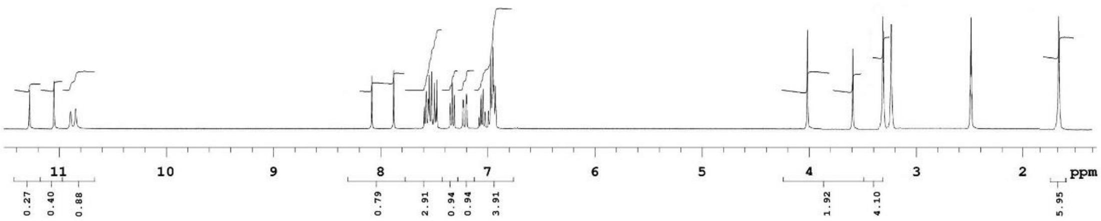


**Figure S11.** $^{13}$C NMR spectrum of compound **3c**

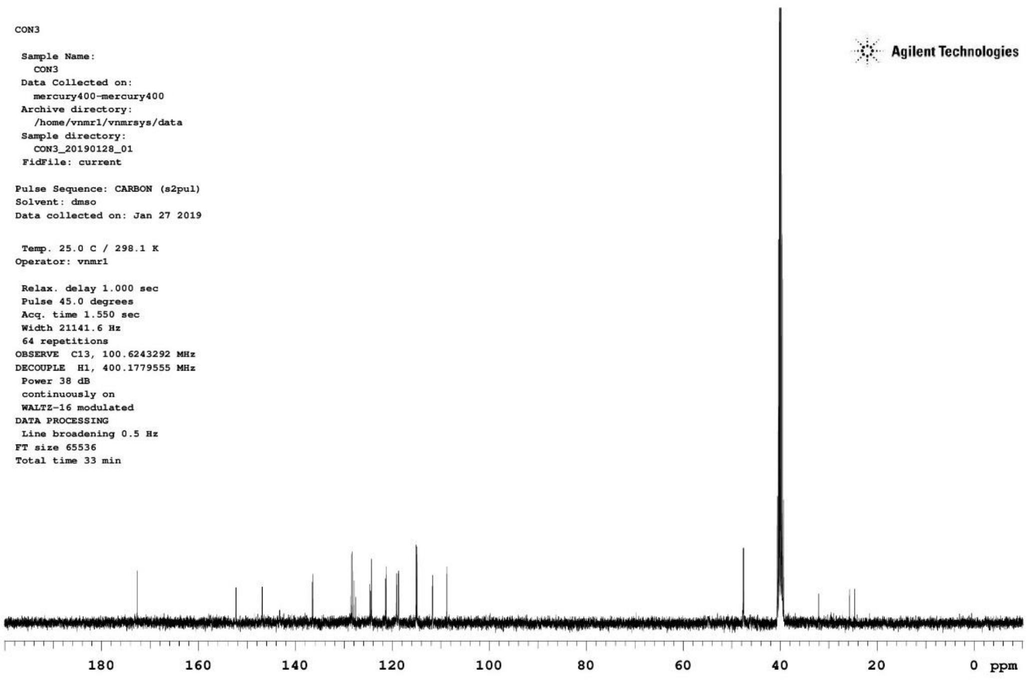

**Figure S12.** HRMS spectrum of compound **3c**

Formula Predictor Report - CON-3_7.lcd

Page 1 of 1

Data File: C:\LabSolutions\Data\Analiz\AOzdemir\CON-3_7.lcd

| Elmt | Val. | Min | Max | Elmt | Val. | Min | Max | Elmt | Val. | Min | Max | Elmt | Val. | Min | Max | Use Adduct |
|---|---|---|---|---|---|---|---|---|---|---|---|---|---|---|---|---|
| H | 1 | 0 | 30 | O | 2 | 1 | 4 | S | 2 | 0 | 2 | Ru | 2 | 0 | 0 | H |
| C | 4 | 15 | 25 | F | 1 | 0 | 0 | Cl | 1 | 0 | 0 | Pd | 2 | 0 | 0 | |
| N | 3 | 1 | 4 | P | 3 | 0 | 0 | Br | 1 | 0 | 0 | I | 3 | 0 | 0 | |

Error Margin (ppm): 10
HC Ratio: unlimited
Max Isotopes: 3
MSn Iso RI (%): 10.00

DBE Range: 10.0 - 20.0
Apply N Rule: yes
Isotope RI (%): 1.00
MSn Logic Mode: AND

Electron Ions: both
Use MSn Info: yes
Isotope Res: 9000
Max Results: 500

Event#: 1 MS(E+) Ret. Time : 6.400 -> 6.840 Scan# : 961 -> 1027

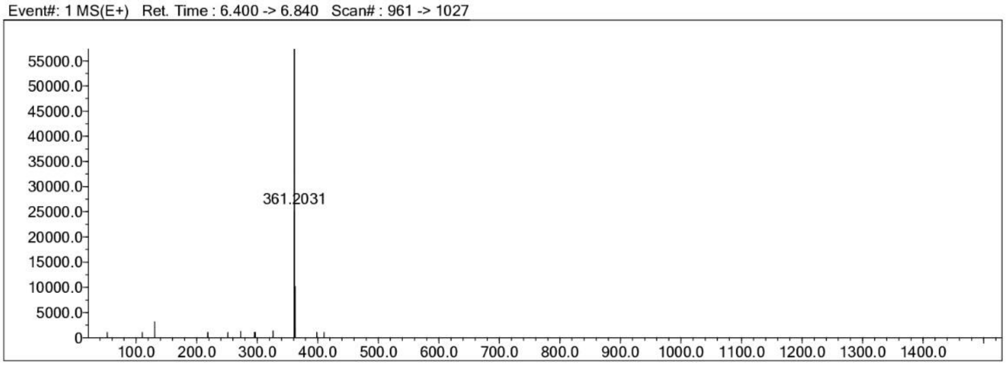


Measured region for 361.2031 m/z

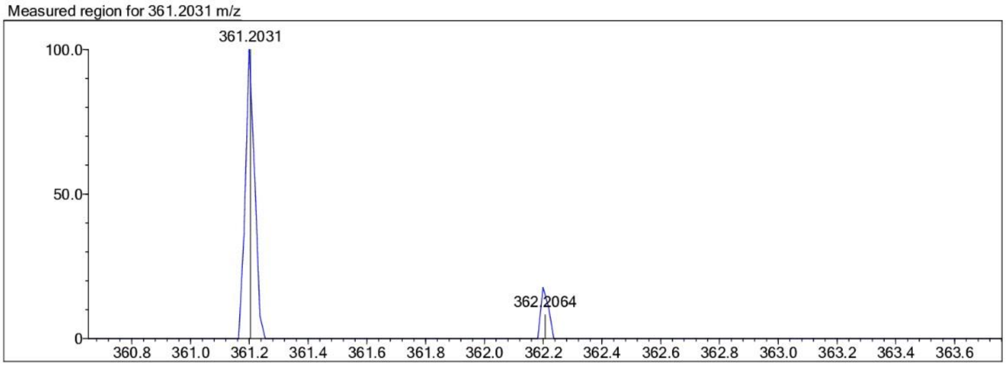


C22 H24 N4 O [M+H]+ : Predicted region for 361.2023 m/z

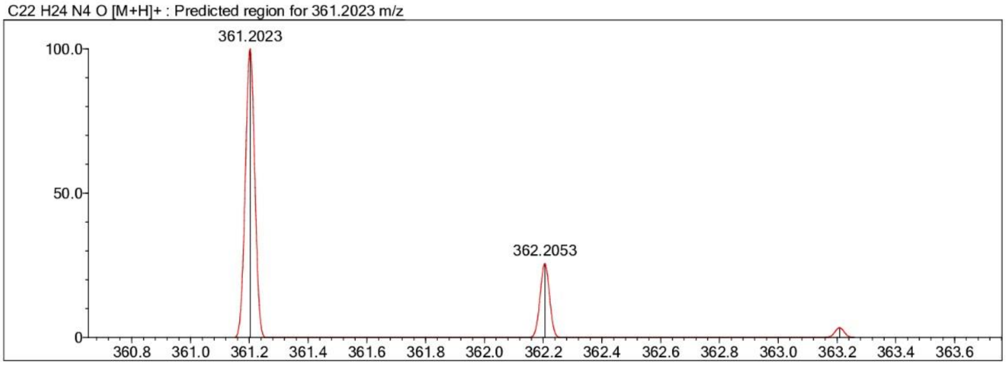


| Rank | Score | Formula (M) | Ion | Meas. m/z | Pred. m/z | Df. (mDa) | Df. (ppm) | Iso | DBE |
|---|---|---|---|---|---|---|---|---|---|
| 1 | 57.85 | C22 H24 N4 O | [M+H]+ | 361.2031 | 361.2023 | 0.8 | 2.21 | 59.65 | 13.0 |

**Figure S13.** IR spectrum of compound **3d**

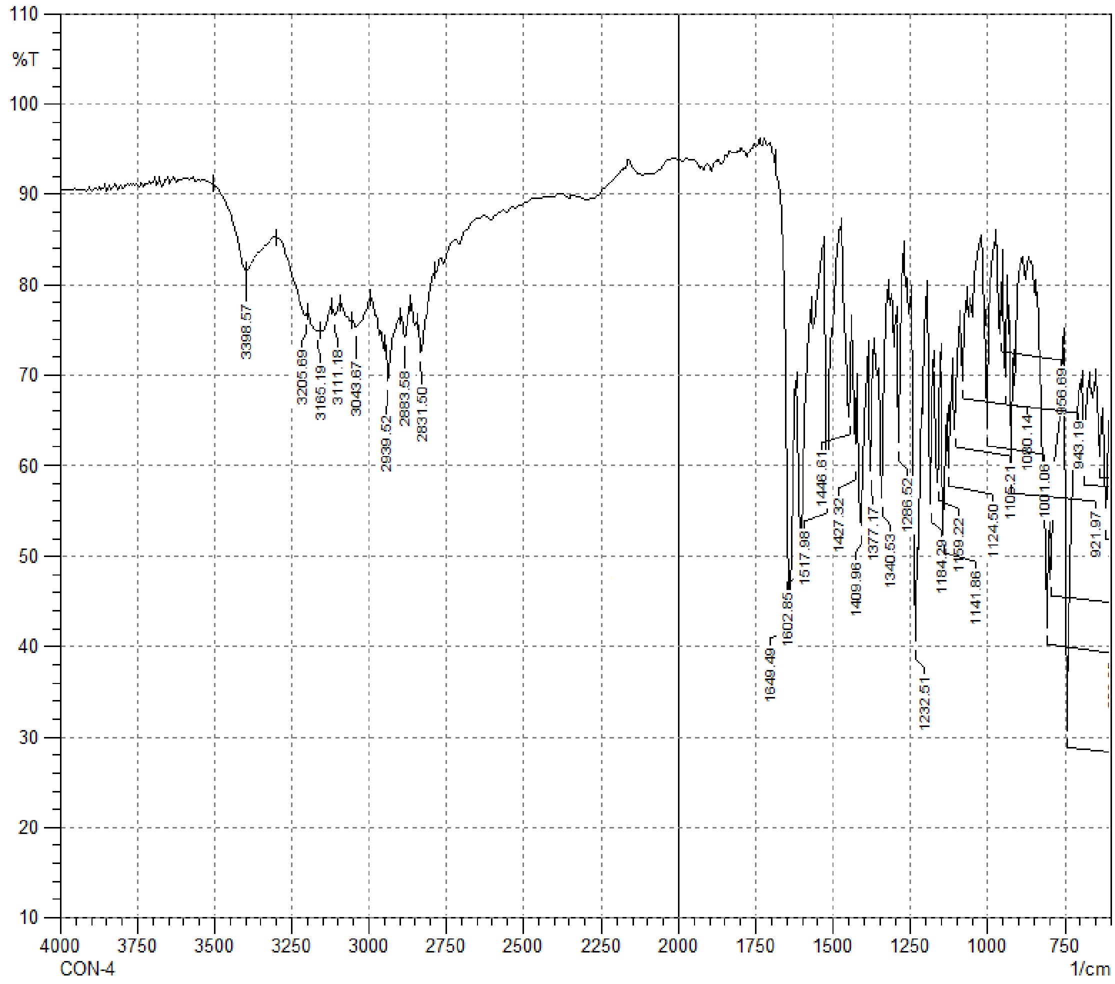

**Figure S14.** $^{1}$H NMR spectrum of compound **3d**

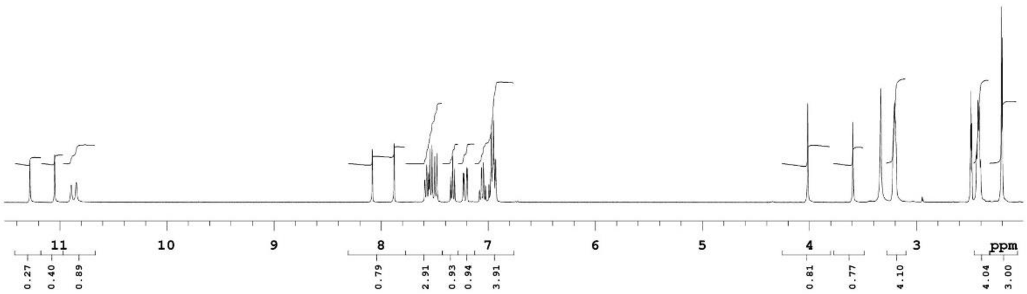


**Figure S15.** $^{13}$C NMR spectrum of compound **3d**

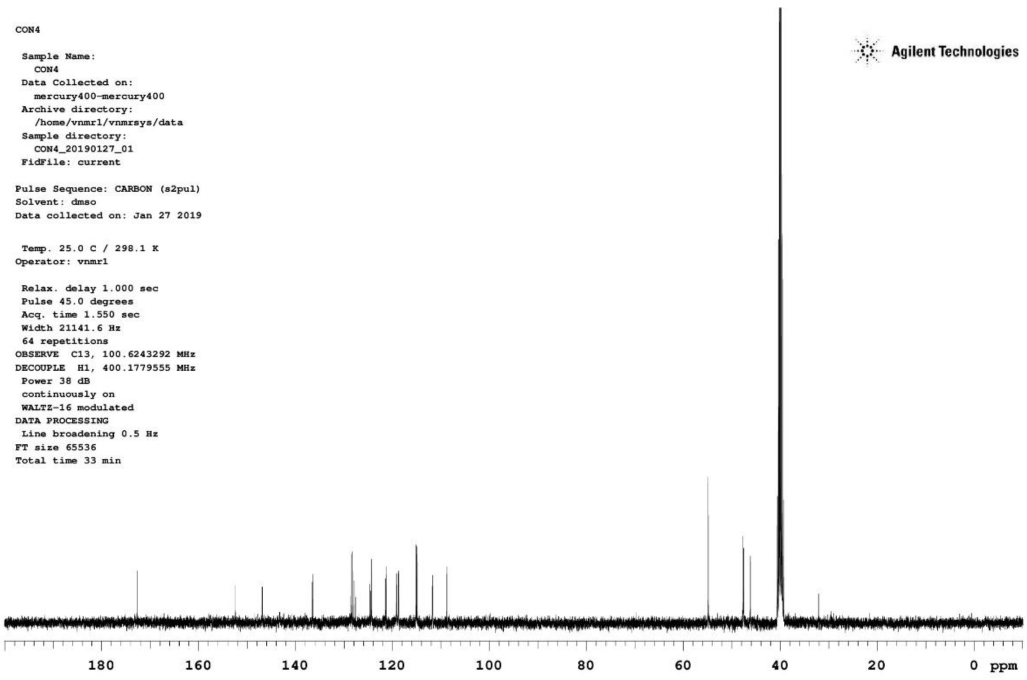

**Figure S16.** HRMS spectrum of compound **3d**

Formula Predictor Report - CON-5_7.lcd  Page 1 of 1

Data File: C:\LabSolutions\Data\Analiz\AOzdemir\CON-5_7.lcd

| Elmt | Val. | Min | Max | Elmt | Val. | Min | Max | Elmt | Val. | Min | Max | Elmt | Val. | Min | Max | Use Adduct |
|---|---|---|---|---|---|---|---|---|---|---|---|---|---|---|---|---|
| H | 1 | 0 | 30 | O | 2 | 0 | 5 | S | 2 | 0 | 0 | Ru | 2 | 0 | 0 | H |
| C | 4 | 15 | 25 | F | 1 | 0 | 0 | Cl | 1 | 0 | 0 | Pd | 2 | 0 | 0 | |
| N | 3 | 1 | 5 | P | 3 | 0 | 0 | Br | 1 | 0 | 0 | I | 3 | 0 | 0 | |

| | | | | | |
|---|---|---|---|---|---|
| Error Margin (ppm): | 5 | DBE Range: | 8.0 - 17.0 | Electron Ions: | both |
| HC Ratio: | unlimited | Apply N Rule: | yes | Use MSn Info: | yes |
| Max Isotopes: | 3 | Isotope RI (%): | 1.00 | Isotope Res: | 9000 |
| MSn Iso RI (%): | 10.00 | MSn Logic Mode: | AND | Max Results: | 500 |

Event#: 1 MS(E+)  Ret. Time : 5.227  Scan# : 785

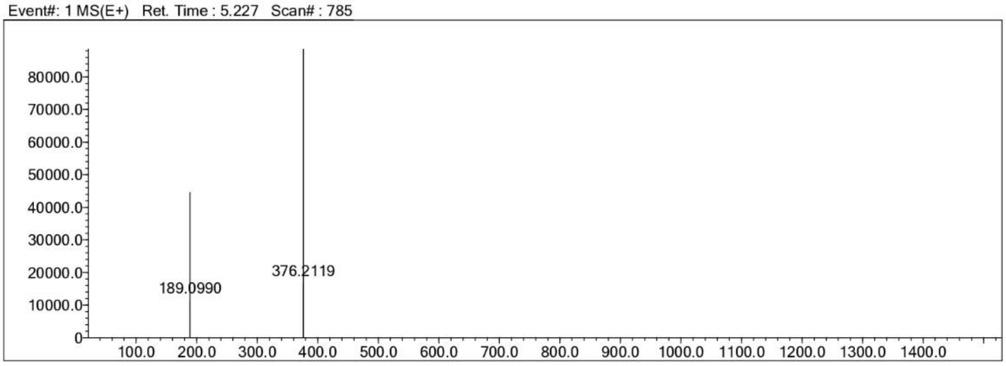


Measured region for 376.2148 m/z

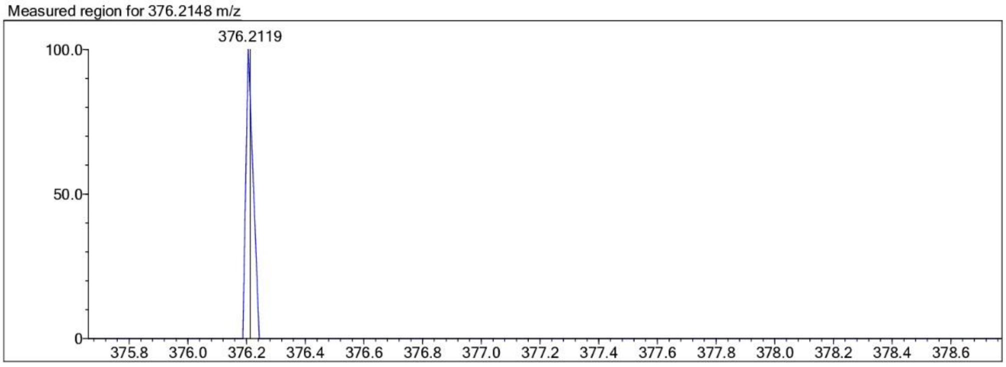


C22 H25 N5 O [M+H]+ : Predicted region for 376.2132 m/z

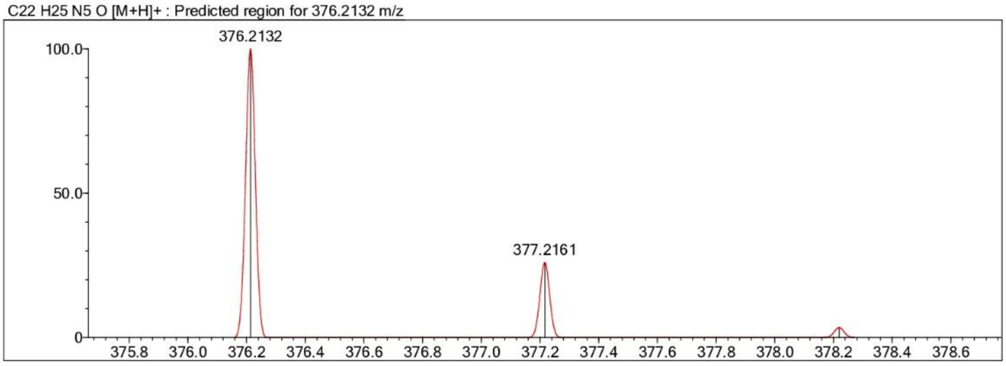


| Rank | Score | Formula (M) | Ion | Meas. m/z | Pred. m/z | Df. (mDa) | Df. (ppm) | Iso | DBE |
|---|---|---|---|---|---|---|---|---|---|
| 1 | 0.00 | C22 H25 N5 O | [M+H]+ | 376.2148 | 376.2132 | 1.6 | 4.25 | 0.00 | 13.0 |

**Figure S17.** IR spectrum of compound **3e**

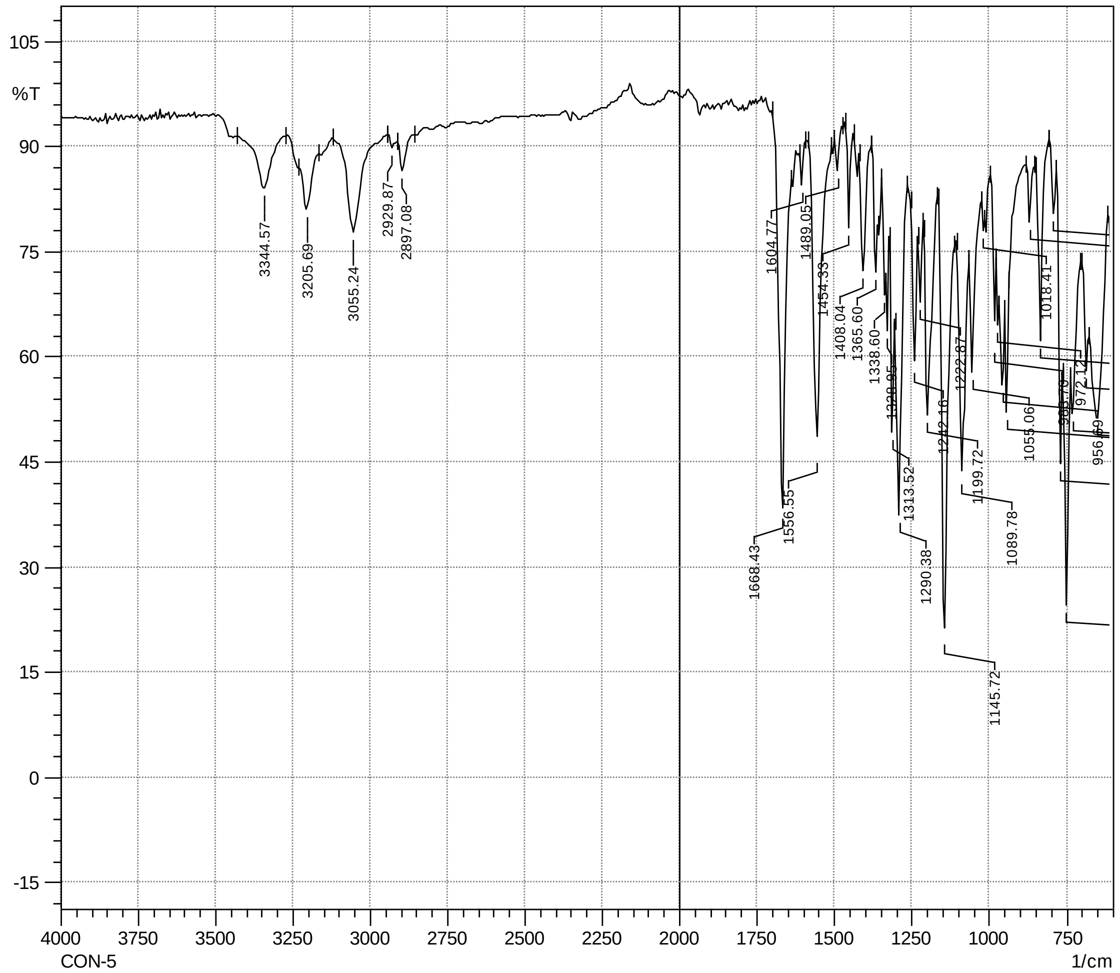

**Figure S18.** $^{1}$H NMR spectrum of compound **3e**

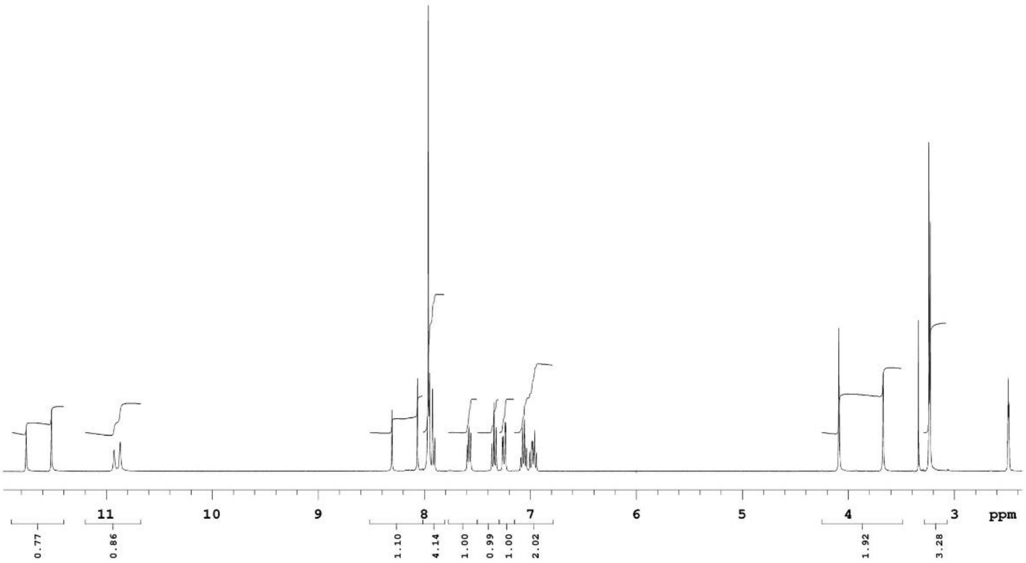


**Figure S19.** $^{13}$C NMR spectrum of compound **3e**

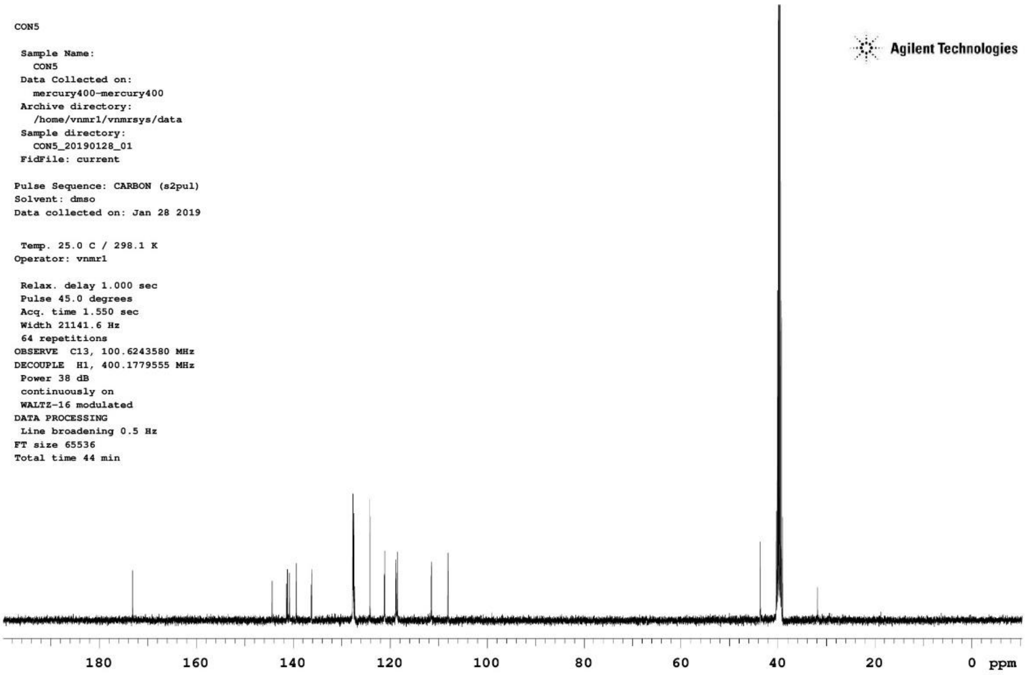

**Figure S20.** HRMS spectrum of compound **3e**

Formula Predictor Report - CON-7_8.lcd



Data File: C:\LabSolutions\Data\Analiz\AOzdemir\CON-7_8.lcd

| Elmt | Val. | Min | Max | Elmt | Val. | Min | Max | Elmt | Val. | Min | Max | Elmt | Val. | Min | Max | Use Adduct |
|---|---|---|---|---|---|---|---|---|---|---|---|---|---|---|---|---|
| H | 1 | 0 | 30 | O | 2 | 0 | 5 | S | 2 | 0 | 1 | Ru | 2 | 0 | 0 | H |
| C | 4 | 15 | 25 | F | 1 | 0 | 0 | Cl | 1 | 0 | 0 | Pd | 2 | 0 | 0 | |
| N | 3 | 1 | 5 | P | 3 | 0 | 0 | Br | 1 | 0 | 0 | I | 3 | 0 | 0 | |

Error Margin (ppm): 5
HC Ratio: unlimited
Max Isotopes: 3
MSn Iso RI (%): 10.00

DBE Range: 8.0 - 17.0
Apply N Rule: yes
Isotope RI (%): 1.00
MSn Logic Mode: AND

Electron Ions: both
Use MSn Info: yes
Isotope Res: 9000
Max Results: 500

Event#: 1 MS(E+) Ret. Time : 6.133 Scan# : 921

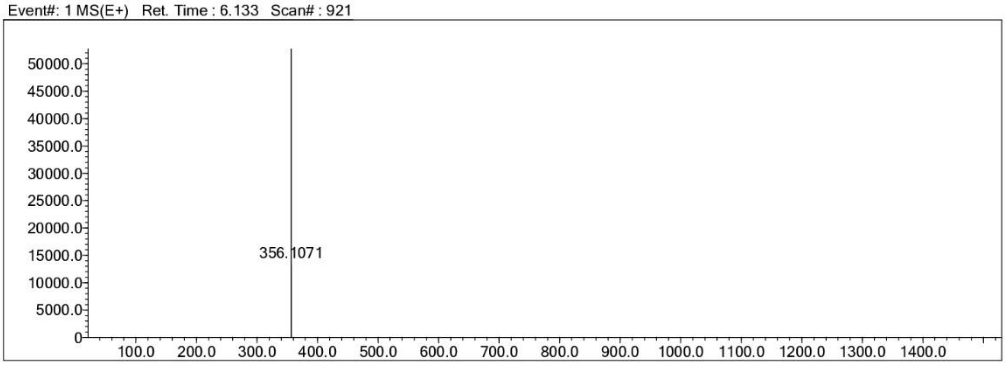


Measured region for 356.1071 m/z

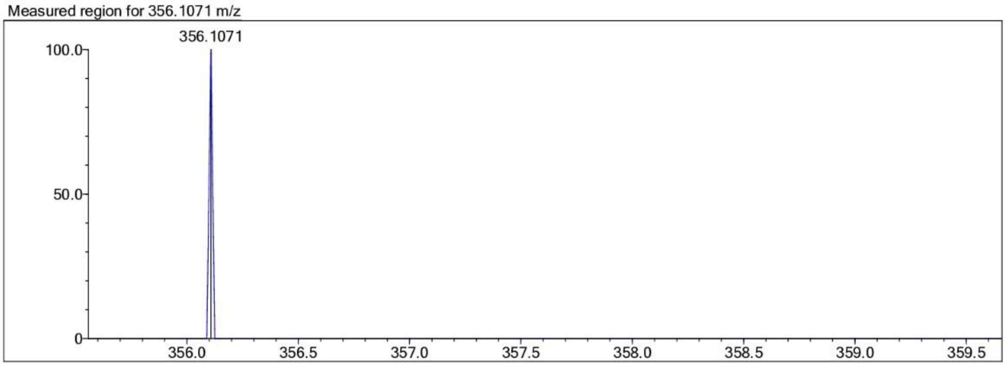


C18 H17 N3 O3 S [M+H]+ : Predicted region for 356.1063 m/z

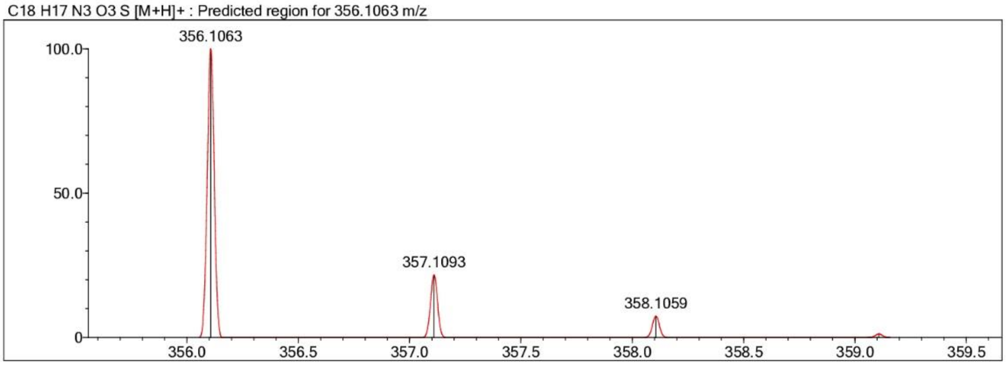


| Rank | Score | Formula (M) | Ion | Meas. m/z | Pred. m/z | Df. (mDa) | Df. (ppm) | Iso | DBE |
|---|---|---|---|---|---|---|---|---|---|
| 1 | 0.00 | C18 H17 N3 O3 S | [M+H]+ | 356.1071 | 356.1063 | 0.8 | 2.25 | 0.00 | 12.0 |

**Figure S21.** IR spectrum of compound **3f**

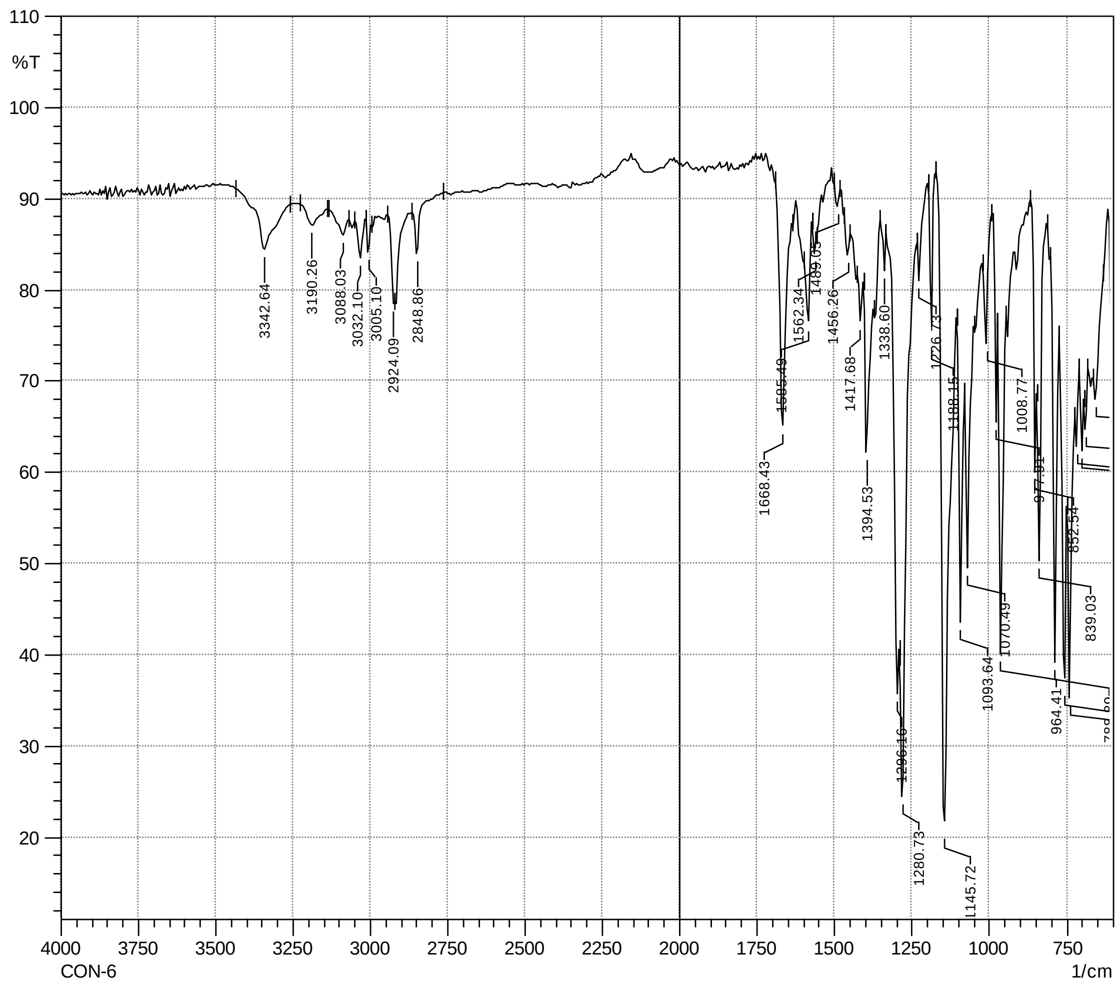

**Figure S22.** $^{1}$H NMR spectrum of compound **3f**

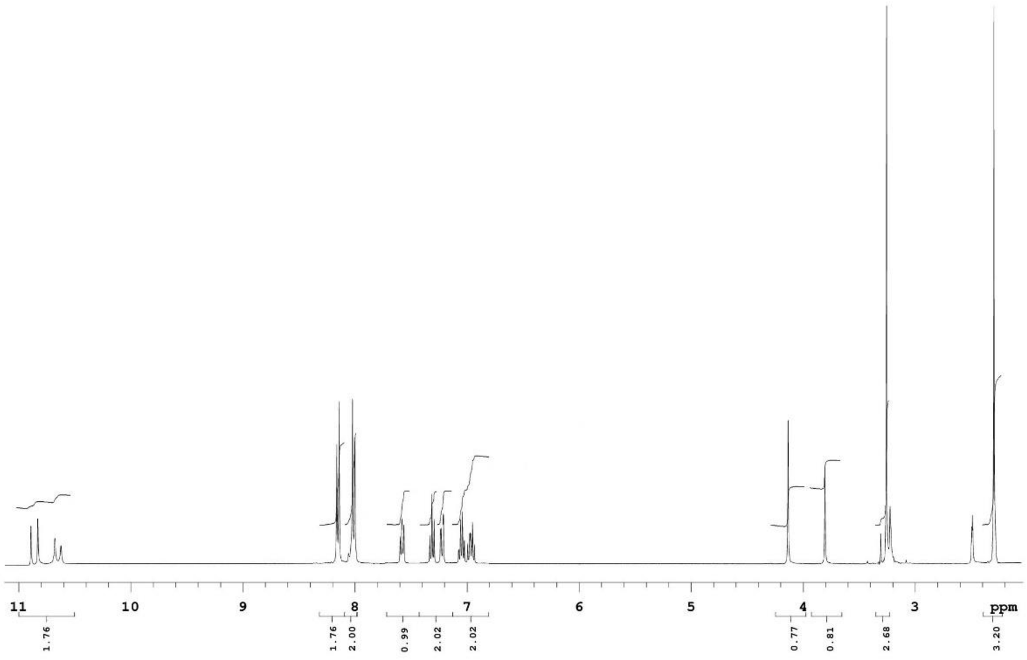


**Figure S23.** $^{13}$C NMR spectrum of compound **3f**

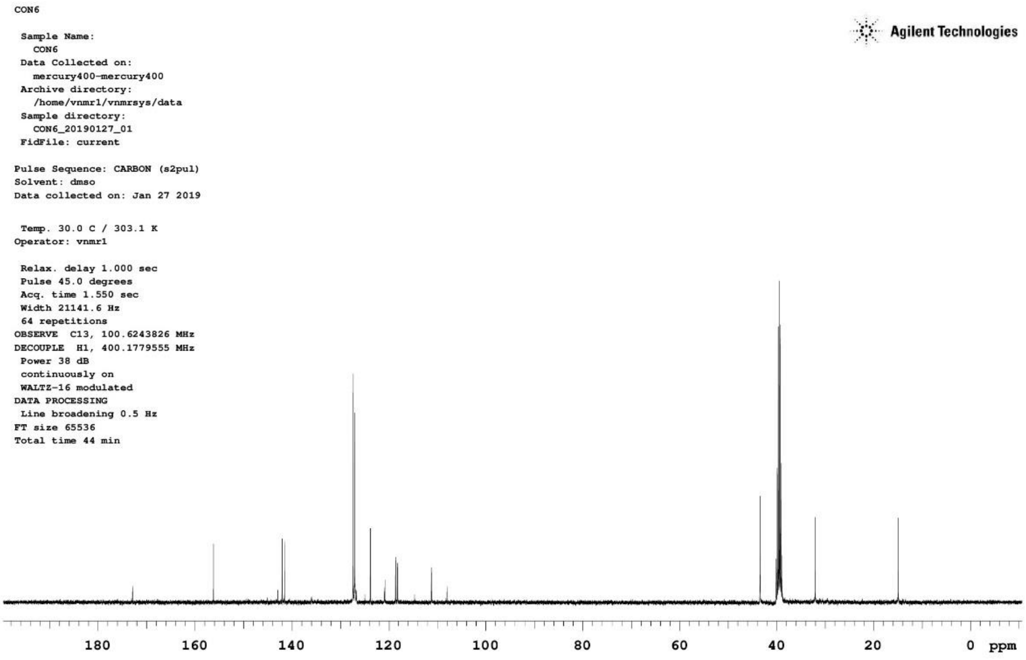

**Figure S24.** HRMS spectrum of compound **3f**

Formula Predictor Report - CON-8_9.lcd Page 1 of 1

Data File: C:\LabSolutions\Data\Analiz\AOzdemir\CON-8_9.lcd

| Elmt | Val. | Min | Max | Elmt | Val. | Min | Max | Elmt | Val. | Min | Max | Elmt | Val. | Min | Max | Use Adduct |
|---|---|---|---|---|---|---|---|---|---|---|---|---|---|---|---|---|
| H | 1 | 0 | 30 | O | 2 | 0 | 5 | S | 2 | 1 | 1 | Ru | 2 | 0 | 0 | H |
| C | 4 | 15 | 25 | F | 1 | 0 | 0 | Cl | 1 | 0 | 0 | Pd | 2 | 0 | 0 | |
| N | 3 | 1 | 5 | P | 3 | 0 | 0 | Br | 1 | 0 | 0 | I | 3 | 0 | 0 | |

| | | | | | |
|---|---|---|---|---|---|
| Error Margin (ppm): | 5 | DBE Range: | 8.0 - 17.0 | Electron Ions: | both |
| HC Ratio: | unlimited | Apply N Rule: | yes | Use MSn Info: | yes |
| Max Isotopes: | 3 | Isotope RI (%): | 1.00 | Isotope Res: | 9000 |
| MSn Iso RI (%): | 10.00 | MSn Logic Mode: | AND | Max Results: | 500 |

Event#: 1 MS(E+) Ret. Time : 6.253 Scan# : 939

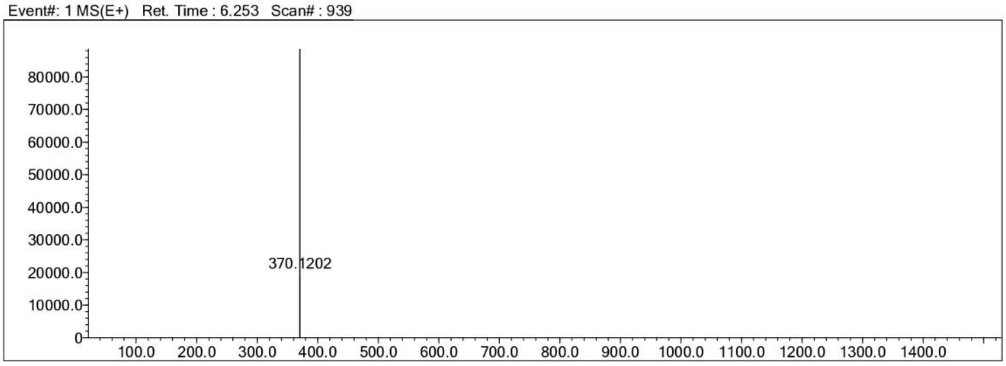


Measured region for 370.1202 m/z

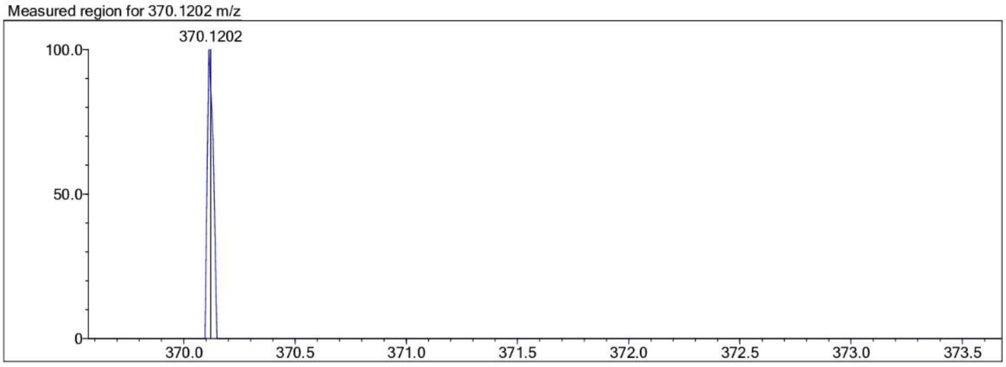


C19 H19 N3 O3 S [M+H]+ : Predicted region for 370.1220 m/z

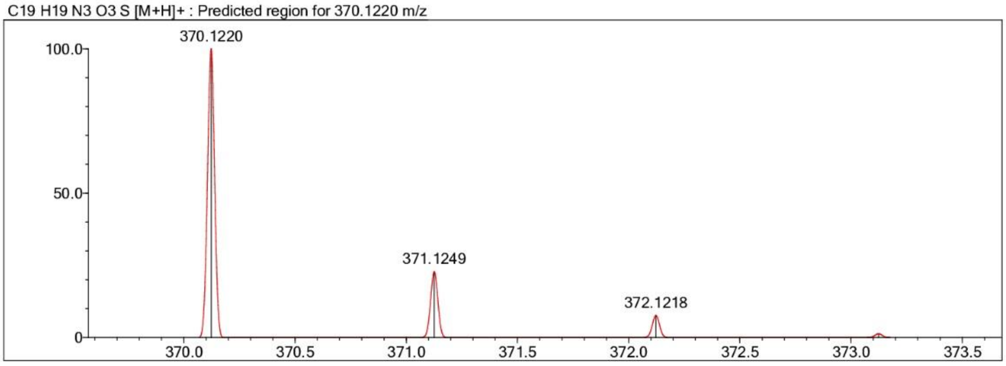


| Rank | Score | Formula (M) | Ion | Meas. m/z | Pred. m/z | Df. (mDa) | Df. (ppm) | Iso | DBE |
|---|---|---|---|---|---|---|---|---|---|
| 1 | 0.00 | C19 H19 N3 O3 S | [M+H]+ | 370.1202 | 370.1220 | -1.8 | -4.86 | 0.00 | 12.0 |

**Figure S25.** IR spectrum of compound **3g**

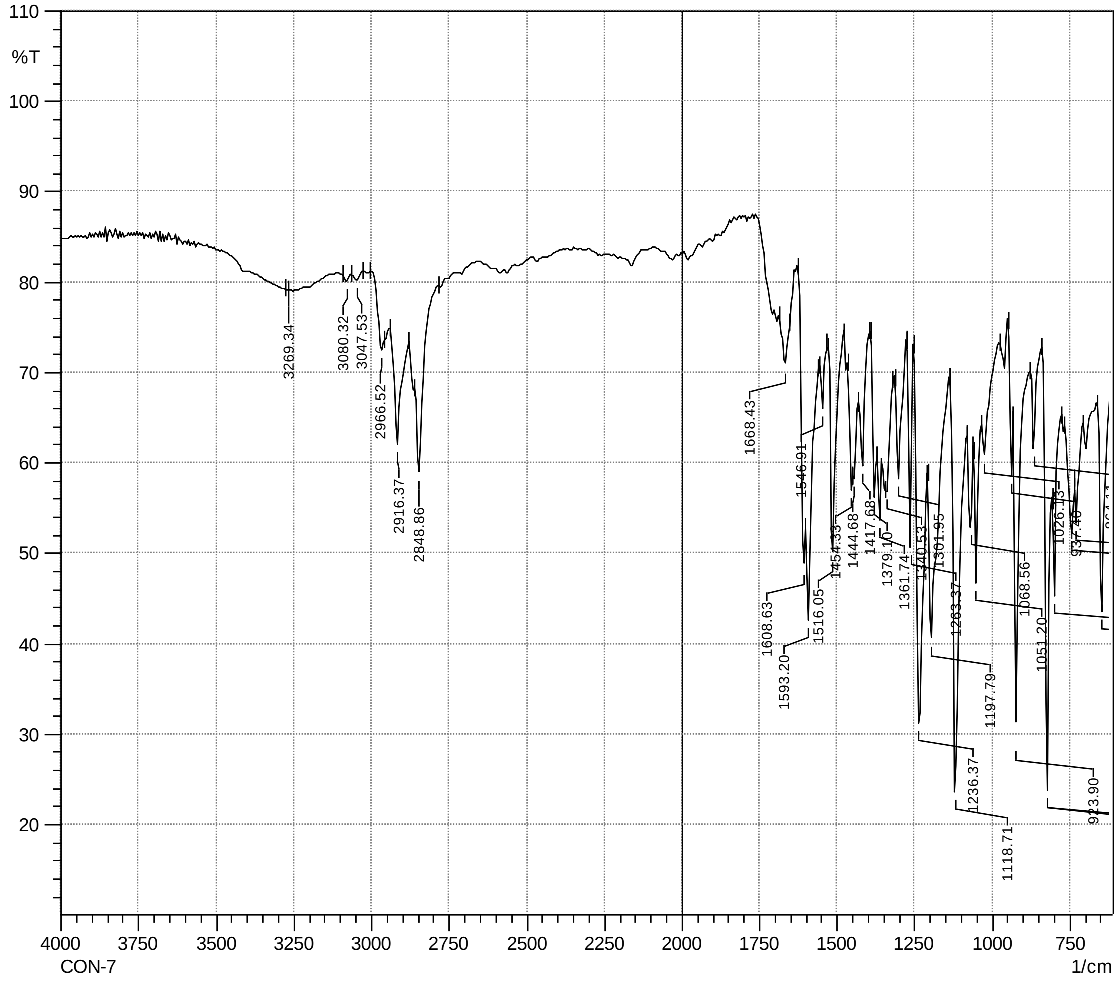

**Figure S26.** $^{1}$H NMR spectrum of compound **3g**

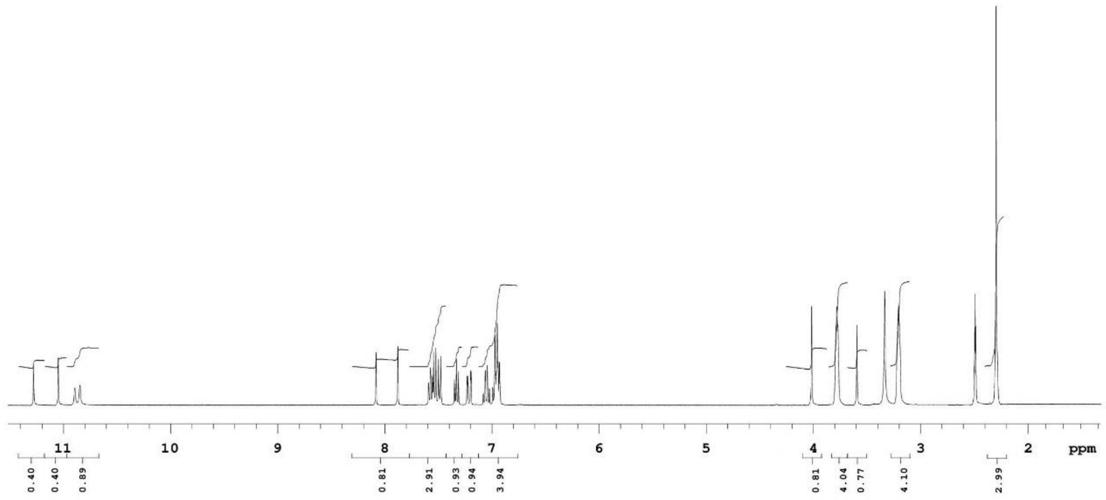


**Figure S27.** $^{13}$C NMR spectrum of compound **3g**

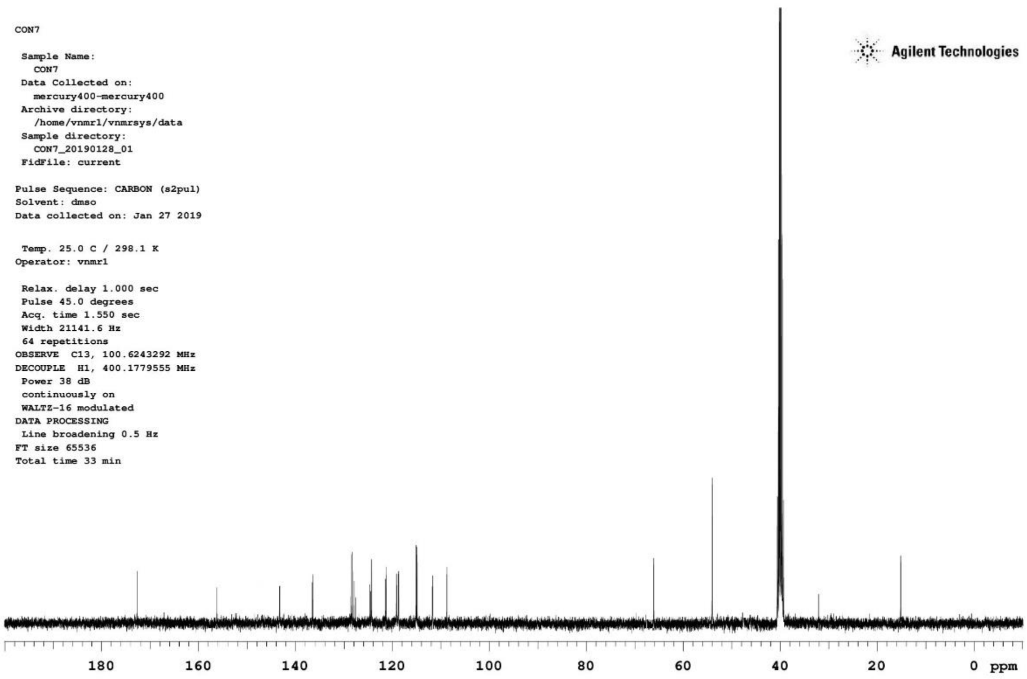

**Figure S28.** HRMS spectrum of compound **3g**

Formula Predictor Report - CON-9_8.lcd

Page 1 of 1

Data File: C:\LabSolutions\Data\Analiz\AOzdemir\CON-9_8.lcd

| Elmt | Val. | Min | Max | Elmt | Val. | Min | Max | Elmt | Val. | Min | Max | Elmt | Val. | Min | Max | Use Adduct |
|---|---|---|---|---|---|---|---|---|---|---|---|---|---|---|---|---|
| H | 1 | 0 | 30 | O | 2 | 1 | 4 | S | 2 | 0 | 2 | Ru | 2 | 0 | 0 | H |
| C | 4 | 15 | 25 | F | 1 | 0 | 0 | Cl | 1 | 0 | 0 | Pd | 2 | 0 | 0 | |
| N | 3 | 1 | 4 | P | 3 | 0 | 0 | Br | 1 | 0 | 0 | I | 3 | 0 | 0 | |

Error Margin (ppm): 10
HC Ratio: unlimited
Max Isotopes: 3
MSn Iso RI (%): 10.00

DBE Range: 10.0 - 20.0
Apply N Rule: yes
Isotope RI (%): 1.00
MSn Logic Mode: AND

Electron Ions: both
Use MSn Info: yes
Isotope Res: 9000
Max Results: 500

Event#: 1 MS(E+) Ret. Time : 6.493 -> 6.827 Scan# : 975 -> 1025

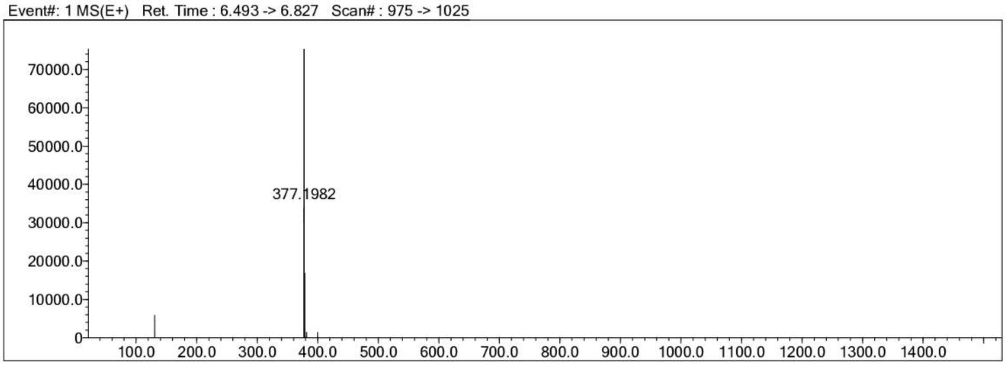


Measured region for 377.1982 m/z

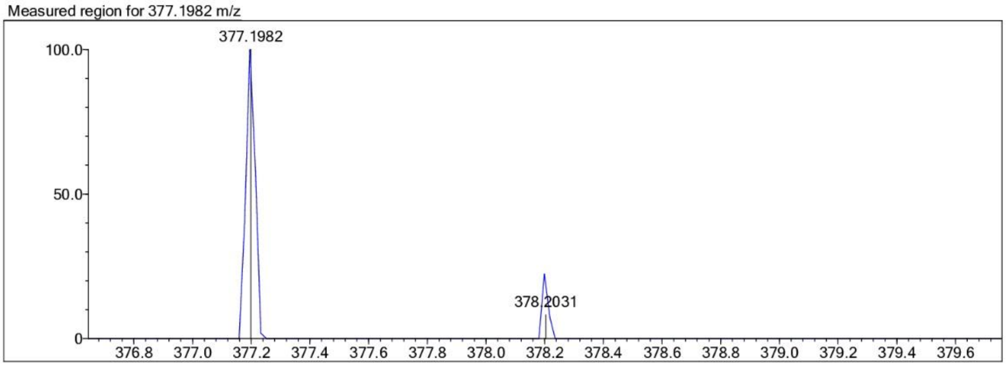


C22 H24 N4 O2 [M+H]+ : Predicted region for 377.1972 m/z

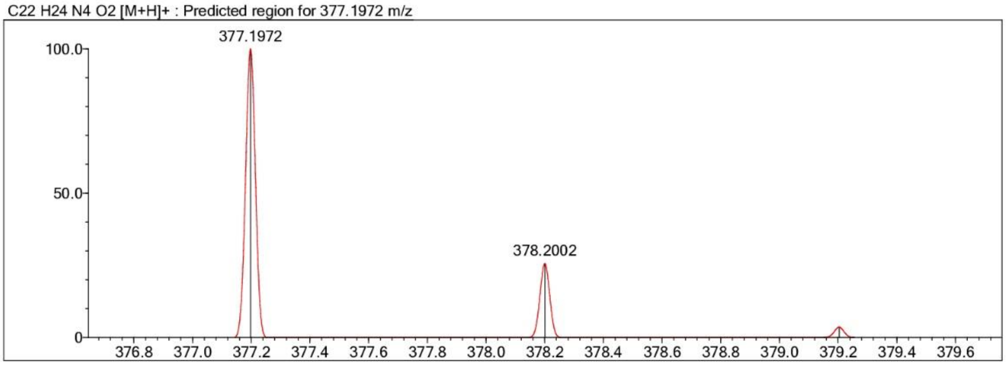


| Rank | Score | Formula (M) | Ion | Meas. m/z | Pred. m/z | Df. (mDa) | Df. (ppm) | Iso | DBE |
|---|---|---|---|---|---|---|---|---|---|
| 1 | 69.48 | C22 H24 N4 O2 | [M+H]+ | 377.1982 | 377.1972 | 1.0 | 2.65 | 72.47 | 13.0 |

**Figure S29.** IR spectrum of compound **3h**

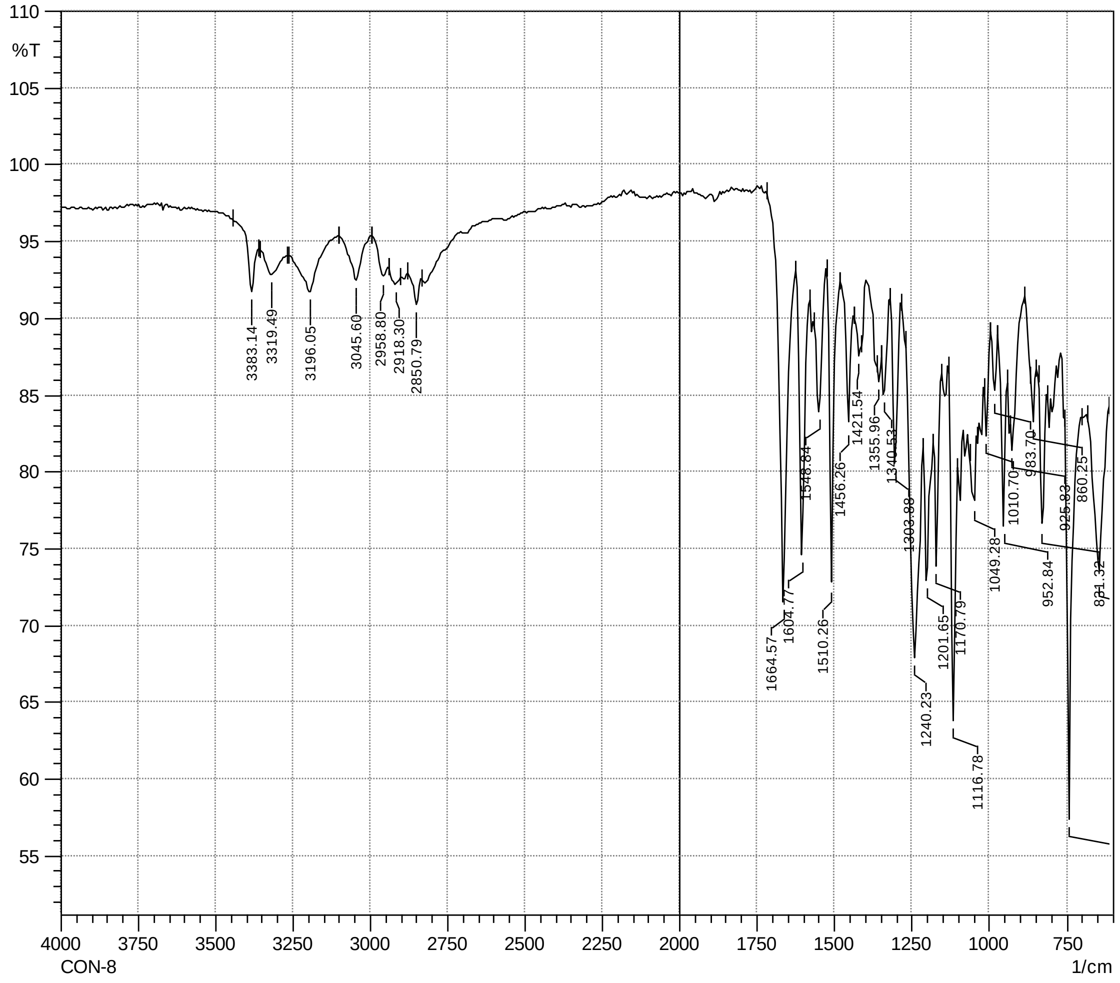

**Figure S30.** $^{1}$H NMR spectrum of compound **3h**

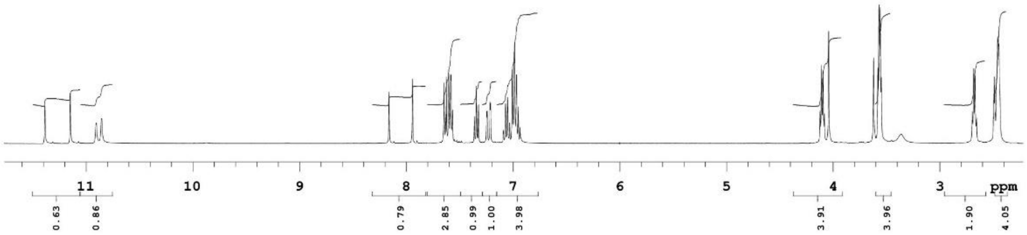


**Figure S31.** $^{13}$C NMR spectrum of compound **3h**

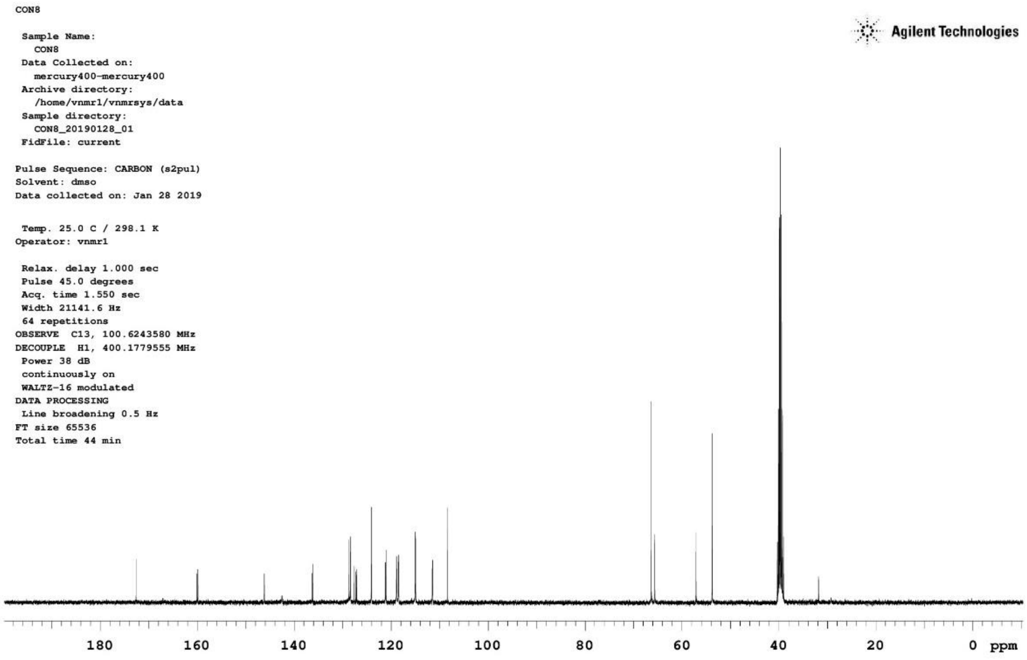

**Figure S32.** HRMS spectrum of compound **3h**

Formula Predictor Report - CON-12_11.lcd

Page 1 of 1

Data File: C:\LabSolutions\Data\Analiz\AOzdemir\CON-12_11.lcd

| Elmt | Val. | Min | Max | Elmt | Val. | Min | Max | Elmt | Val. | Min | Max | Elmt | Val. | Min | Max | Use Adduct |
|---|---|---|---|---|---|---|---|---|---|---|---|---|---|---|---|---|
| H | 1 | 0 | 30 | O | 2 | 0 | 5 | S | 2 | 0 | 1 | Ru | 2 | 0 | 0 | H |
| C | 4 | 15 | 25 | F | 1 | 0 | 0 | Cl | 1 | 0 | 0 | Pd | 2 | 0 | 0 | |
| N | 3 | 1 | 5 | P | 3 | 0 | 0 | Br | 1 | 0 | 0 | I | 3 | 0 | 0 | |

| | | | | | |
|---|---|---|---|---|---|
| Error Margin (ppm): | 5 | DBE Range: | 5.0 - 20.0 | Electron Ions: | both |
| HC Ratio: | unlimited | Apply N Rule: | yes | Use MSn Info: | yes |
| Max Isotopes: | 3 | Isotope RI (%): | 1.00 | Isotope Res: | 9000 |
| MSn Iso RI (%): | 10.00 | MSn Logic Mode: | AND | Max Results: | 500 |

Event#: 1 MS(E+) Ret. Time : 5.427 Scan# : 815

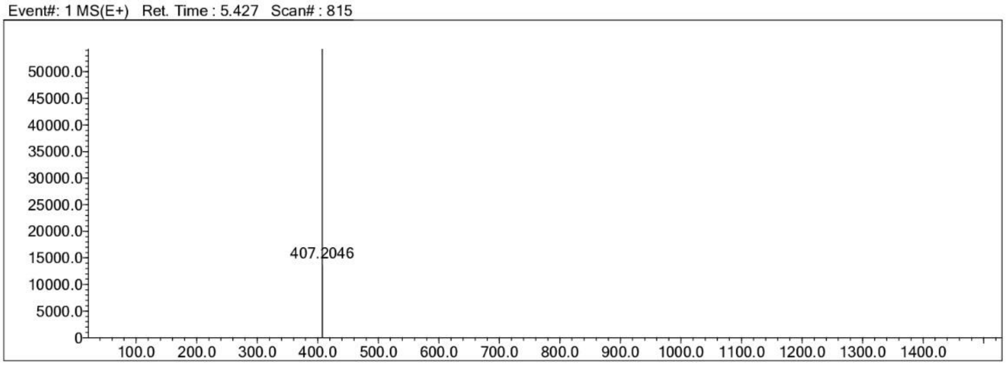


Measured region for 407.2071 m/z

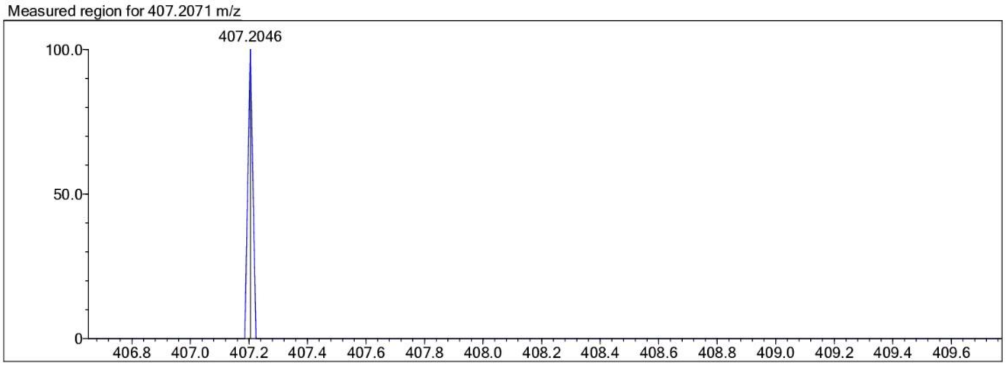


C23 H26 N4 O3 [M+H]+ : Predicted region for 407.2078 m/z

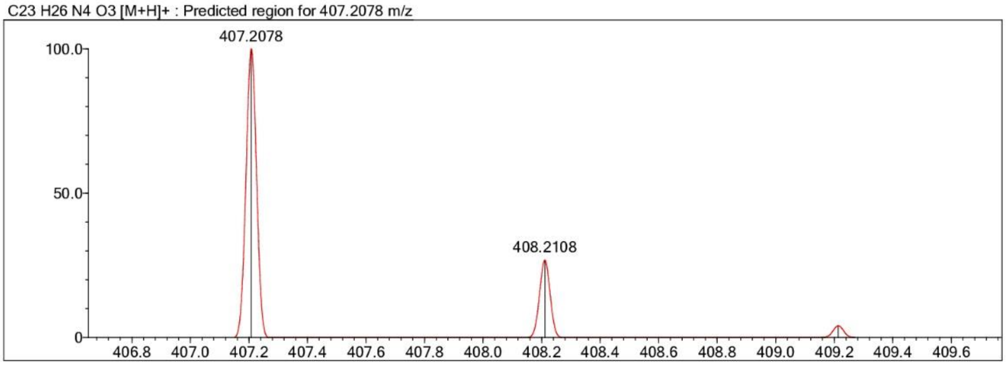


| Rank | Score | Formula (M) | Ion | Meas. m/z | Pred. m/z | Df. (mDa) | Df. (ppm) | Iso | DBE |
|---|---|---|---|---|---|---|---|---|---|
| 1 | 0.00 | C23 H26 N4 O3 | [M+H]+ | 407.2071 | 407.2078 | -0.7 | -1.72 | 0.00 | 13.0 |

**Figure S33.** IR spectrum of compound **3i**

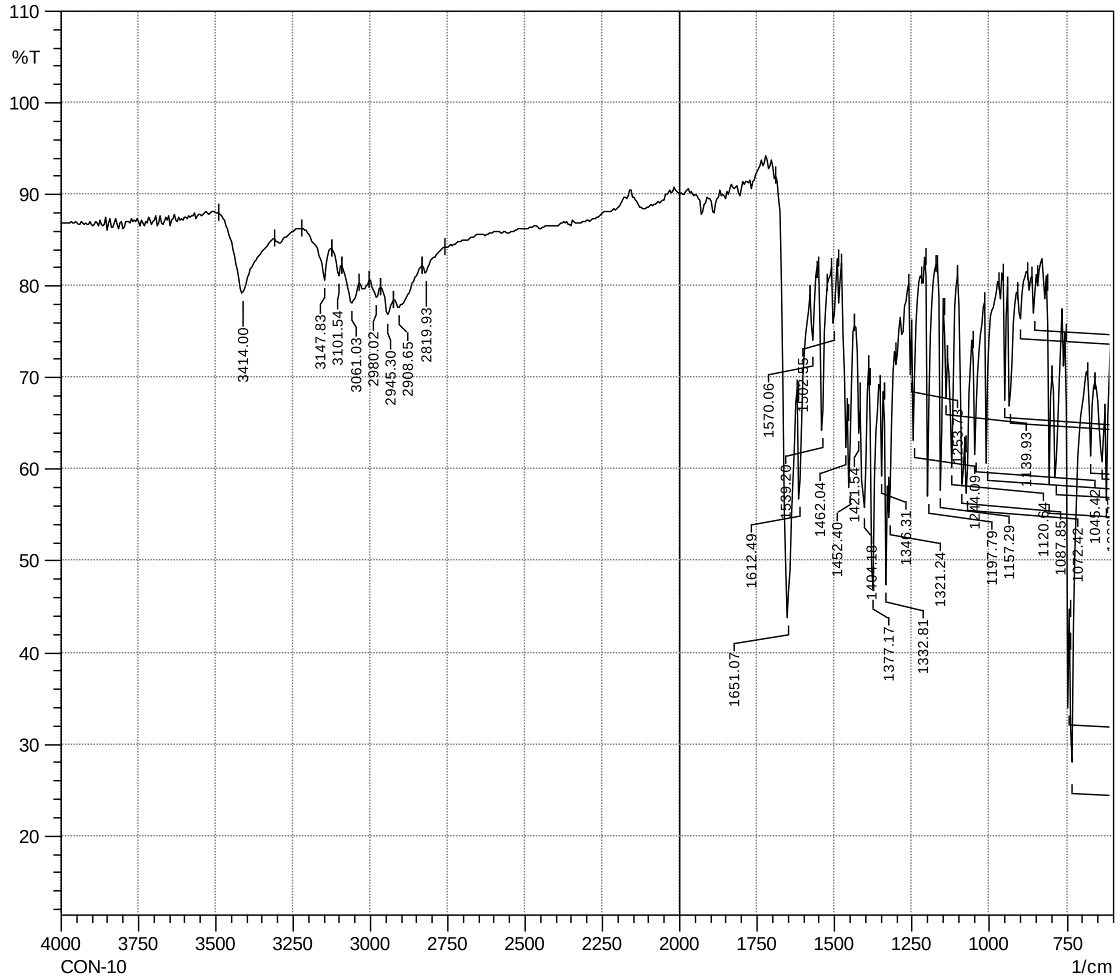

**Figure S34.** $^{1}$H NMR spectrum of compound **3i**

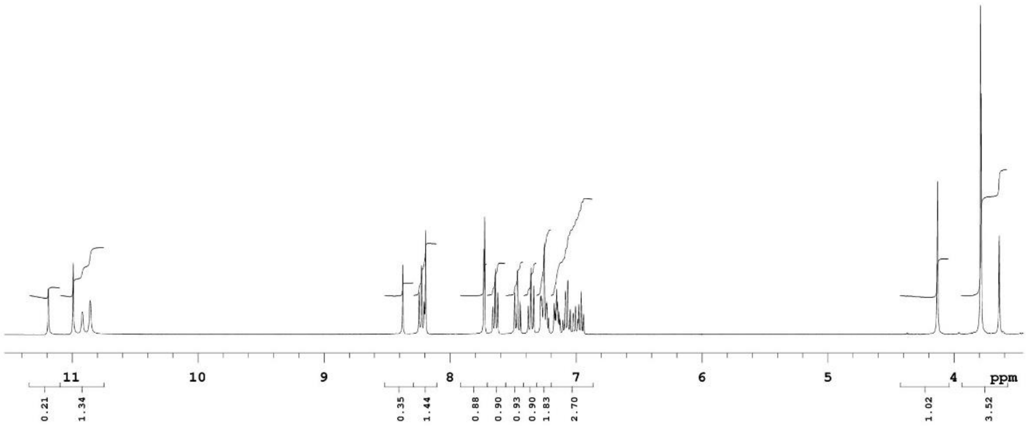


**Figure S35.** $^{13}$C NMR spectrum of compound **3i**

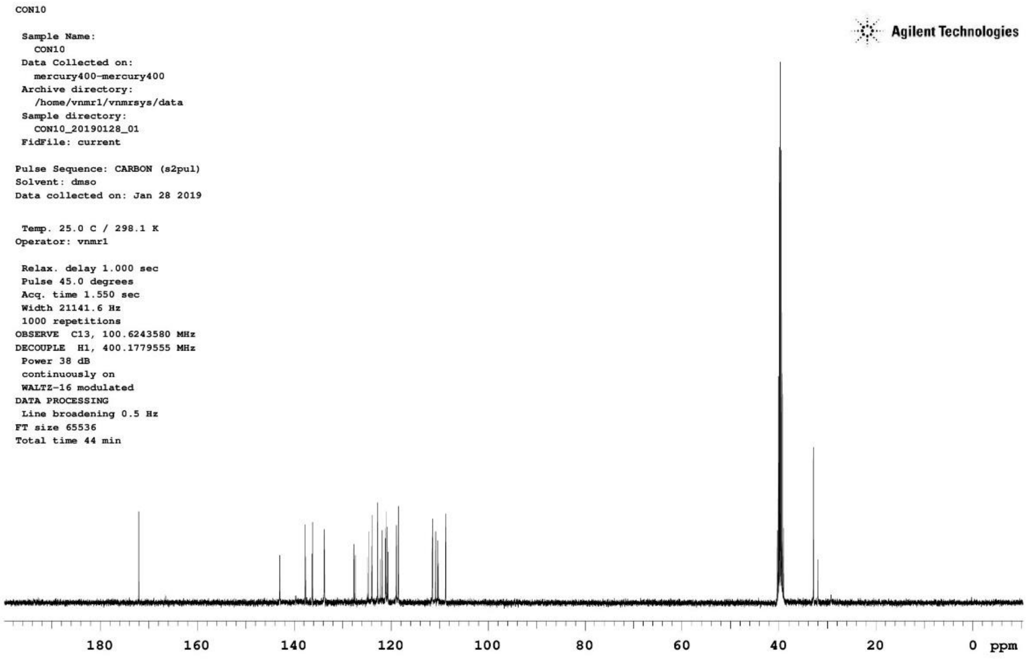

**Figure S36.** HRMS spectrum of compound **3i**



Data File: C:\LabSolutions\Data\Analiz\AOzdemir\CON-15_13.lcd

| Elmt | Val. | Min | Max | Elmt | Val. | Min | Max | Elmt | Val. | Min | Max | Elmt | Val. | Min | Max | Use Adduct |
|---|---|---|---|---|---|---|---|---|---|---|---|---|---|---|---|---|
| H | 1 | 0 | 30 | O | 2 | 0 | 4 | S | 2 | 0 | 2 | Ru | 2 | 0 | 0 | H |
| C | 4 | 15 | 25 | F | 1 | 0 | 0 | Cl | 1 | 0 | 1 | Pd | 2 | 0 | 0 | |
| N | 3 | 1 | 6 | P | 3 | 0 | 0 | Br | 1 | 0 | 0 | I | 3 | 0 | 0 | |

Error Margin (ppm): 5
HC Ratio: unlimited
Max Isotopes: 3
MSn Iso RI (%): 10.00

DBE Range: 10.0 - 20.0
Apply N Rule: yes
Isotope RI (%): 1.00
MSn Logic Mode: AND

Electron Ions: both
Use MSn Info: yes
Isotope Res: 9000
Max Results: 500

Event#: 1 MS(E+) Ret. Time : 6.920 Scan# : 1039

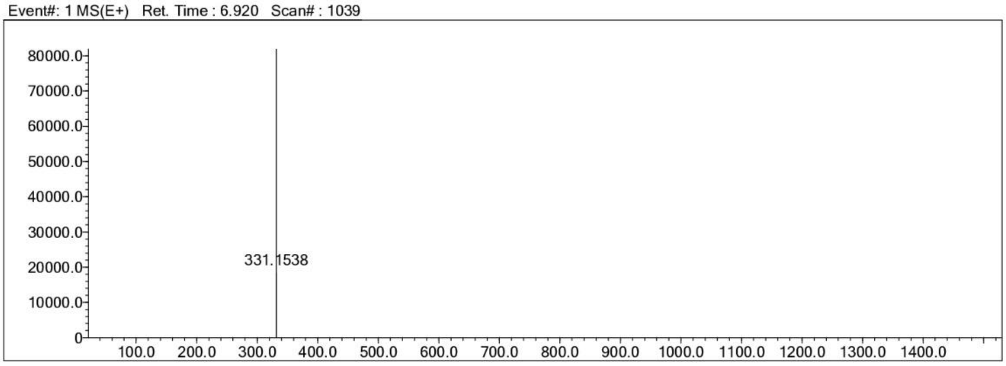


Measured region for 331.1538 m/z

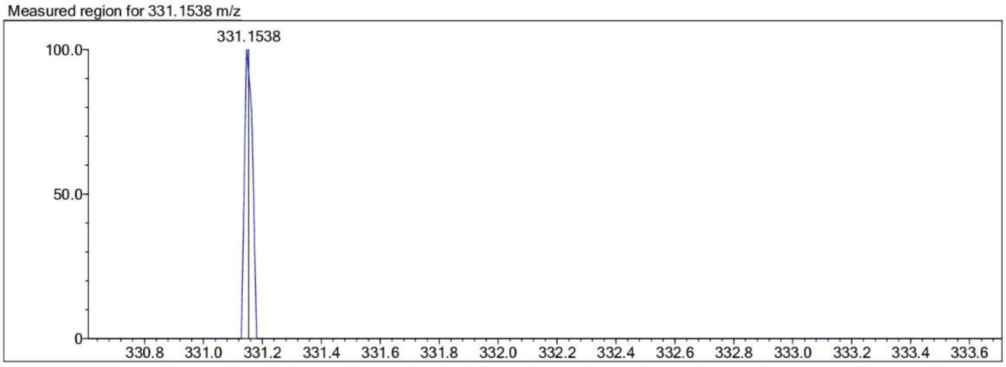


C20 H18 N4 O [M+H]+ : Predicted region for 331.1553 m/z

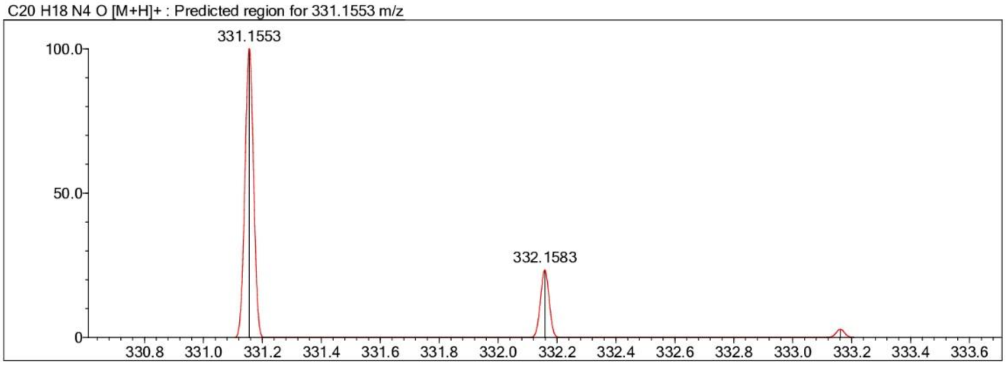


| Rank | Score | Formula (M) | Ion | Meas. m/z | Pred. m/z | Df. (mDa) | Df. (ppm) | Iso | DBE |
|---|---|---|---|---|---|---|---|---|---|
| 1 | 0.00 | C20 H18 N4 O | [M+H]+ | 331.1538 | 331.1553 | -1.5 | -4.53 | 0.00 | 14.0 |

**Figure S37.** IR spectrum of compound **3j**

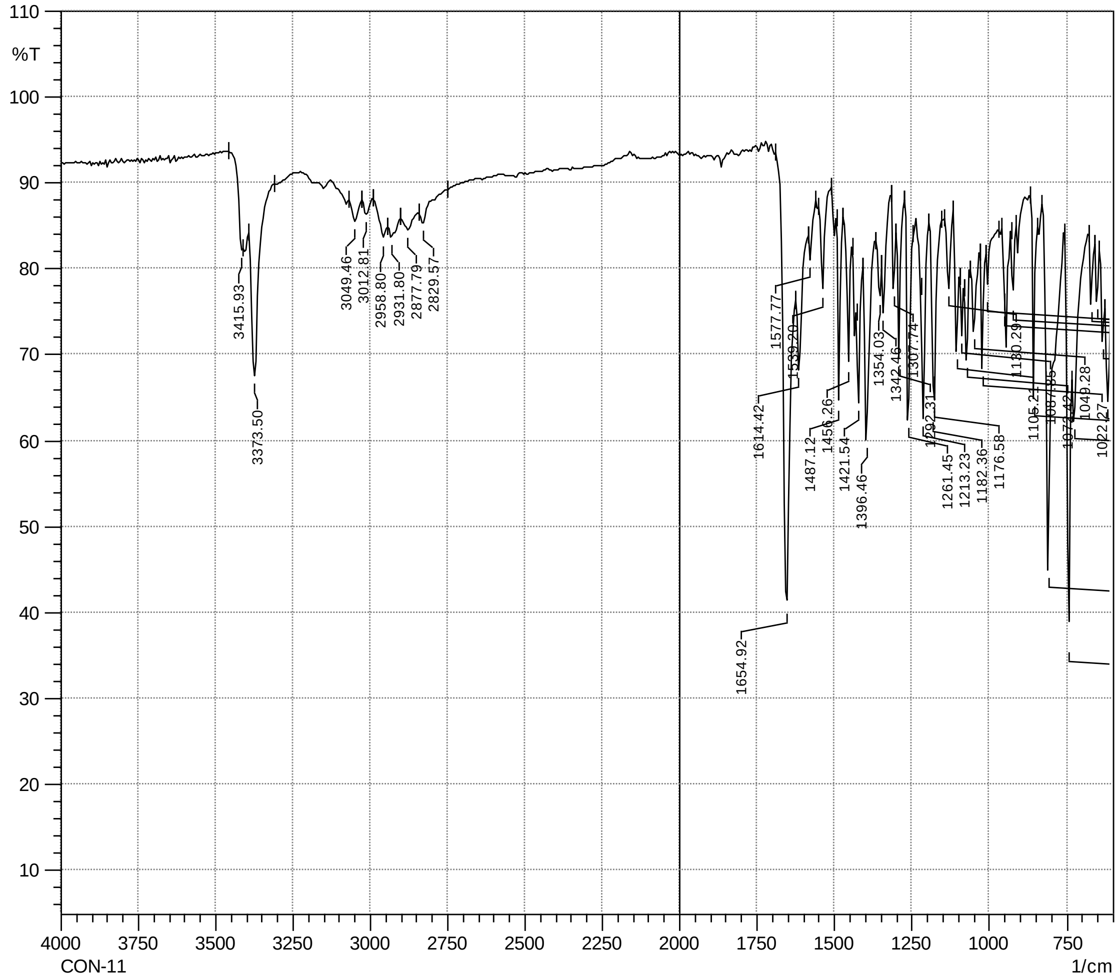

**Figure S38.** $^{1}$H NMR spectrum of compound **3j**

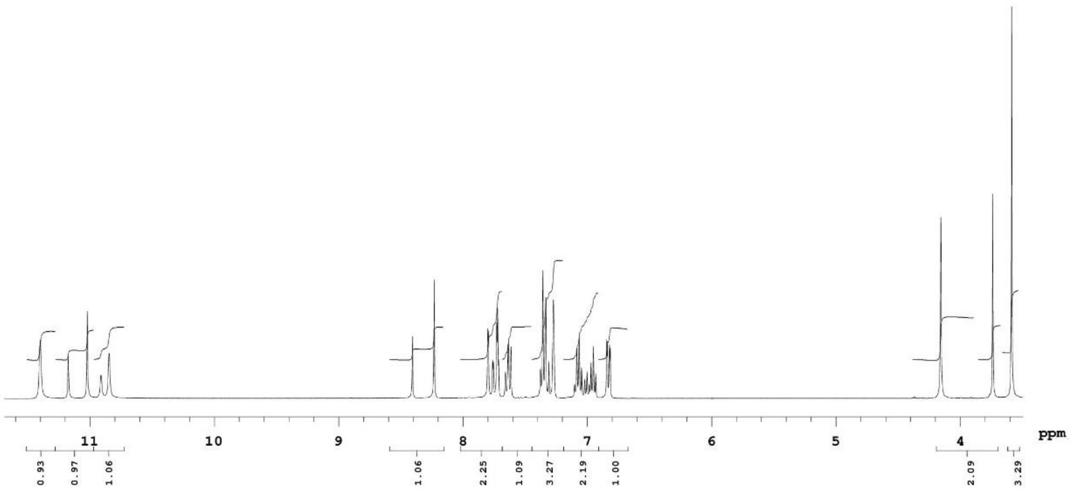


**Figure S39.** $^{13}$C NMR spectrum of compound **3j**

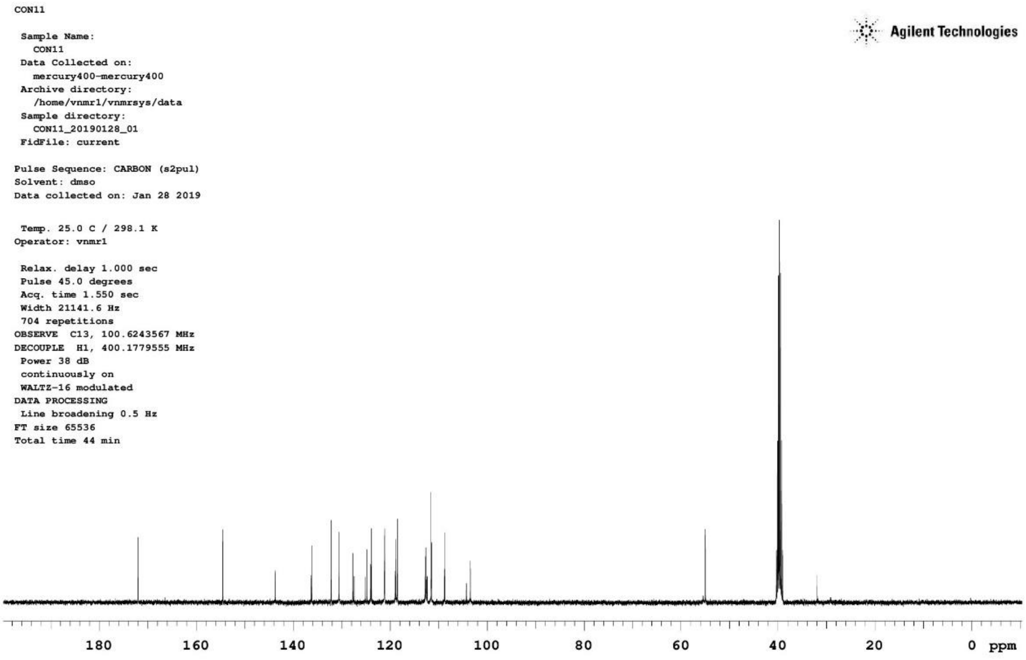

**Figure S40.** HRMS spectrum of compound **3j**



Data File: C:\LabSolutions\Data\Analiz\AOzdemir\CON-16_14.lcd

| Elmt | Val. | Min | Max | Elmt | Val. | Min | Max | Elmt | Val. | Min | Max | Elmt | Val. | Min | Max | Use Adduct |
|---|---|---|---|---|---|---|---|---|---|---|---|---|---|---|---|---|
| H | 1 | 0 | 30 | O | 2 | 1 | 4 | S | 2 | 0 | 2 | Ru | 2 | 0 | 0 | H |
| C | 4 | 15 | 25 | F | 1 | 0 | 0 | Cl | 1 | 0 | 1 | Pd | 2 | 0 | 0 | |
| N | 3 | 1 | 6 | P | 3 | 0 | 0 | Br | 1 | 0 | 0 | I | 3 | 0 | 0 | |

Error Margin (ppm): 5
HC Ratio: unlimited
Max Isotopes: 3
MSn Iso RI (%): 10.00

DBE Range: 10.0 - 20.0
Apply N Rule: yes
Isotope RI (%): 1.00
MSn Logic Mode: AND

Electron Ions: both
Use MSn Info: yes
Isotope Res: 9000
Max Results: 500

Event#: 1 MS(E+) Ret. Time : 6.227 -> 6.240 - 6.453 -> 6.680 Scan# : 935 -> 937 - 969 -> 1003

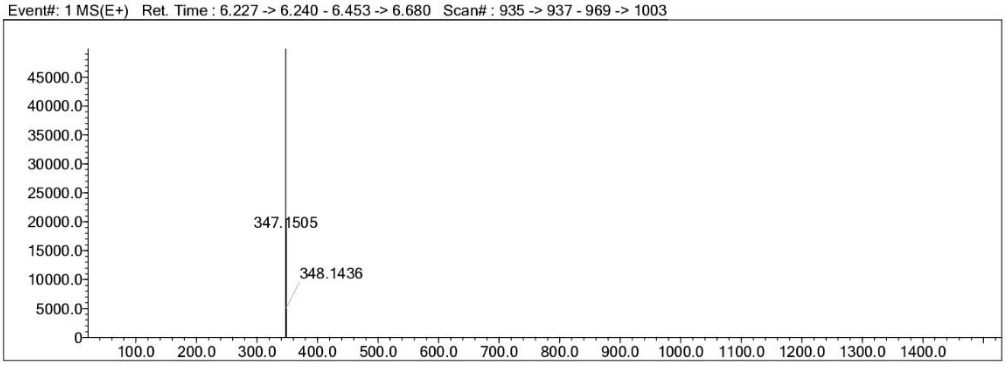


Measured region for 347.1505 m/z

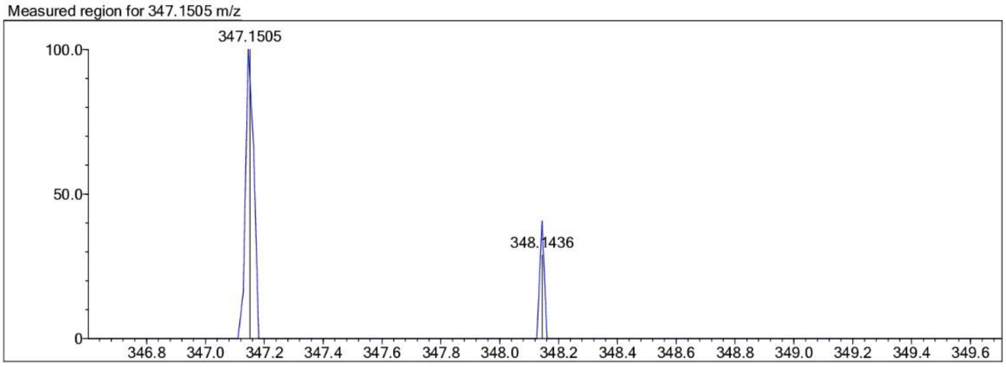


C20 H18 N4 O2 [M+H]+ : Predicted region for 347.1503 m/z

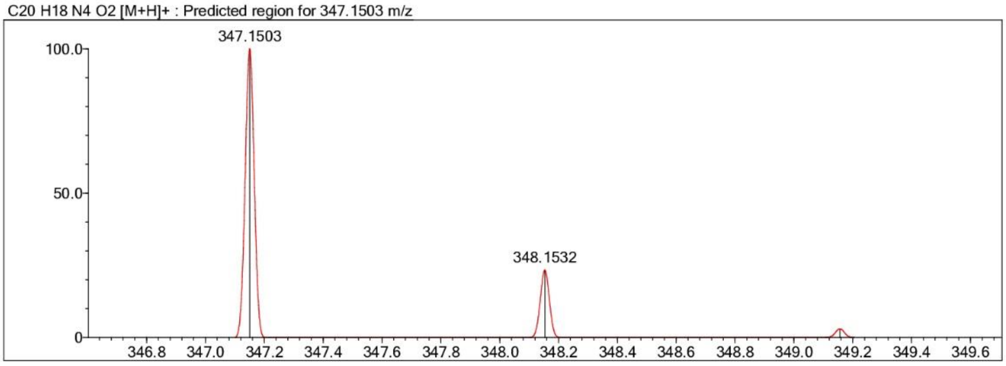


| Rank | Score | Formula (M) | Ion | Meas. m/z | Pred. m/z | Df. (mDa) | Df. (ppm) | Iso | DBE |
|---|---|---|---|---|---|---|---|---|---|
| 1 | 38.14 | C20 H18 N4 O2 | [M+H]+ | 347.1505 | 347.1503 | 0.2 | 0.58 | 38.14 | 14.0 |

**Figure S41.** IR spectrum of compound **4a**

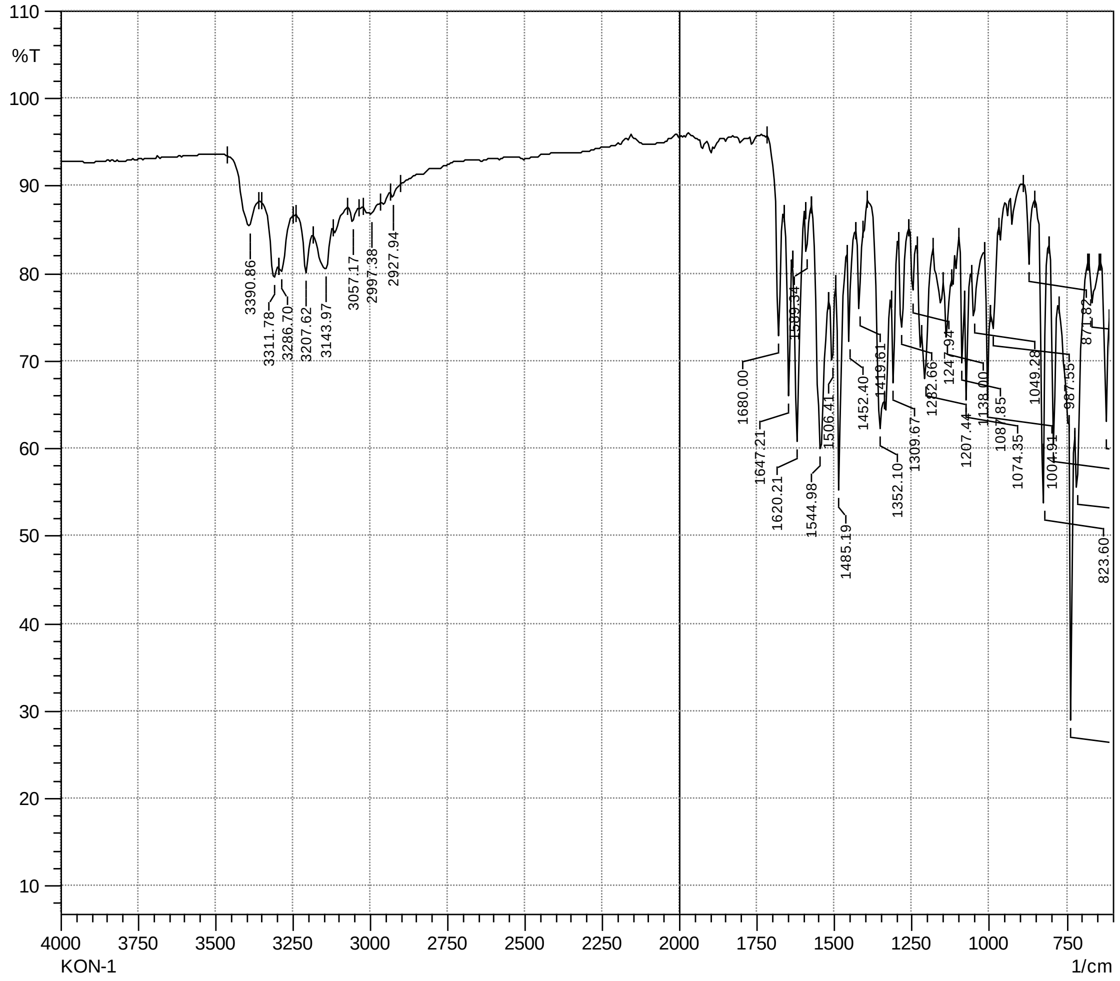

**Figure S42.** $^{1}$H NMR spectrum of compound **4a**

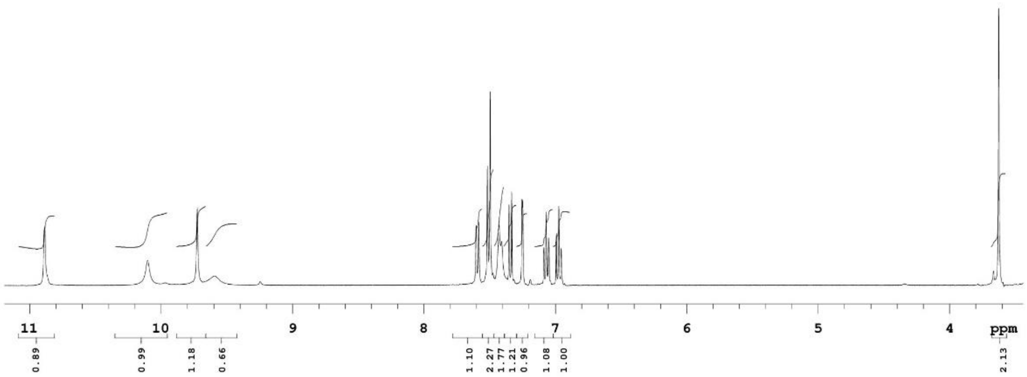


**Figure S43.** $^{13}$C NMR spectrum of compound **4a**

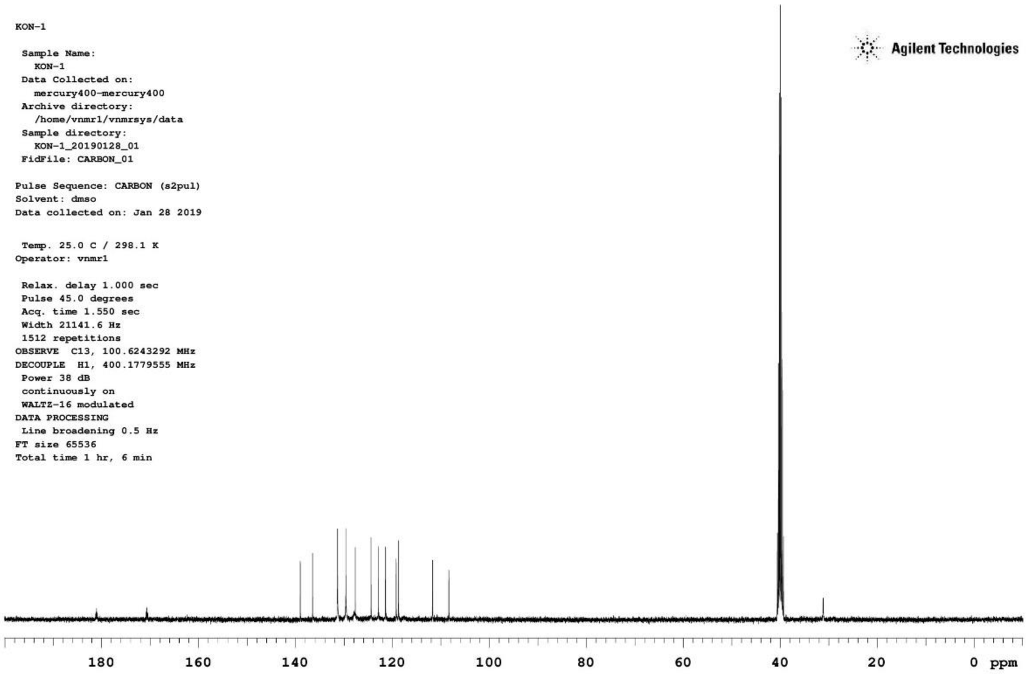

**Figure S44.** HRMS spectrum of compound **4a**

Formula Predictor Report - KON-1_17.lcd        Page 1 of 1

Data File: C:\LabSolutions\Data\Analiz\AOzdemir\KON-1_17.lcd

| Elmt | Val. | Min | Max | Elmt | Val. | Min | Max | Elmt | Val. | Min | Max | Elmt | Val. | Min | Max | Use Adduct |
|---|---|---|---|---|---|---|---|---|---|---|---|---|---|---|---|---|
| H | 1 | 0 | 30 | O | 2 | 0 | 5 | S | 2 | 0 | 1 | Ru | 2 | 0 | 0 | H |
| C | 4 | 15 | 25 | F | 1 | 0 | 0 | Cl | 1 | 0 | 0 | Pd | 2 | 0 | 0 | |
| N | 3 | 1 | 5 | P | 3 | 0 | 0 | Br | 1 | 0 | 1 | I | 3 | 0 | 0 | |

| | | | | | |
|---|---|---|---|---|---|
| Error Margin (ppm): | 5 | DBE Range: | 5.0 - 15.0 | Electron Ions: | both |
| HC Ratio: | unlimited | Apply N Rule: | yes | Use MSn Info: | yes |
| Max Isotopes: | 3 | Isotope RI (%): | 1.00 | Isotope Res: | 9000 |
| MSn Iso RI (%): | 10.00 | MSn Logic Mode: | AND | Max Results: | 500 |

Event#: 1 MS(E+)   Ret. Time : 6.893   Scan# : 1035

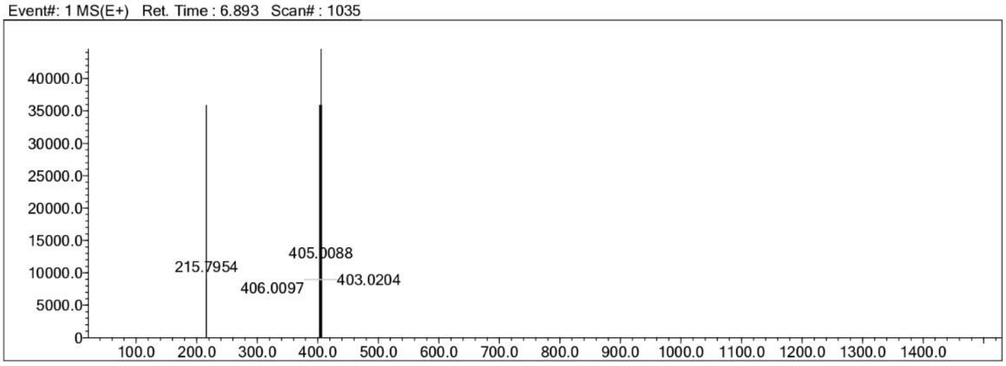


Measured region for 403.0204 m/z

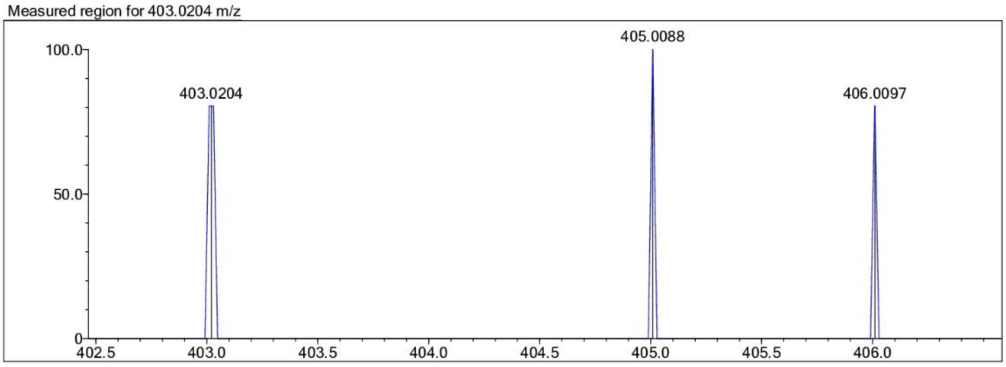


C17 H15 N4 O S Br [M+H]+ : Predicted region for 403.0223 m/z

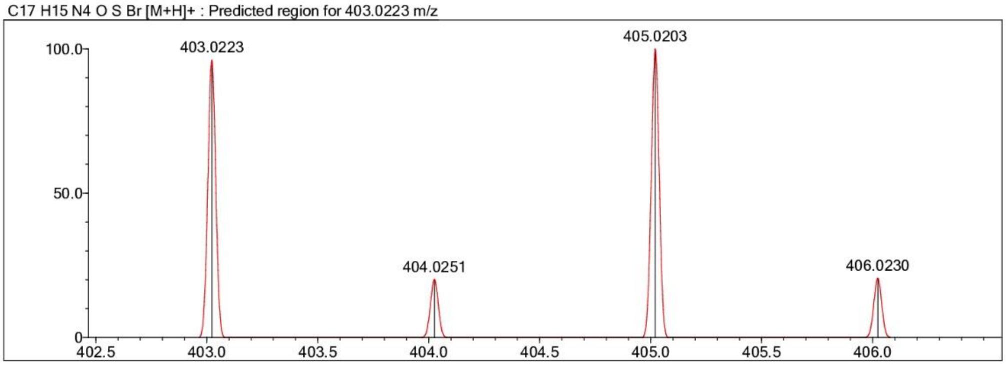


| Rank | Score | Formula (M) | Ion | Meas. m/z | Pred. m/z | Df. (mDa) | Df. (ppm) | Iso | DBE |
|---|---|---|---|---|---|---|---|---|---|
| 1 | 14.06 | C17 H15 N4 O S Br | [M+H]+ | 403.0204 | 403.0223 | -1.9 | -4.71 | 15.50 | 12.0 |

**Figure S45.** IR spectrum of compound **4b**

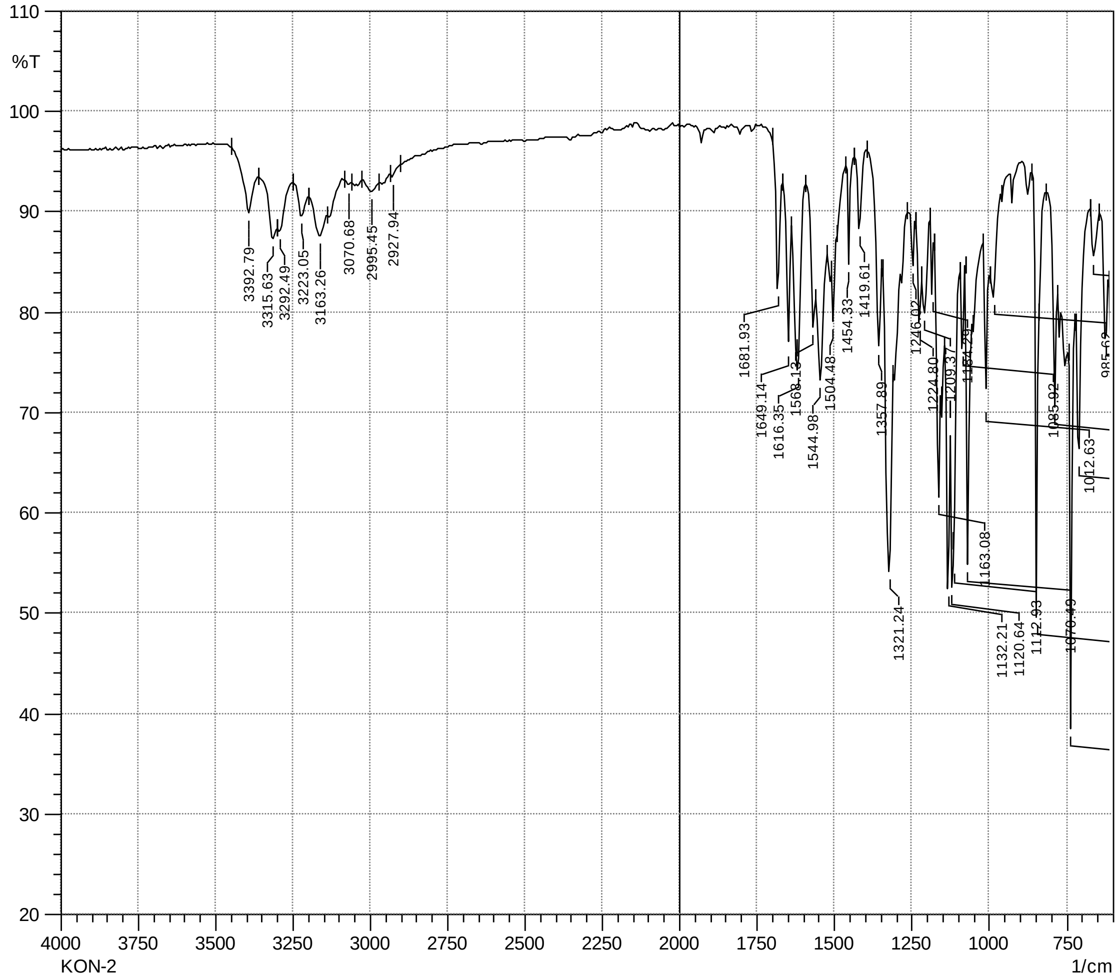

**Figure S46.** $^{1}$H NMR spectrum of compound **4b**

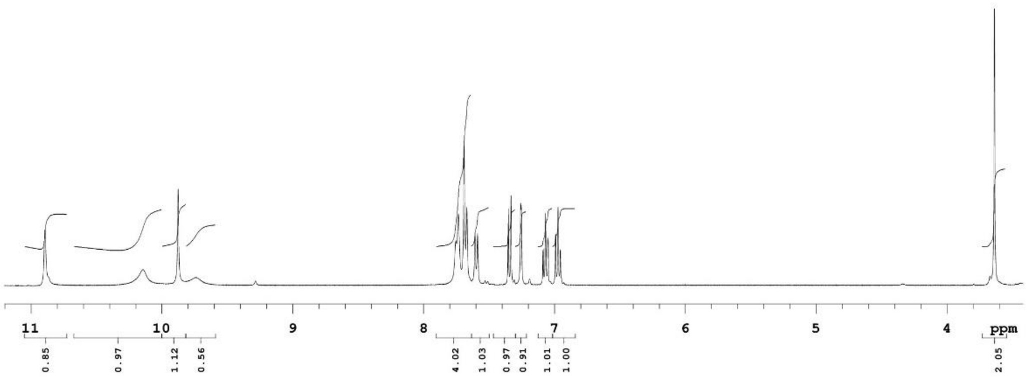


**Figure S47.** $^{13}$C NMR spectrum of compound **4b**

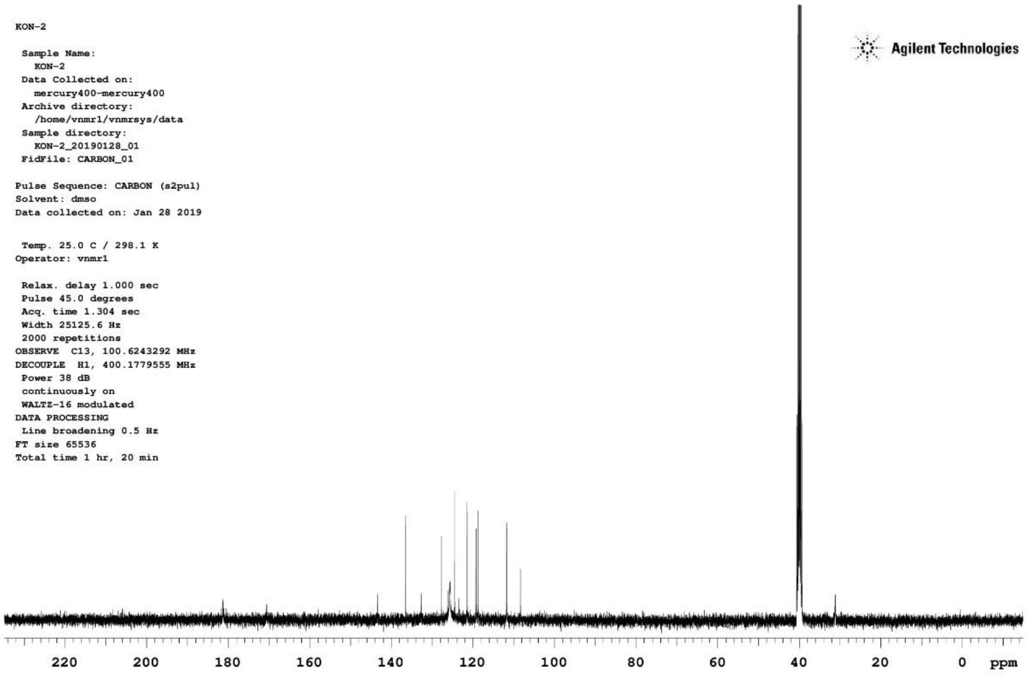

**Figure S48.** HRMS spectrum of compound **4b**

Formula Predictor Report - KON-2_18.lcd

Page 1 of 1

Data File: C:\LabSolutions\Data\Analiz\AOzdemir\KON-2_18.lcd

| Elmt | Val. | Min | Max | Elmt | Val. | Min | Max | Elmt | Val. | Min | Max | Elmt | Val. | Min | Max | Use Adduct |
|---|---|---|---|---|---|---|---|---|---|---|---|---|---|---|---|---|
| H | 1 | 0 | 30 | O | 2 | 0 | 4 | S | 2 | 0 | 1 | Ru | 2 | 0 | 0 | H |
| C | 4 | 15 | 25 | F | 1 | 0 | 3 | Cl | 1 | 0 | 0 | Pd | 2 | 0 | 0 | |
| N | 3 | 1 | 5 | P | 3 | 0 | 0 | Br | 1 | 0 | 0 | I | 3 | 0 | 0 | |

| | | | | | |
|---|---|---|---|---|---|
| Error Margin (ppm): | 5 | DBE Range: | 5.0 - 15.0 | Electron Ions: | both |
| HC Ratio: | unlimited | Apply N Rule: | yes | Use MSn Info: | yes |
| Max Isotopes: | 3 | Isotope RI (%): | 1.00 | Isotope Res: | 9000 |
| MSn Iso RI (%): | 10.00 | MSn Logic Mode: | AND | Max Results: | 500 |

Event#: 1 MS(E+) Ret. Time : 7.013 Scan# : 1053

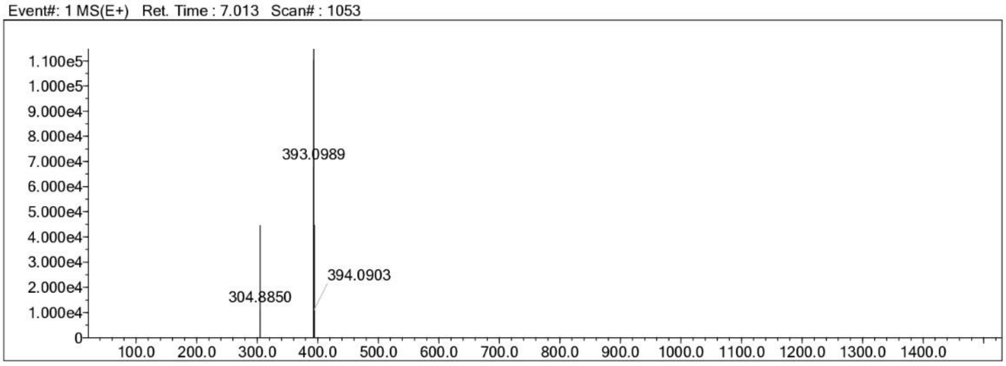


Measured region for 393.0989 m/z

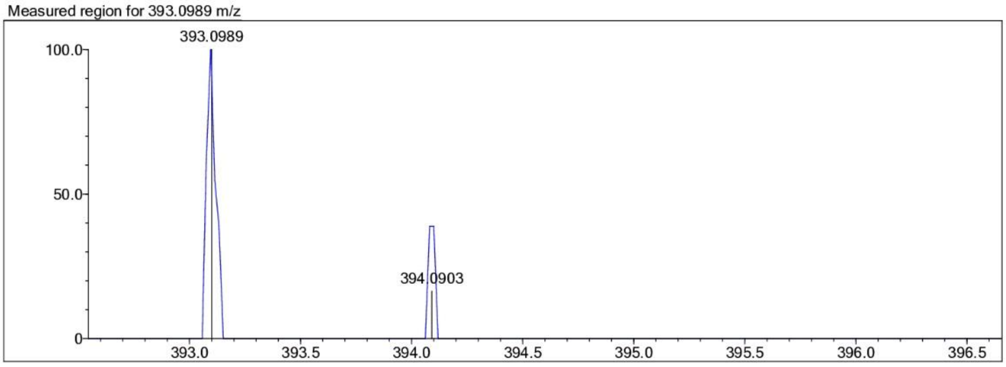


C18 H15 N4 O F3 S [M+H]+ : Predicted region for 393.0991 m/z

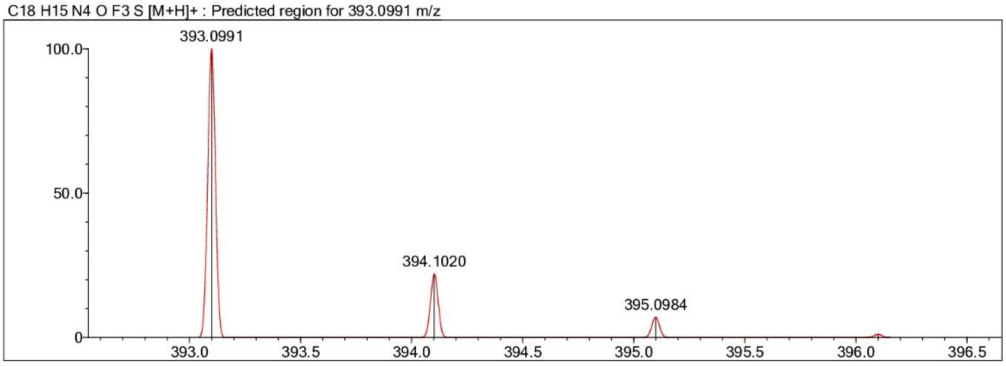


| Rank | Score | Formula (M) | Ion | Meas. m/z | Pred. m/z | Df. (mDa) | Df. (ppm) | Iso | DBE |
|---|---|---|---|---|---|---|---|---|---|
| 1 | 0.00 | C18 H15 N4 O F3 S | [M+H]+ | 393.0989 | 393.0991 | -0.2 | -0.51 | 0.00 | 12.0 |

**Figure S49.** IR spectrum of compound **4c**

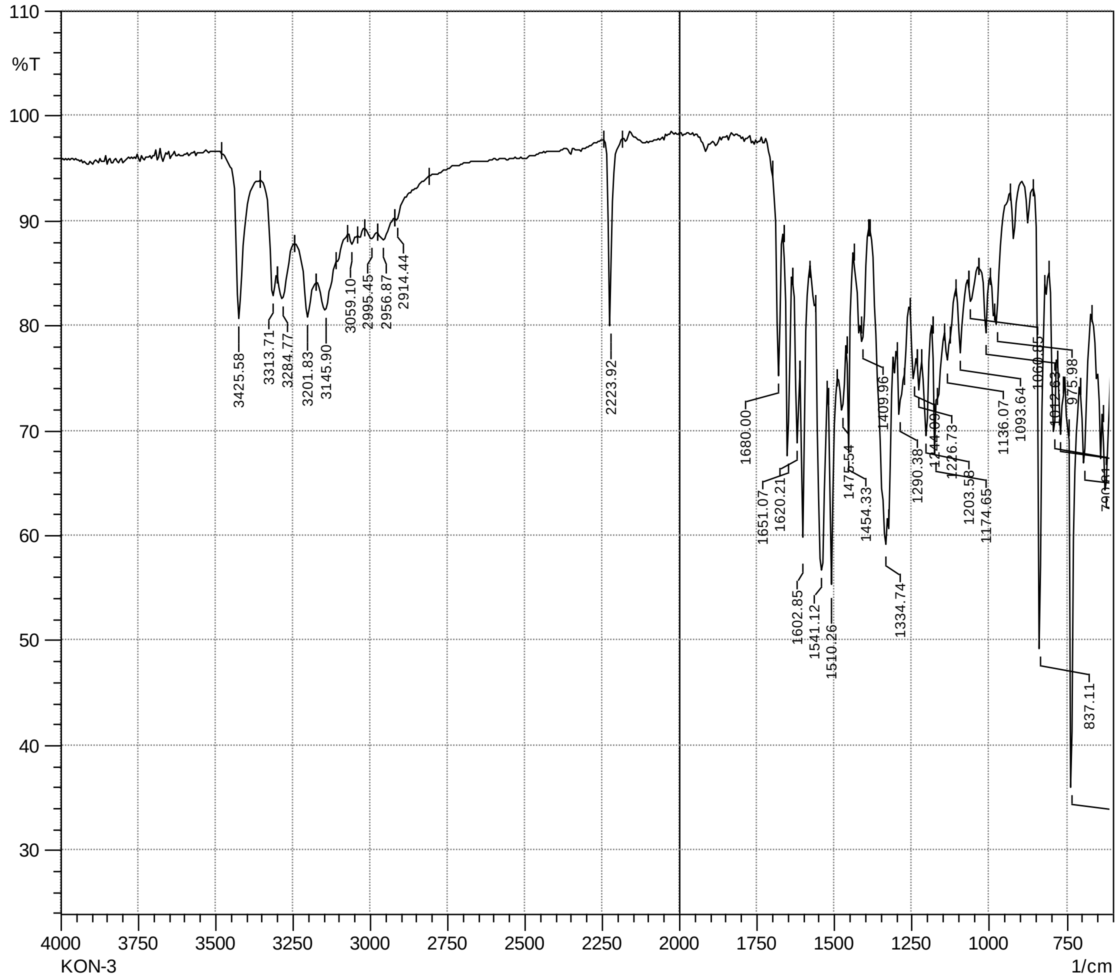

**Figure S50.** $^{1}$H NMR spectrum of compound **4c**

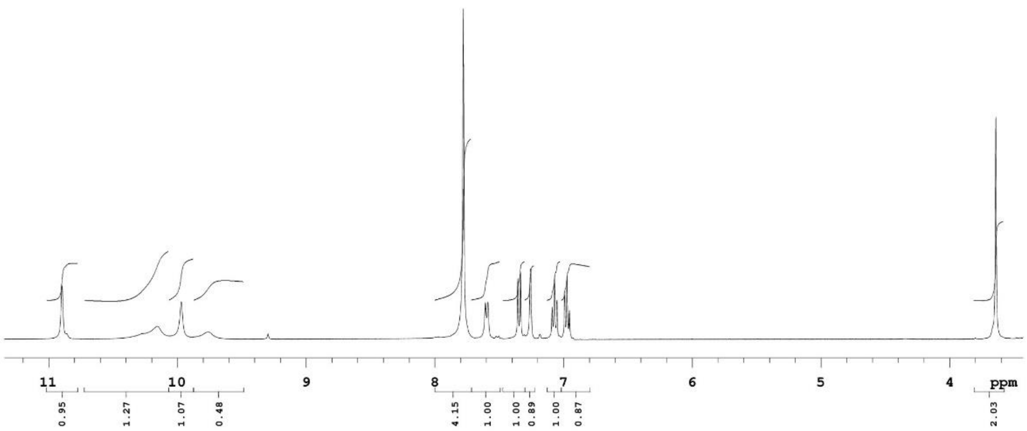


**Figure S51.** $^{13}$C NMR spectrum of compound **4c**

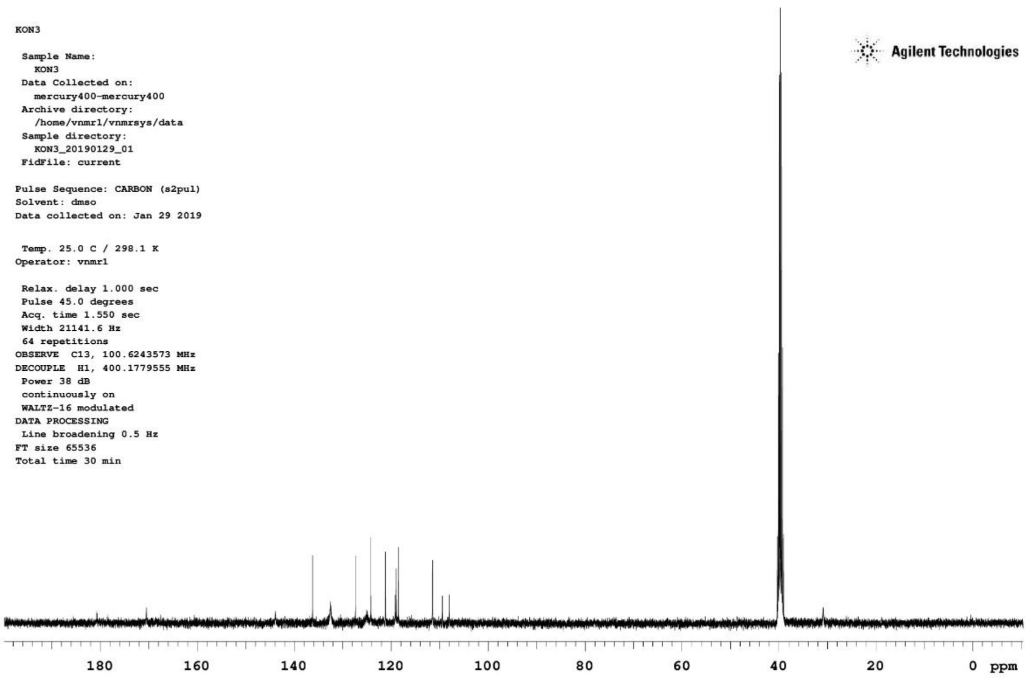

**Figure S52.** HRMS spectrum of compound **4c**

Formula Predictor Report - KON-3_19.lcd

Page 1 of 1

Data File: C:\LabSolutions\Data\Analiz\AOzdemir\KON-3_19.lcd

| Elmt | Val. | Min | Max | Elmt | Val. | Min | Max | Elmt | Val. | Min | Max | Elmt | Val. | Min | Max | Use Adduct |
|---|---|---|---|---|---|---|---|---|---|---|---|---|---|---|---|---|
| H | 1 | 0 | 30 | O | 2 | 0 | 4 | S | 2 | 0 | 1 | Ru | 2 | 0 | 0 | H |
| C | 4 | 15 | 25 | F | 1 | 0 | 0 | Cl | 1 | 0 | 0 | Pd | 2 | 0 | 0 | |
| N | 3 | 1 | 5 | P | 3 | 0 | 0 | Br | 1 | 0 | 0 | I | 3 | 0 | 0 | |

Error Margin (ppm): 5
HC Ratio: unlimited
Max Isotopes: 3
MSn Iso RI (%): 10.00

DBE Range: 5.0 - 15.0
Apply N Rule: yes
Isotope RI (%): 1.00
MSn Logic Mode: AND

Electron Ions: both
Use MSn Info: yes
Isotope Res: 9000
Max Results: 500

Event#: 1 MS(E+) Ret. Time : 6.320 Scan# : 949

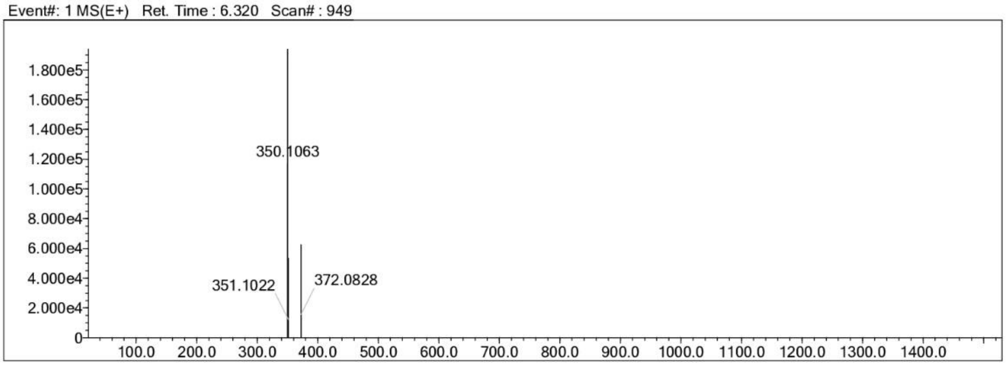


Measured region for 350.1063 m/z

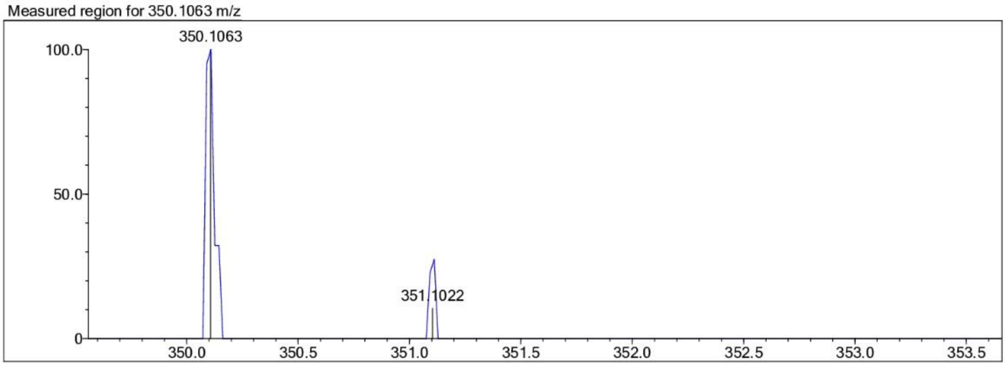


C18 H15 N5 O S [M+H]+ : Predicted region for 350.1070 m/z

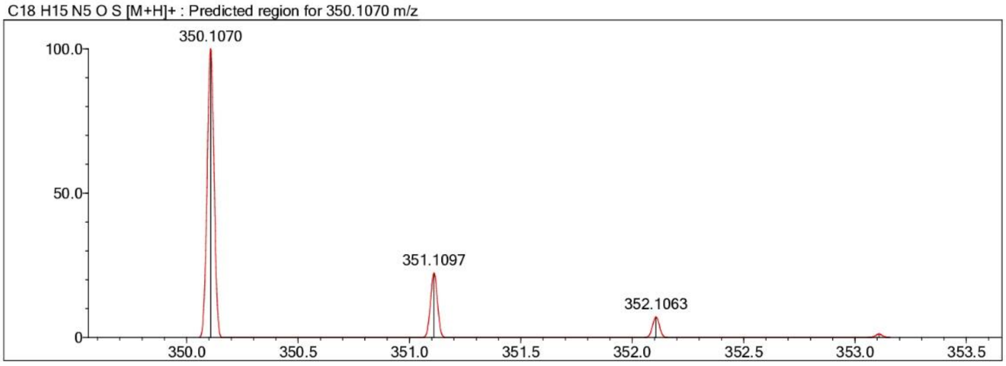


| Rank | Score | Formula (M) | Ion | Meas. m/z | Pred. m/z | Df. (mDa) | Df. (ppm) | Iso | DBE |
|---|---|---|---|---|---|---|---|---|---|
| 1 | 0.00 | C18 H15 N5 O S | [M+H]+ | 350.1063 | 350.1070 | -0.7 | -2.00 | 0.00 | 14.0 |

**Figure S53.** IR spectrum of compound **4d**

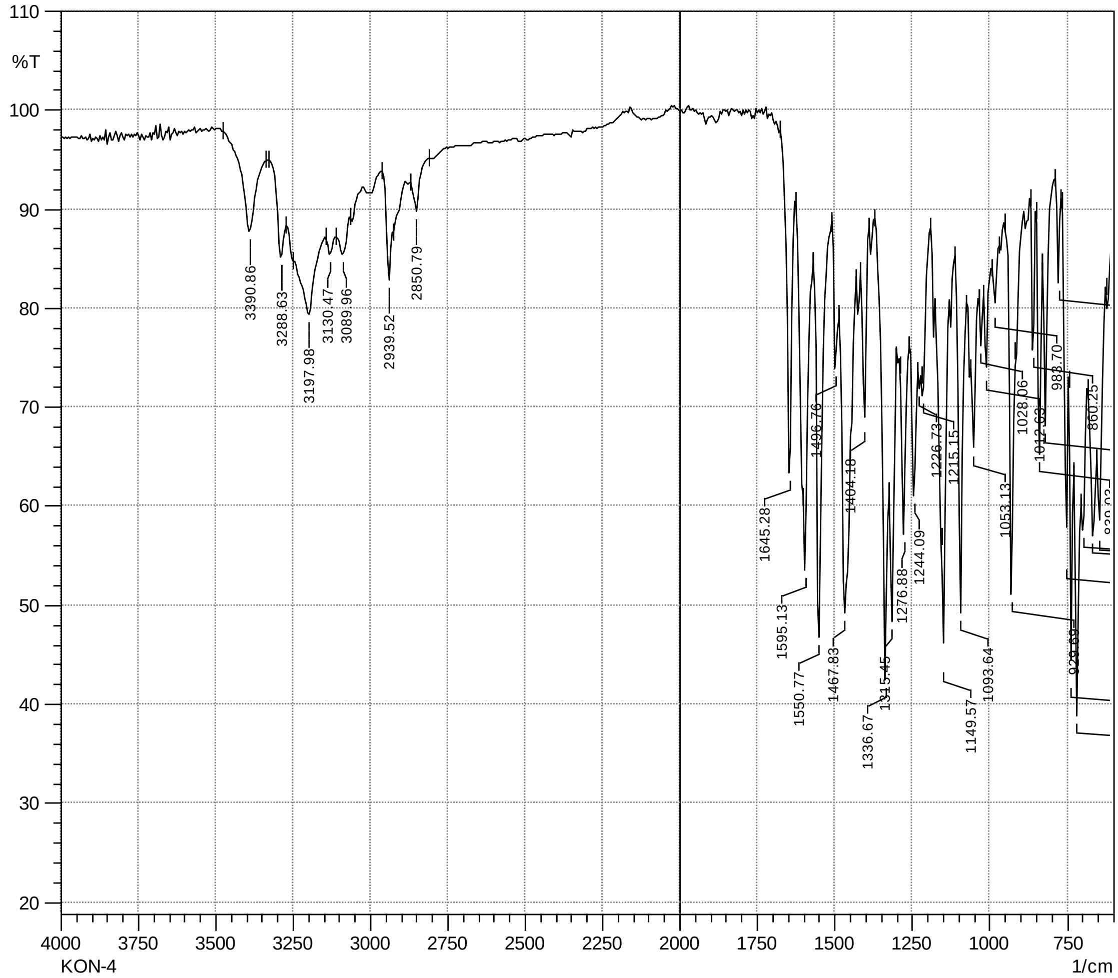

**Figure S54.** $^{1}$H NMR spectrum of compound **4d**

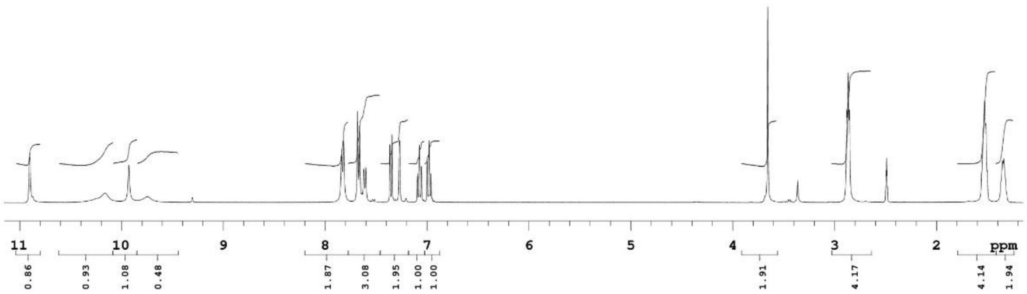


**Figure S55.** $^{13}$C NMR spectrum of compound **4d**

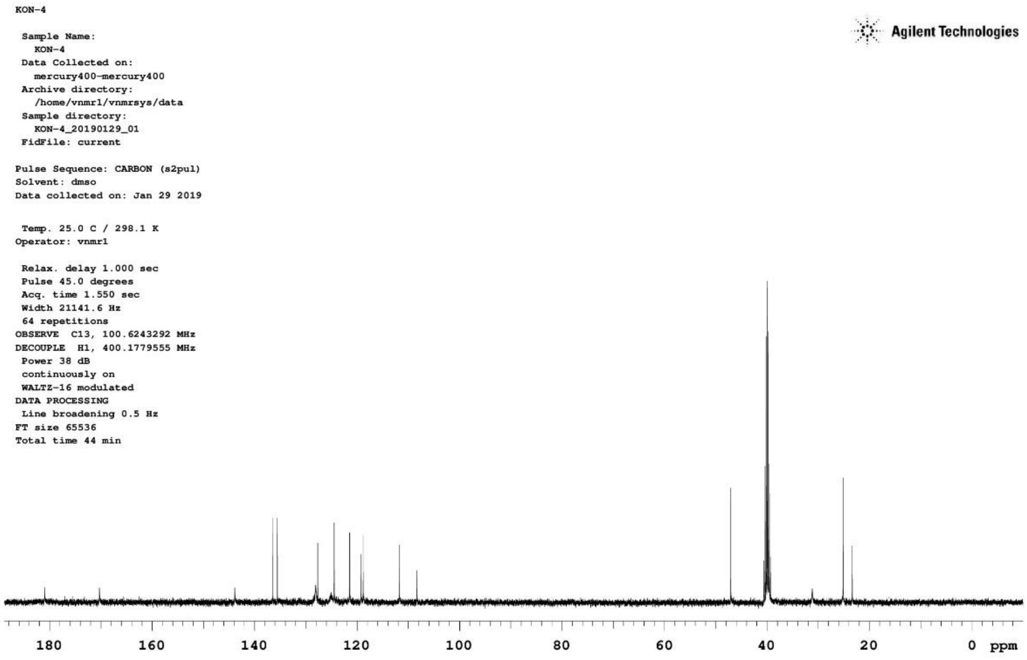

**Figure S56.** HRMS spectrum of compound **4d**

Formula Predictor Report - KON-4_20.lcd

Page 1 of 1

Data File: C:\LabSolutions\Data\Analiz\AOzdemir\KON-4_20.lcd

| Elmt | Val. | Min | Max | Elmt | Val. | Min | Max | Elmt | Val. | Min | Max | Elmt | Val. | Min | Max | Use Adduct |
|---|---|---|---|---|---|---|---|---|---|---|---|---|---|---|---|---|
| H | 1 | 0 | 30 | O | 2 | 0 | 4 | S | 2 | 0 | 2 | Ru | 2 | 0 | 0 | H |
| C | 4 | 15 | 25 | F | 1 | 0 | 0 | Cl | 1 | 0 | 0 | Pd | 2 | 0 | 0 | |
| N | 3 | 1 | 5 | P | 3 | 0 | 0 | Br | 1 | 0 | 0 | I | 3 | 0 | 0 | |

| | | | | | |
|---|---|---|---|---|---|
| Error Margin (ppm): | 5 | DBE Range: | 5.0 - 15.0 | Electron Ions: | both |
| HC Ratio: | unlimited | Apply N Rule: | yes | Use MSn Info: | yes |
| Max Isotopes: | 3 | Isotope RI (%): | 1.00 | Isotope Res: | 9000 |
| MSn Iso RI (%): | 10.00 | MSn Logic Mode: | AND | Max Results: | 500 |

Event#: 1 MS(E+) Ret. Time : 7.000 Scan# : 1051

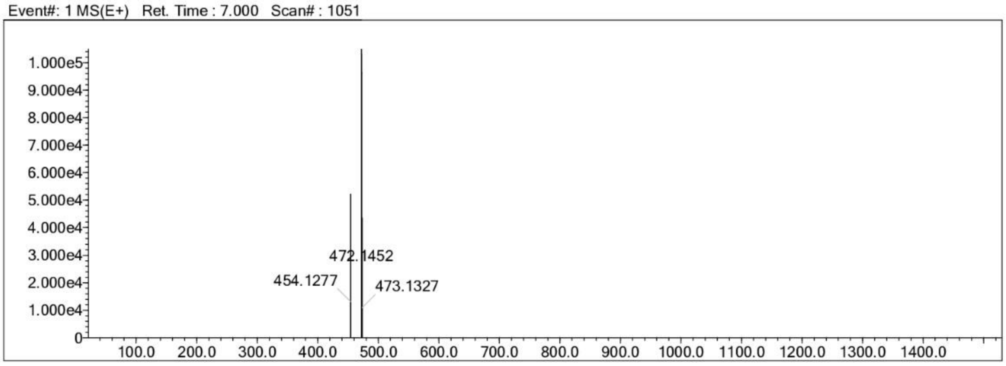


Measured region for 472.1452 m/z

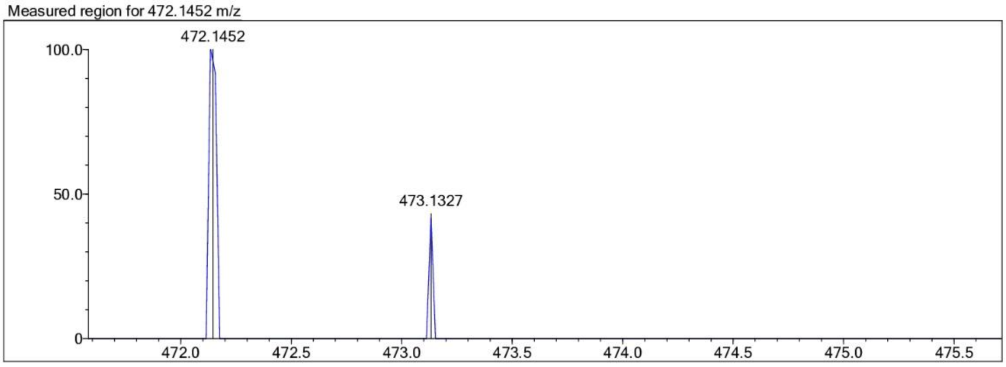


C22 H25 N5 O3 S2 [M+H]+ : Predicted region for 472.1472 m/z

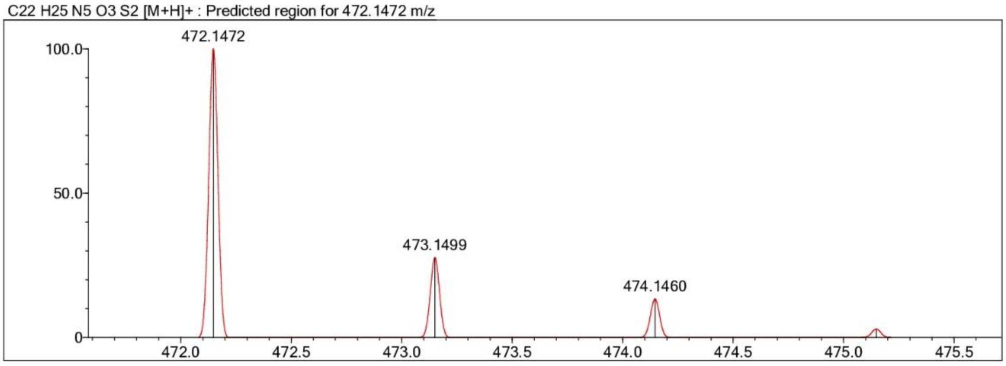


| Rank | Score | Formula (M) | Ion | Meas. m/z | Pred. m/z | Df. (mDa) | Df. (ppm) | Iso | DBE |
|---|---|---|---|---|---|---|---|---|---|
| 1 | 0.00 | C22 H25 N5 O3 S2 | [M+H]+ | 472.1452 | 472.1472 | -2.0 | -4.24 | 0.00 | 13.0 |

**Figure S57.** IR spectrum of compound **4e**

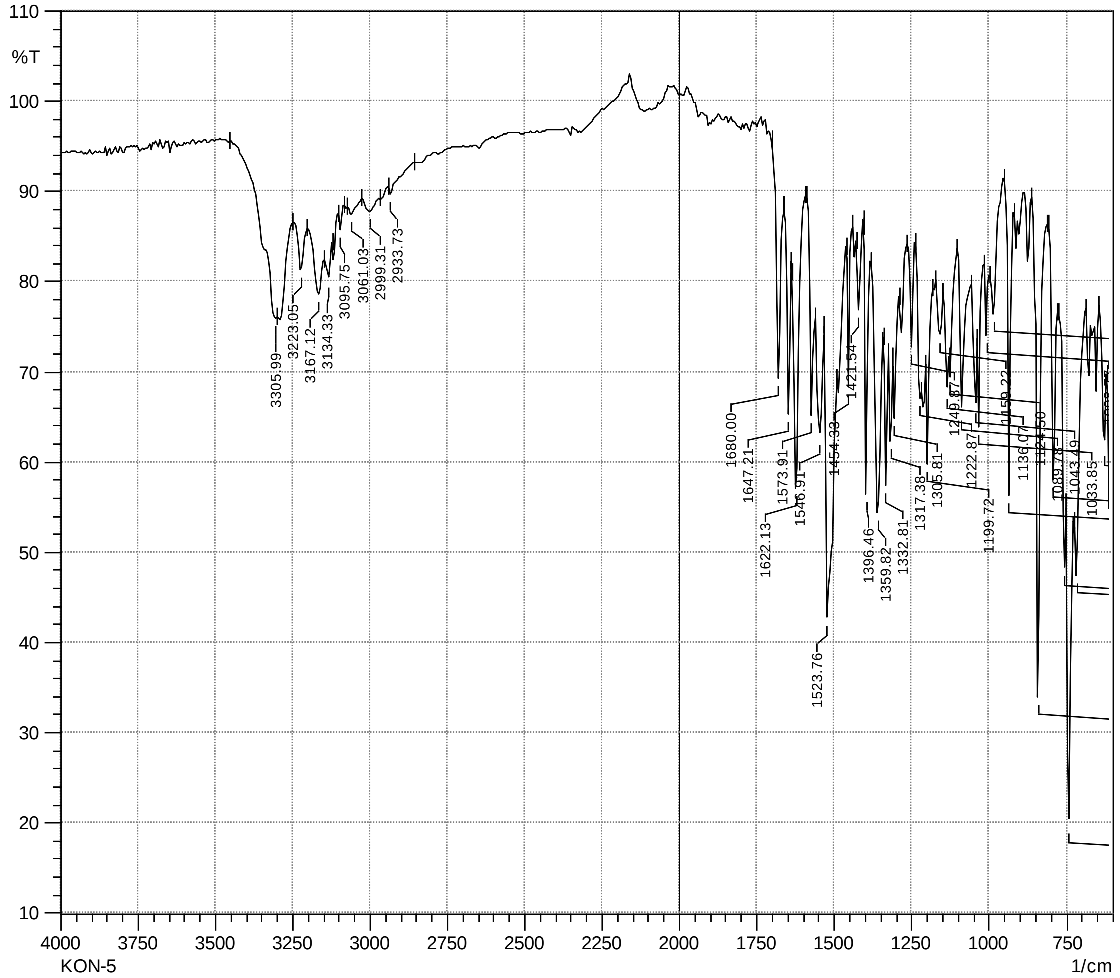

**Figure S58.** $^{1}$H NMR spectrum of compound **4e**

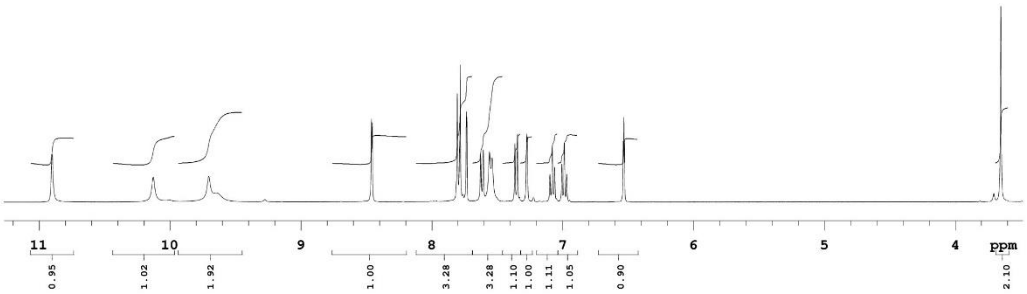


**Figure S59.** $^{13}$C NMR spectrum of compound **4e**

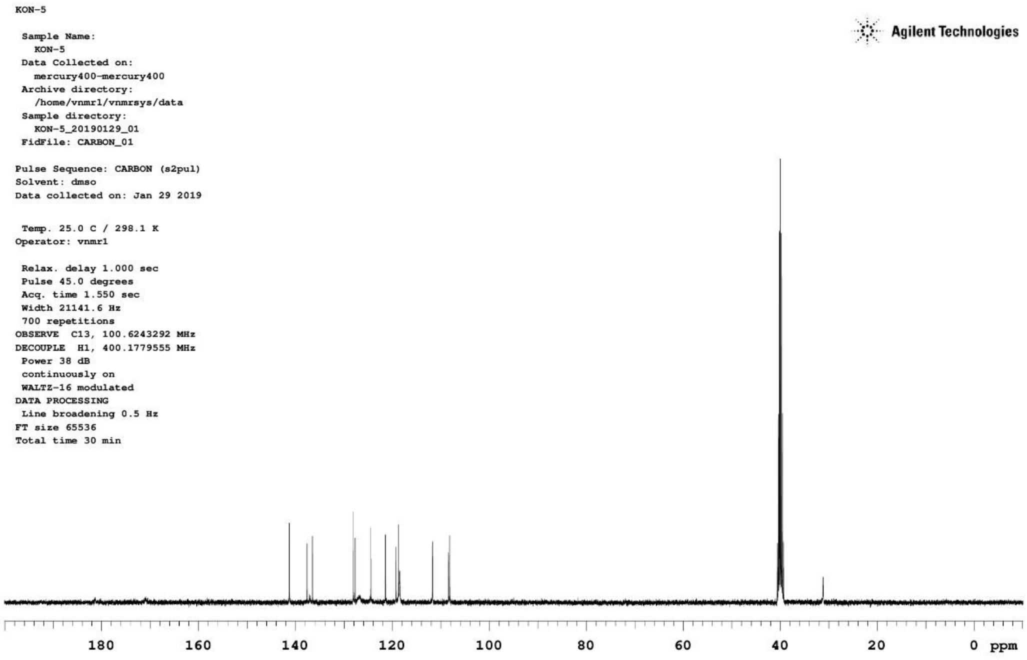

**Figure S60.** HRMS spectrum of compound **4e**



Data File: C:\LabSolutions\Data\Analiz\mdaltintop\KON-5_2.lcd

| Elmt | Val. | Min | Max | Elmt | Val. | Min | Max | Elmt | Val. | Min | Max | Elmt | Val. | Min | Max | Use Adduct |
|---|---|---|---|---|---|---|---|---|---|---|---|---|---|---|---|---|
| H | 1 | 10 | 40 | O | 2 | 1 | 2 | S | 2 | 0 | 1 | Ru | 2 | 0 | 0 | H |
| C | 4 | 10 | 40 | F | 1 | 0 | 0 | Cl | 1 | 0 | 0 | Pd | 2 | 0 | 0 | |
| N | 3 | 2 | 6 | P | 3 | 0 | 0 | Br | 1 | 0 | 0 | I | 3 | 0 | 0 | |

| | | | | | |
|---|---|---|---|---|---|
| Error Margin (ppm): | 5 | DBE Range: | 5.0 - 25.0 | Electron Ions: | both |
| HC Ratio: | unlimited | Apply N Rule: | yes | Use MSn Info: | yes |
| Max Isotopes: | 3 | Isotope RI (%): | 1.00 | Isotope Res: | 9000 |
| MSn Iso RI (%): | 10.00 | MSn Logic Mode: | AND | Max Results: | 200 |

Event#: 1 MS(E+) Ret. Time : 2.133 Scan# : 321

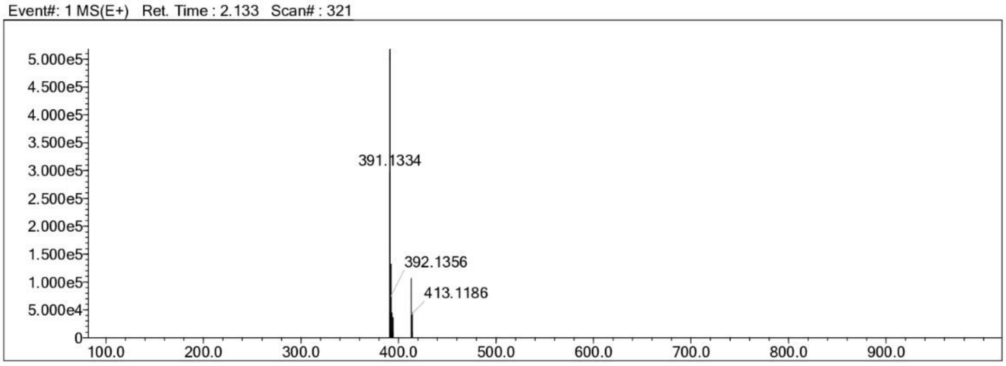


Measured region for 391.1334 m/z

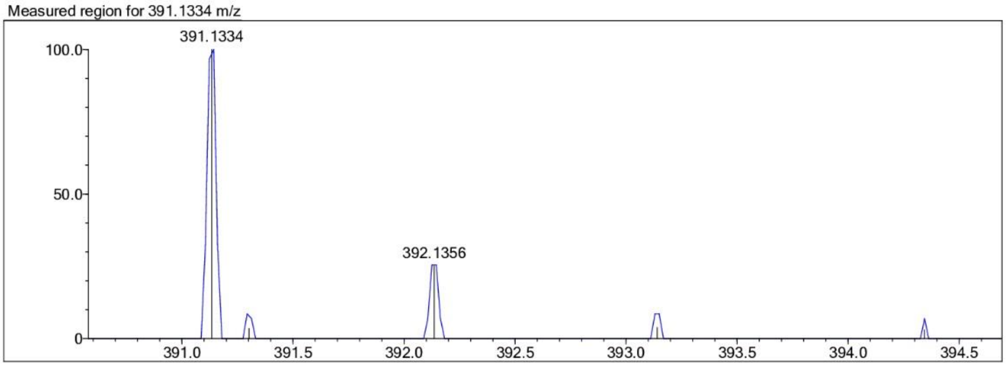


C20 H18 N6 O S [M+H]+ : Predicted region for 391.1336 m/z

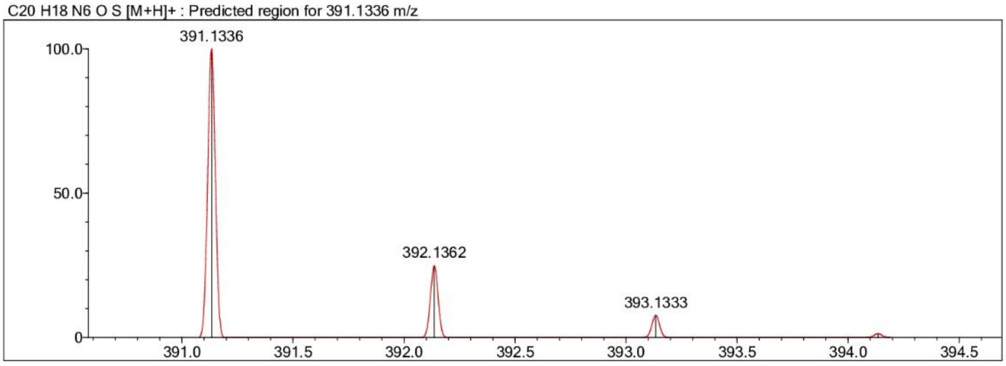


| Rank | Score | Formula (M) | Ion | Meas. m/z | Pred. m/z | Df. (mDa) | Df. (ppm) | Iso | DBE |
|---|---|---|---|---|---|---|---|---|---|
| 1 | 88.29 | C20 H18 N6 O S | [M+H]+ | 391.1334 | 391.1336 | -0.2 | -0.51 | 88.29 | 15.0 |

**Figure S61.** IR spectrum of compound **4f**

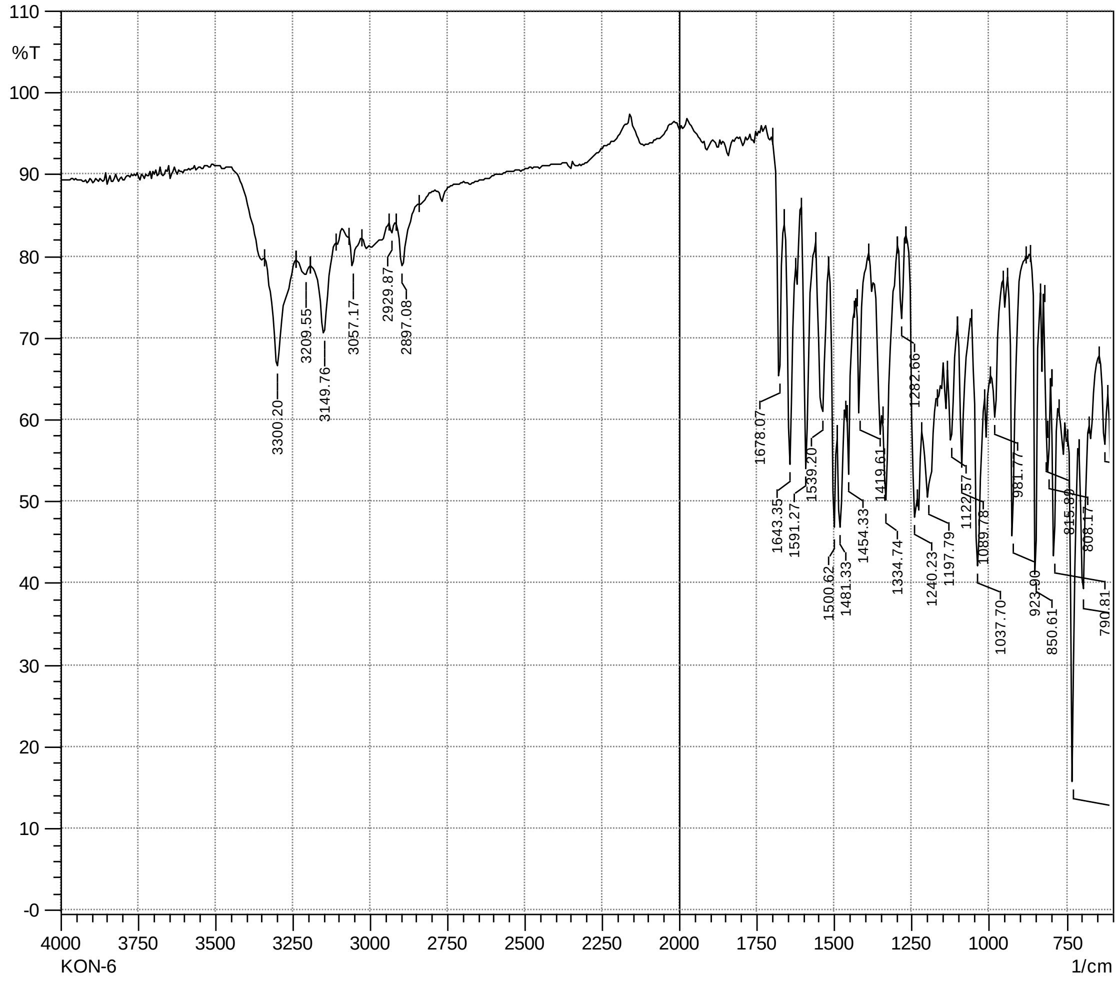

**Figure S62.** $^{1}$H NMR spectrum of compound **4f**

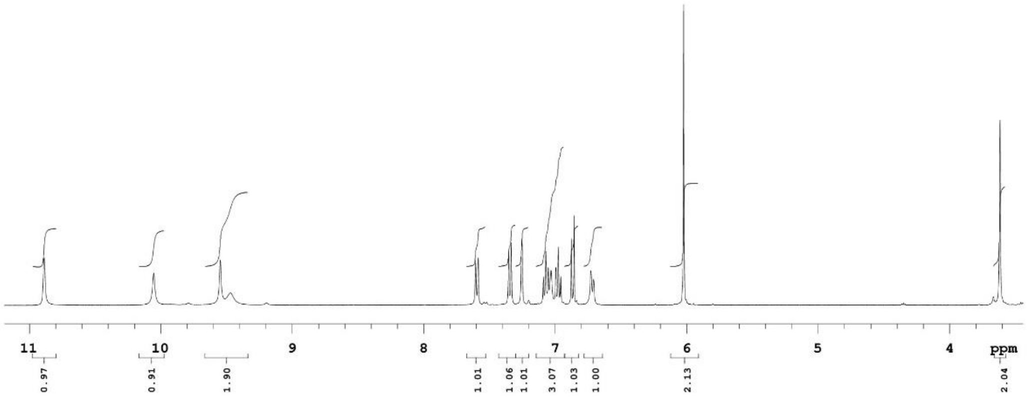


**Figure S63.** $^{13}$C NMR spectrum of compound **4f**

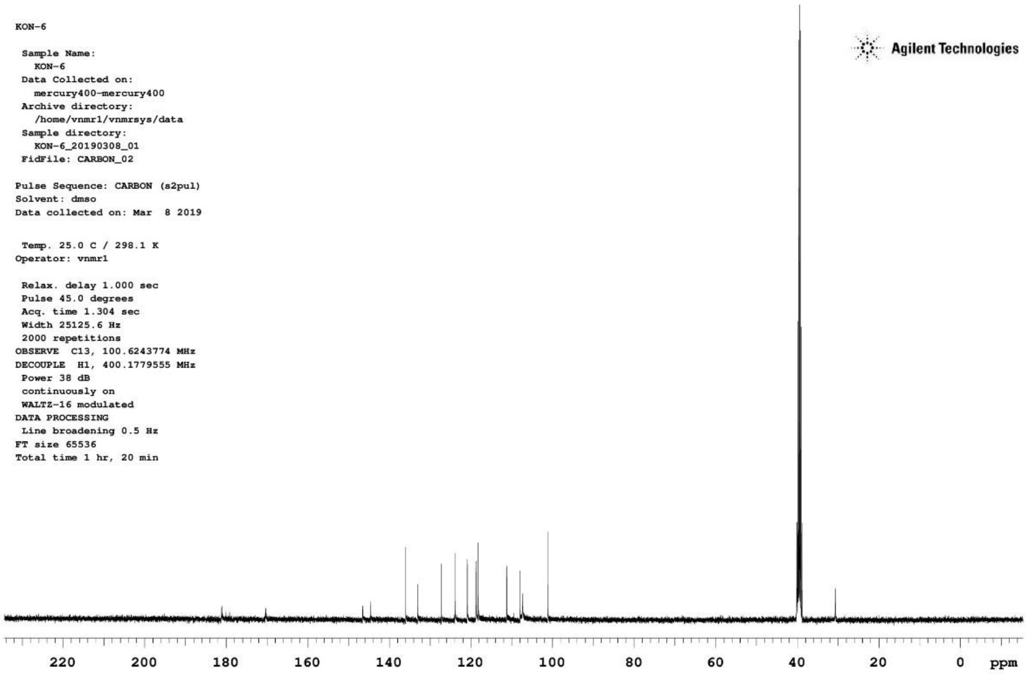

**Figure S64.** HRMS spectrum of compound **4f**

Formula Predictor Report - KON-6_58.lcd  Page 1 of 1

Data File: C:\LabSolutions\Data\Analiz\AOzdemir\KON-6_58.lcd

| Elmt | Val. | Min | Max | Elmt | Val. | Min | Max | Elmt | Val. | Min | Max | Elmt | Val. | Min | Max | Use Adduct |
|---|---|---|---|---|---|---|---|---|---|---|---|---|---|---|---|---|
| H | 1 | 10 | 40 | O | 2 | 1 | 5 | S | 2 | 1 | 2 | Ru | 2 | 0 | 0 | H |
| C | 4 | 18 | 30 | F | 1 | 0 | 0 | Cl | 1 | 0 | 0 | Pd | 2 | 0 | 0 | |
| N | 3 | 4 | 7 | P | 3 | 0 | 0 | Br | 1 | 0 | 1 | I | 3 | 0 | 0 | |

Error Margin (ppm): 20
HC Ratio: unlimited
Max Isotopes: 3
MSn Iso RI (%): 10.00

DBE Range: 10.0 - 21.0
Apply N Rule: yes
Isotope RI (%): 1.00
MSn Logic Mode: AND

Electron Ions: both
Use MSn Info: yes
Isotope Res: 9000
Max Results: 500

Event#: 1 MS(E+) Ret. Time : 2.200 -> 2.453 Scan# : 331 -> 369

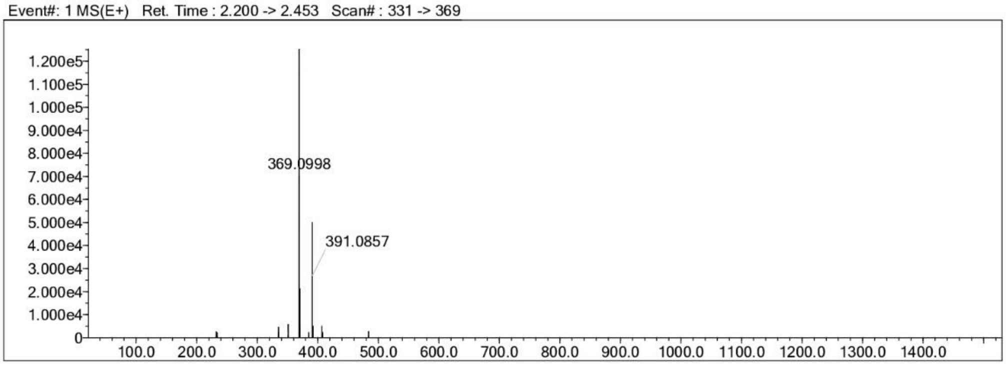


Measured region for 369.0998 m/z

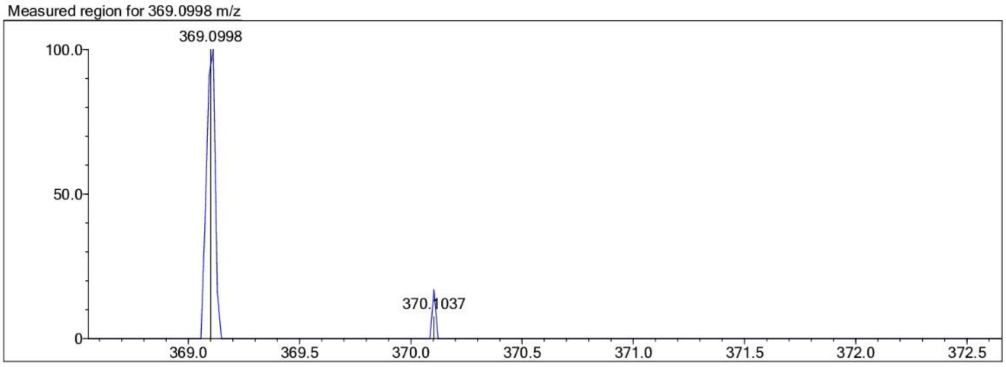


C18 H16 N4 O3 S [M+H]+ : Predicted region for 369.1016 m/z

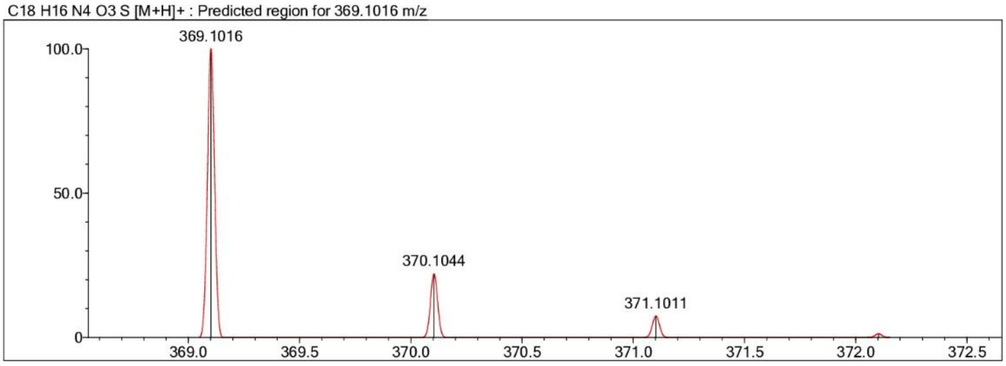


| Rank | Score | Formula (M) | Ion | Meas. m/z | Pred. m/z | Df. (mDa) | Df. (ppm) | Iso | DBE |
|---|---|---|---|---|---|---|---|---|---|
| 1 | 0.00 | C18 H16 N4 O3 S | [M+H]+ | 369.0998 | 369.1016 | -1.8 | -4.88 | 0.00 | 13.0 |

**Figure S65.** IR spectrum of compound **4g**

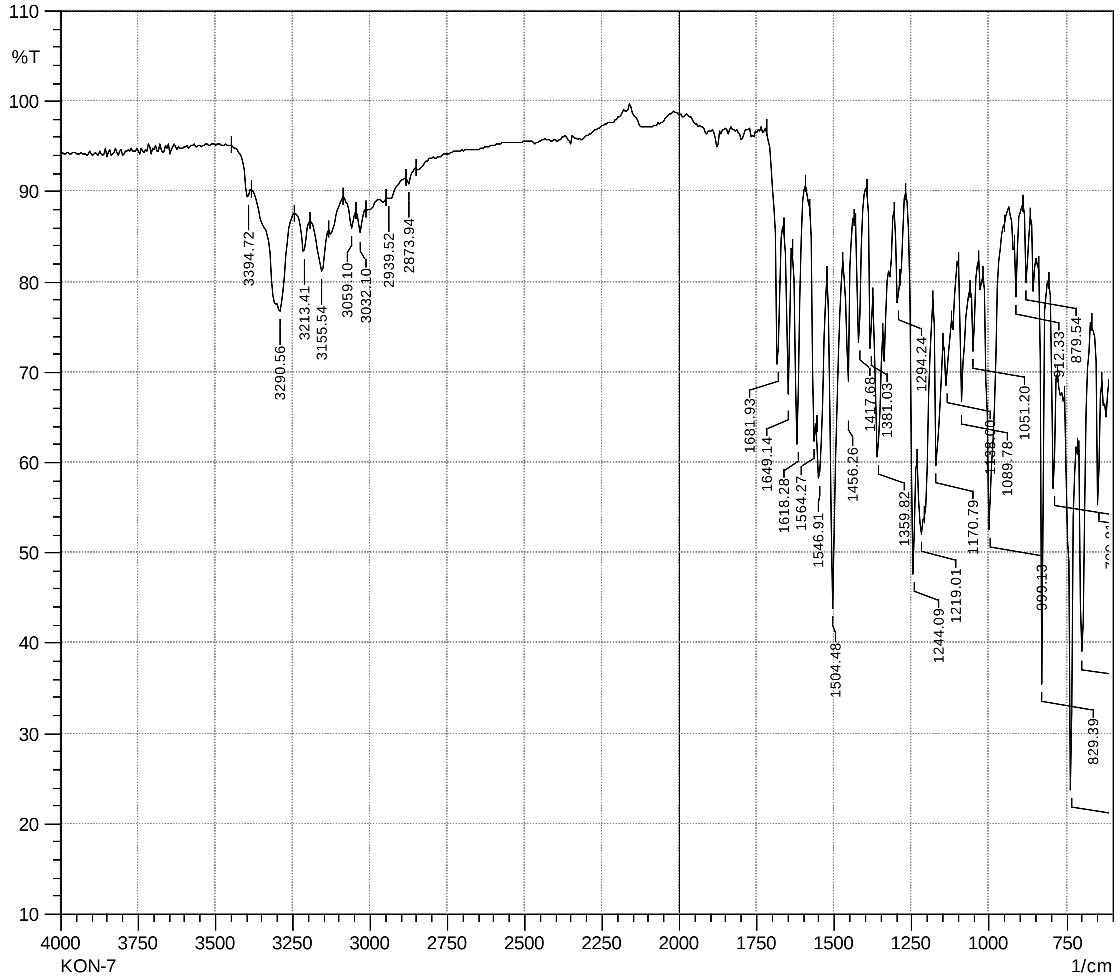

**Figure S66.** $^{1}$H NMR spectrum of compound **4g**

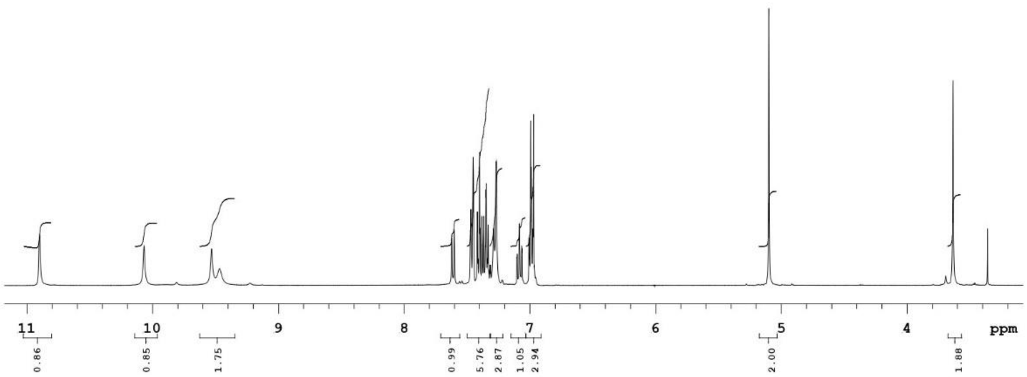


**Figure S67.** $^{13}$C NMR spectrum of compound **4g**

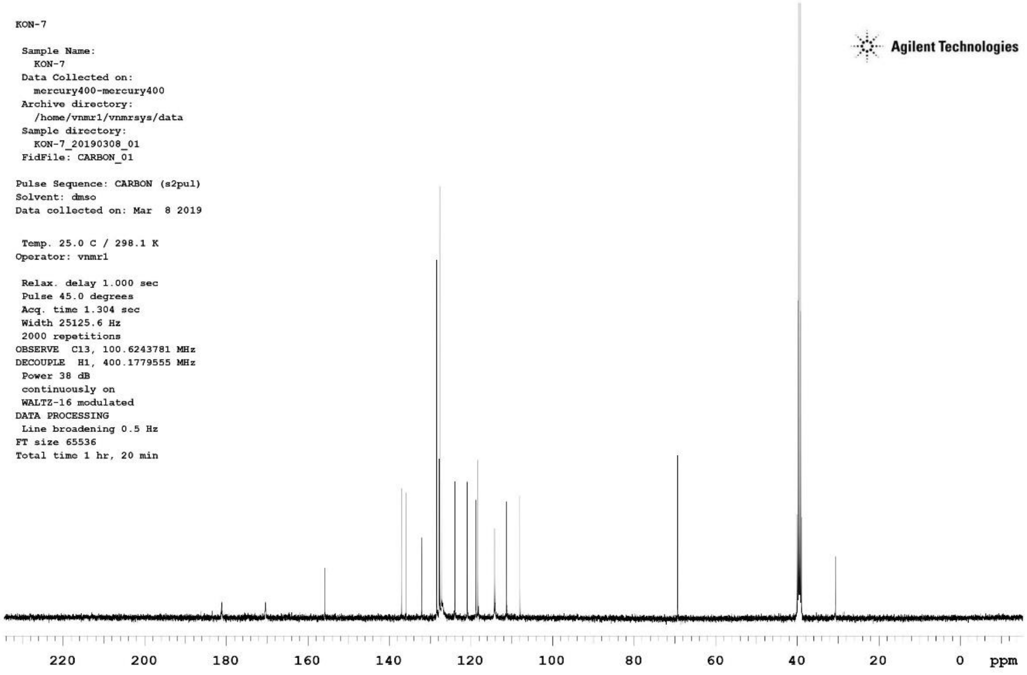

**Figure S68.** HRMS spectrum of compound **4g**

Formula Predictor Report - KON-7_59.lcd

Page 1 of 1

Data File: C:\LabSolutions\Data\Analiz\AOzdemir\KON-7_59.lcd

| Elmt | Val. | Min | Max | Elmt | Val. | Min | Max | Elmt | Val. | Min | Max | Elmt | Val. | Min | Max | Use Adduct |
|---|---|---|---|---|---|---|---|---|---|---|---|---|---|---|---|---|
| H | 1 | 10 | 40 | O | 2 | 1 | 5 | S | 2 | 1 | 2 | Ru | 2 | 0 | 0 | H |
| C | 4 | 18 | 30 | F | 1 | 0 | 0 | Cl | 1 | 0 | 0 | Pd | 2 | 0 | 0 | |
| N | 3 | 4 | 7 | P | 3 | 0 | 0 | Br | 1 | 0 | 1 | I | 3 | 0 | 0 | |

Error Margin (ppm): 20
HC Ratio: unlimited
Max Isotopes: 3
MSn Iso RI (%): 10.00

DBE Range: 13.0 - 21.0
Apply N Rule: yes
Isotope RI (%): 1.00
MSn Logic Mode: AND

Electron Ions: both
Use MSn Info: yes
Isotope Res: 9000
Max Results: 500

Event#: 1 MS(E+) Ret. Time : 3.733 -> 3.867 Scan# : 561 -> 581

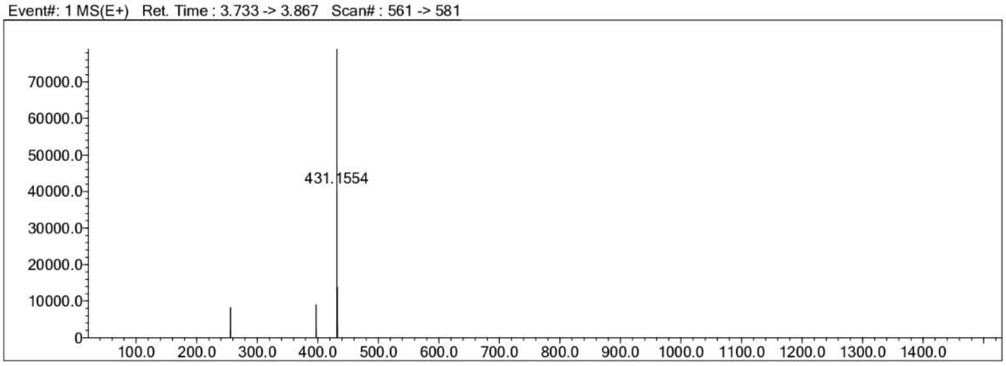


Measured region for 431.1554 m/z

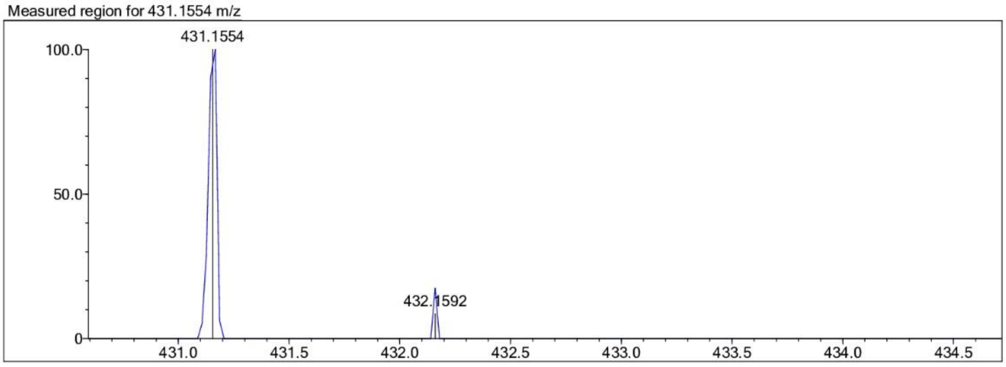


C24 H22 N4 O2 S [M+H]+ : Predicted region for 431.1536 m/z

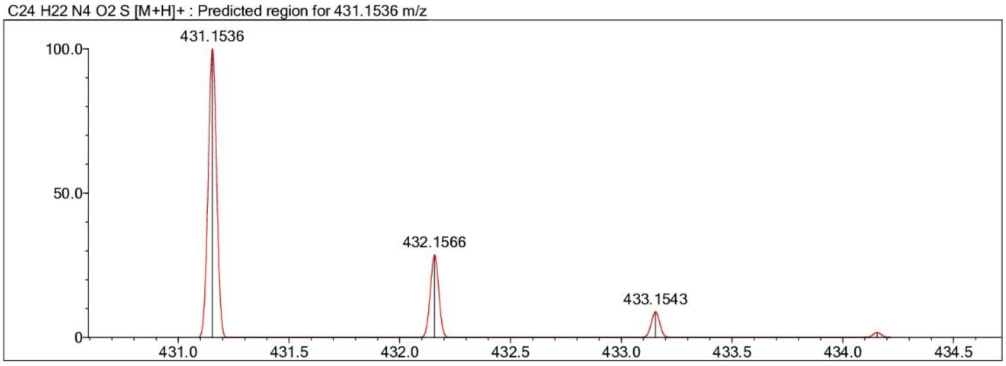


| Rank | Score | Formula (M) | Ion | Meas. m/z | Pred. m/z | Df. (mDa) | Df. (ppm) | Iso | DBE |
|---|---|---|---|---|---|---|---|---|---|
| 1 | 0.00 | C24 H22 N4 O2 S | [M+H]+ | 431.1554 | 431.1536 | 1.8 | 4.17 | 0.00 | 16.0 |
